# Implicit bias in ICU electronic health record data: measurement frequencies and missing data rates of clinical variables

**DOI:** 10.1186/s12911-025-03058-9

**Published:** 2025-07-01

**Authors:** Junming Shi, Alan E. Hubbard, Nicholas Fong, Romain Pirracchio

**Affiliations:** 1 Division of Biostatistics, University of California Berkeley, Berkeley, CA USA; 2 Department of Anesthesia and Perioperative Care, Zuckerberg San Francisco General Hospital and Trauma Center, 1001 Potrero Avenue, CA94110, San Francisco, CA USA

**Keywords:** Bias detection, Critical care, Data completeness, Electronic health records (EHRs), Health equity, Healthcare applications, Implicit bias, Measurement frequency, MIMIC-III dataset, Systematic disparities

## Abstract

**Background:**

Systematic disparities in data collection within electronic health records (EHRs), defined as non-random patterns in the measurement and recording of clinical variables across demographic groups, can be reflective of underlying implicit bias and may affect patient outcome. Identifying and mitigating these biases is critical for ensuring equitable healthcare. This study aims to develop an analytical framework for measurement patterns, defined as the combination of measurement frequency (how often variables are collected) and missing data rates (the frequency of missing recordings), evaluate the association between them and demographic factors, and assess their impact on in-hospital mortality prediction.

**Methods:**

We conducted a retrospective cohort study using the Medical Information Mart for Intensive Care III (MIMIC-III) database, which includes data on over 40,000 ICU patients from Beth Israel Deaconess Medical Center (2001–2012). Adult patients with ICU stays longer than 24 h were included. Measurement patterns, including missing data rates and measurement frequencies, were derived from EHR data and analyzed. Targeted Machine Learning (TML) methods were used to assess potential systematic disparities in measurement patterns across demographic factors (age, gender, race/ethnicity) while controlling for confounders such as other demographics and disease severity. The predictive power of measurement patterns on in-hospital mortality was evaluated.

**Results:**

Among 23,426 patients, significant demographic systematic disparities were observed in the first 24 h of ICU stays. Elderly patients (≥ 65 years) had more frequent temperature measurements compared to younger patients, while males had slightly fewer missing temperature measurements than females. Racial disparities were notable: White patients had more frequent blood pressure and oxygen saturation (SpO2) measurements compared to Black and Hispanic patients. Measurement patterns were associated with ICU mortality, with models based solely on these patterns achieving an area under the receiver operating characteristic curve (AUC) of 0.76 (95% CI: 0.74–0.77).

**Conclusions:**

This study underscores the significance of measurement patterns in ICU EHR data, which are associated with patient demographics and ICU mortality. Analyzing patterns of missing data and measurement frequencies provides valuable insights into patient monitoring practices and potential systemic disparities in healthcare delivery. Understanding these disparities is critical for improving the fairness of healthcare delivery and developing more accurate predictive models in critical care settings.

**Clinical trial number:**

Not applicable.

**Supplementary Information:**

The online version contains supplementary material available at 10.1186/s12911-025-03058-9.

## Introduction

The digitization of health records through Electronic Health Records (EHRs) has supplanted traditional paper-based systems, centralizing patient-specific information in an electronic medium. Over the past decade, several de-identified EHR datasets were made available to the public, mainly for research purposes. Notable examples include the MIMIC Database [1], PCORnet [2], I2B2 Data [3], and the COVID-19 Research Database.

The advent of large-scale, accessible EHR databases has led to a surge in studies to improve healthcare delivery by identifying patient phenotypes [4], developing risk prediction models [5], or enriching our understanding of risk factors in relation to health outcomes [6]. These real-world datasets not only reflect how care is delivered but also offer valuable insights into measurement patterns, specifically how often variables are measured or missing.

Previous research [7–11] has emphasized the necessity of addressing the issue of *missing values* and has recognized that incomplete data is often related to clinical practice, rather than being truly random. Common methods to handle missing data include imputation techniques [12], Inverse Probability Weighting (IPW) [13], and ignoring missing observations through complete case analysis (CCA) or available case analysis (ACA). While these methods are widely used, they have drawbacks, including the risk of compromised generalizability and the potential for increased bias due to their implicit modeling assumptions. Furthermore, they overlook the informative potential of missing data. This becomes especially problematic when data are not missing at random, as has been observed in Intensive Care Unit (ICU) settings [14, 15].

Similarly, the concept of *measurement frequency* is often overlooked in studies using real-world EHR data, despite its critical importance, especially in the ICU [16]. Measurement frequency refers to how often data points, such as vital signs or laboratory values, are collected. The frequency of vital sign collection can vary, and incorporating this variability can improve the performance of outcome prediction models [17]. While prior research has primarily focused on adjusting for or modeling with missing data, systemic patterns in measurement frequency and their implications for equity in care are rarely explored. In this study, we define measurement patterns as the combined consideration of missing data rates (the number of expected observations absent over time) and measurement frequencies (the number of observations recorded over time).

We hypothesize that, for both missing data rates and measurement frequencies, the underlying mechanisms are not random but rather reflective of systemic disparities in data collection practices, and that these measurement patterns are associated with patient outcomes. Specifically, we propose (i) to investigate the association between demographic variables, such as age, gender, and race/ethnicity, and measurement patterns of key clinical and biological parameters in critical care patients; (ii) to study whether such variations in measurement patterns, which are potentially arising from systematic disparities, are associated with in-hospital mortality. Our findings reveal implicit biases and systemic patterns within ICU EHR data, reflecting differences in vital sign monitoring and laboratory testing practices across demographic groups.

## Methods

### Data source

The data used in this study were sourced from the Medical Information Mart for Intensive Care III (MIMIC-III) [1], the most frequently used ICU EHR dataset and one of the very few publicly available datasets where protected attributes, such as demographic variables, are collected and documented [18].

MIMIC-III is a cohort of more than 40,000 de-identified patients who were admitted to ICUs at Beth Israel Deaconess Medical Center in Boston, Massachusetts, from 2001 to 2012, and is publicly accessible through PhysioNet [19]. MIMIC-III contains patient-level healthcare information, including demographics, hospital mortality, diagnostic data, laboratory tests, prescriptions, and medical procedures. Physiological signals (e.g., heart rate, blood pressure, oxygen saturation) are captured directly from bedside monitors, time-stamped, nurse-verified, and integrated into the EHR system, reducing variability in data collection [1]. This integration allows us to reasonably assume that the recorded measurements reflect actual clinical practice.

We included the first ICU admission of patients who met the following criteria: (1) age 18 years or older; (2) no ICU admissions related to pregnancy, childbirth, or postpartum; (3) no live discharge within the initial 24-hour period following admission; and (4) presence of at least one arterial line during the stay, ensuring continuous availability of vital signs [20]. A total of 23,426 patients met these criteria after excluding 23,094 patients who did not meet any of the above conditions. Specific exclusions included 7939 patients younger than 18 years, 17 patients with admissions related to pregnancy, 10,232 patients discharged within the initial 24 h, and 25,606 patients without arterial lines.

### Variables and data structure

We extracted data on 11 vital signs and 35 laboratory tests, selected from the top 80% of the most commonly performed tests [21] (Appendix A, Table S1), as well as baseline characteristics and severity scores for each ICU stay [22]. Data was extracted from the first five days of ICU stays, and segmented into consecutive 12-hour intervals. Further details on data structure are available in Appendix B.

### Measurement patterns

Two distinct types of measurement pattern variables were developed and analyzed: measurement frequency and missing data rate. Measurement frequency represents the total number of observations per variable during the first 24 h or subsequent 12-hour blocks. Since there is no standard protocol for measuring all vital signs and laboratory tests uniformly across ICUs, we used measurement frequency as an indicator of monitoring intensity.

Missing data rate, on the other hand, quantifies the number of hours without an observation for a given vital sign. For continuously monitored vital signs, which are expected to be recorded hourly by nurse [1], an hour-long gap without a recorded value was classified as missing data. For example, if no temperature measurement is recorded during the third hour of a patient’s ICU stay, this hour would be classified as missing data.

For laboratory tests, which are ordered on an as-needed basis and lack a standardized collection schedule, missing data was not explicitly defined. Instead, measurement frequency was used to capture the ordering patterns of laboratory test variables. To capture missing data patterns for vital signs, we calculated the total hours with missing data for each patient during the analysis periods. For the first 24-hour block, the missing data rate can range from 0 to 24, and for the 12-hour blocks, from 0 to 12. Detailed methods for calculating measurement patterns are described in Appendix C.

Since some vital signs are often measured together (e.g., systolic, diastolic, and mean blood pressures), we grouped them and consolidated their missingness rate and measurement frequency into average rates. Similarly, for laboratory tests that are ordered together (e.g., complete blood count), we grouped them and consolidated their measurement rates into single variables that reflect group averages, as detailed in Appendix C, Table S2.

### Statistical analysis

#### Association of demographic variables and measurement patterns

To estimate the association between demographic variables and measurement patterns, we used the double robust Targeted Maximum Likelihood Estimator (TMLE) [23]. This nonparametric method provides efficient and doubly robust estimates of causal parameters, while rigorously addressing high-dimensional intermediating and confounding covariates. Specifically, TMLE trains the outcome model and treatment model (propensity score) with confounding variables, and then updates the initial outcome model using a clever covariate derived from the propensity score to reduce bias.

Given the unknown data distribution inherent in observational studies and the model selection flexibility allowed in TMLE, we utilized Super Learner (SL) [24] algorithm to estimate the outcome and treatment models. SL is an ensemble machine learning method that applies cross-validation to select the best-performing model among a set of candidate algorithms. By leveraging the SL framework, we maximized the model’s ability to account for associations between outcomes and covariates, isolating the independent effect of demographic factors. To account for intra-patient correlation stemming from multiple ICU admissions, we implemented stratified cross-validation.

In the TMLE analysis, the measurement pattern variable served as the outcome. We assessed the association between each demographic variable and the outcome, while adjusting for other demographic factors and clinical severity scores, including the Sequential Organ Failure Assessment (SOFA) score and Simplified Acute Physiology Score (SAPS) II. For example, we estimated the expected number of heart rate measurements taken over the initial 24 h for each racial/ethnic groups. Potential confounding variables, which are severity scores and other demographic variables including age, gender, insurance status, language, religion, and marital status, were incorporated in both initial outcome and treatment models within the TMLE analysis. Adjusted averages for measurement patterns were compared between groups, and pairwise hypothesis testing was conducted to assess whether the values were drawn from the same distribution. P-values were computed using the estimated standard errors derived from the TMLE analysis.

Furthermore, we conducted several sensitivity analyses utilizing more traditional regression methods, including generalized linear models (GLMs), to assess the robustness of our findings. These analyses examined both unadjusted and adjusted coefficients for the association between demographic variables and measurement pattern variables across different model specifications.

#### Association of measurement patterns and mortality

We investigated the relationship between measurement patterns and patient outcomes by predicting ICU mortality within the next 12 h and evaluating their predictive capacities. Using the SL algorithm, we compared the predictive performance of three models: (1) based solely on clinical values (e.g., baseline characteristics, vital sign values, and laboratory test results), (2) based solely on measurement patterns including measurement frequencies and missing data rates, and (3) a combined model using both clinical values and measurement patterns. We implemented 10-fold cross-validation, stratified by patient ID to account for repeated measures and avoid overlap between training and validation sets. The SL library included models such as Generalized Linear Models (GLM), Bayesian GLM, Generalized Additive Models (GAM), Ridge Regression, ElasticNet, Lasso Regression, Random Forests, Gradient Boosting Machines, and Bayesian Additive Regression Trees.

In models (1) and (3), we imputed missing values using the median for continuous variables and the mode for categorical variables. Predictive performance was assessed using Negative Log-Likelihood (NLL), area under the receiver operating characteristic curve (AUC), and Area Under the Precision-Recall Curve (AUCPR), to compare the effectiveness of each model in predicting ICU mortality. Specifically, AUCPR is useful for imbalanced datasets as it highlights the trade-off between precision and recall for the minority class.

All analyses were performed using the R software version 4.3.1 (2023-06-16), utilizing the sl3 (SuperLearner) and tmle3 (TMLE) packages [25].

## Results

### Patient demographics

Of 23,426 patients who met our inclusion criteria for the study, 464 patients (2.02%) experienced mortality within the first 5 days of their ICU stay. Patients’ characteristics are summarized in Table 1.

**Table 1:** Demographic breakdown and severity scores summary from the datasets used in the MIMIC-III

Demographics		No ICU Mortality (*N* = 22962)	ICU Mortality (*N* = 464)	Overall (*N* = 23426)
Age	Mean (SD)	64.8 (17.2)	67.0 (18.2)	64.8 (17.2)
	Median (IQR)	66.0 (23.0)	69.0 (25.0)	66.0 (23.0)
Gender	Female	9531 (41.5%)	210 (45.3%)	9741 (41.6%)
	Male	13,431 (58.5%)	254 (54.7%)	13,685 (58.4%)
Religion	Christian	11,671 (50.8%)	198 (42.7%)	11,869 (50.7%)
	Not Christian	3509 (15.3%)	56 (12.1%)	3565 (15.2%)
	Missing	7782 (33.9%)	210 (45.3%)	7992 (34.1%)
Partner	Partner	11,576 (50.4%)	208 (44.8%)	11,784 (50.3%)
	No Partner	9869 (43.0%)	180 (38.8%)	10,049 (42.9%)
	Missing	1517 (6.6%)	76 (16.4%)	1593 (6.8%)
Language	English	11,798 (51.4%)	206 (44.4%)	12,004 (51.2%)
	Not English	1879 (8.2%)	46 (9.9%)	1925 (8.2%)
	Missing	9285 (40.4%)	212 (45.7%)	9497 (40.5%)
Ethnicity	White	16,300 (71.0%)	306 (65.9%)	16,606 (70.9%)
	Black	1487 (6.5%)	25 (5.4%)	1512 (6.5%)
	Hispanic	674 (2.9%)	17 (3.7%)	691 (2.9%)
	Asian	497 (2.2%)	14 (3.0%)	511 (2.2%)
	Other	653 (2.8%)	13 (2.8%)	666 (2.8%)
	Missing	3351 (14.6%)	89 (19.2%)	3440 (14.7%)
Insurance	Yes	22,716 (98.9%)	449 (96.8%)	23,165 (98.9%)
	No	246 (1.1%)	15 (3.2%)	261 (1.1%)
SOFA	Mean (SD)	4.69 (3.11)	7.50 (4.52)	4.75 (3.17)
	Median (IQR)	4.00 (4.00)	7.00 (7.00)	4.00 (4.00)
SAPS II	Mean (SD)	36.8 (14.1)	52.8 (17.4)	37.1 (14.4)
	Median (IQR)	35.0 (18.0)	51.0 (25.3)	35.0 (18.0)

### Differential measurement pattern

**Fig. 1:**
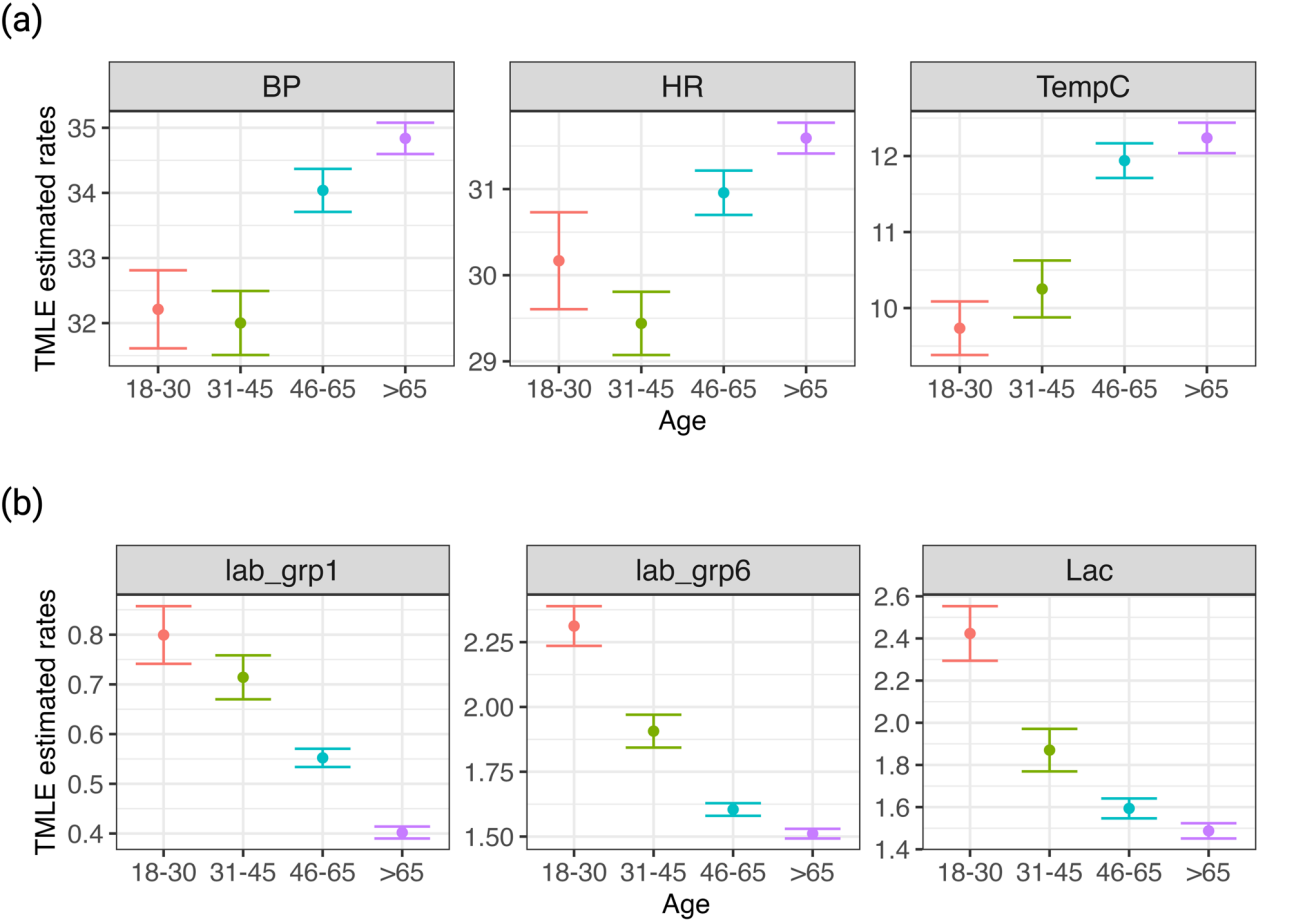
TMLE Estimated Measurement Patterns by Age Groups. This figure illustrates TMLE-estimated marginal average measurement frequencies across four age groups, considering baseline characteristics and SOFA scores. It includes three vital signs and three laboratory tests, showcasing implicit biases in monitoring. Full details and additional plots are available in Appendix D. (**a**) Marginal average measurement frequencies of vital signs among age groups. (**b**) Marginal average laboratory testing frequencies among age groups

Our analysis first focused on the initial 24 h of ICU data, comprising 24,517 ICU stays. After adjusting for severity scores and demographic variables, statistically significant differences in measurement patterns were observed across different age groups (Fig. 1). Elderly patients, particularly those aged 46–65 and over 65, received more frequent vital sign measurements and had fewer missing measurements compared to younger patients, except for the Glasgow Coma Scale (GCS). For example, the adjusted average number of temperature measurements in patients aged 65 and older was 12.2 (95% CI: 12.0 to 12.4), while patients aged 18–30 had 9.7 measurements (95% CI: 9.4 to 10.1) (*p* < 0.001). The adjusted average missing data rates were 14.72 h (61.3% of the initial 24 h) (95% CI: 14.61 to 14.83) for patients ≥ 65 and 15.45 (64.4%) (95% CI: 15.21 to 15.69) for patients aged 18–30 (*p* < 0.001) (Fig. 1). However, laboratory test frequencies decreased linearly with increasing age, a trend consistent across all tests, even after adjusting for SOFA scores and other covariates (Appendix D.1).

**Fig. 2:**
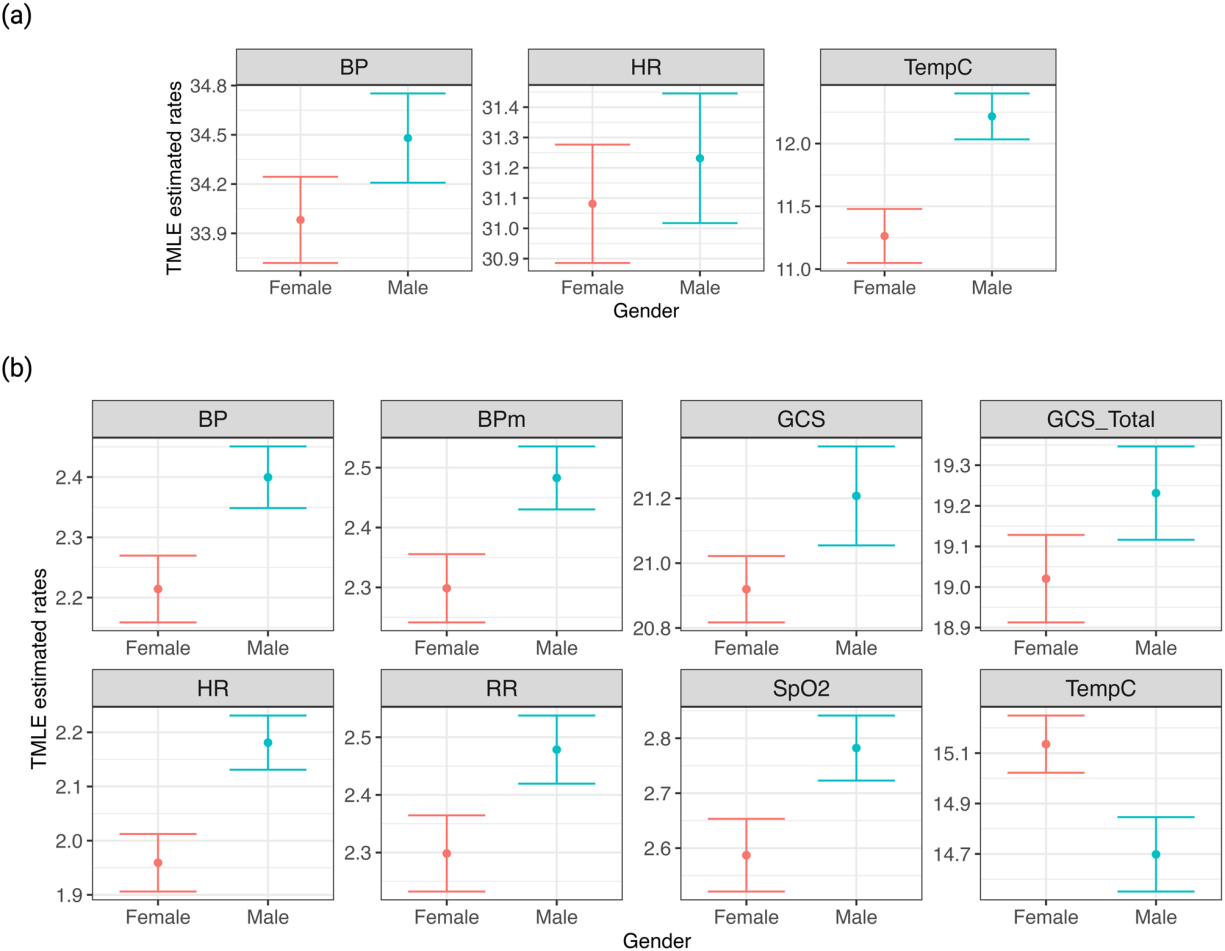
TMLE Estimated Measurement Patterns by Gender. This figure displays TMLE-estimated measurement patterns for female and male patients, incorporating baseline characteristics and SOFA scores. It focuses on three vital signs, highlighting gender-based differences in patient monitoring. (**a**) Marginal average measurement frequencies of vital signs among males and females. (**b**) Marginal average number of hours with missing vital signs among males and females

From gender-based analysis, males had approximately 0.8% more missing vital sign measurements than females, although no significant differences in measurement frequencies were noted. An exception was temperature monitoring, where males received marginally one more check (12.22, 95% CI: 12.03 to 12.40) and had 1.8% fewer hours of missing data (14.69 h (61.2%), 95% CI: 14.55 to 14.85) compared to females, who had on average 11.26 measurements (95% CI: 11.05 to 11.50) (*p* < 0.001) and 15.13 h (63%) (95% CI: 15.02 to 15.25) (*p* < 0.001) of missing measurements (Fig. 2). While there were some differences in laboratory test frequencies by gender, these were inconsistent across different test groups (Appendix D.2).

**Fig. 3:**
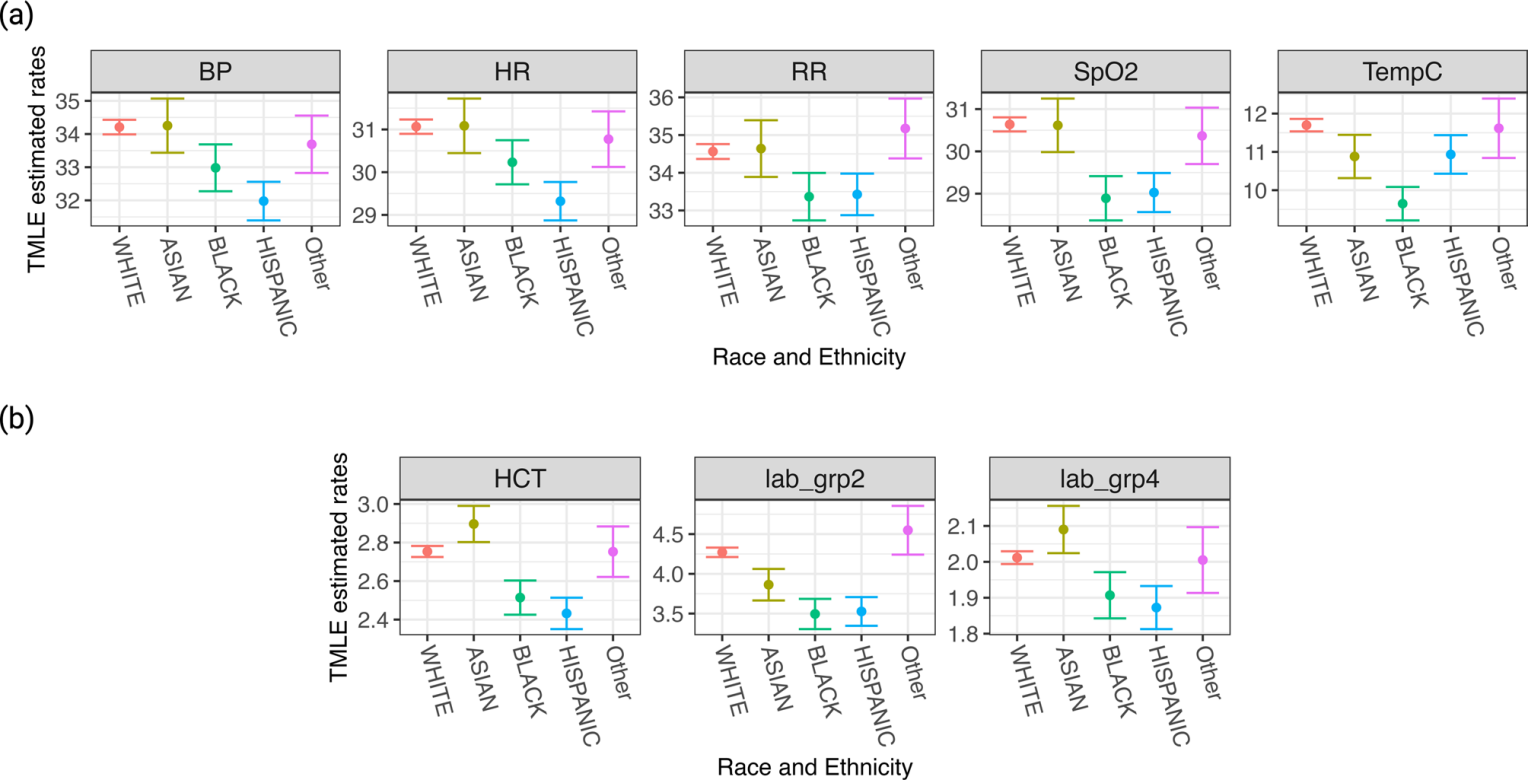
TMLE Estimated Measurement Patterns by Race/Ethnicity. Displaying TMLE-estimated marginal average measurement frequencies for different racial and ethnic groups. The top plot shows five vital signs and the bottom plot shows three laboratory tests, revealing racial and ethnic disparities in monitoring within healthcare settings. (**a**) Marginal average measurement frequencies of vital signs among race/ethnic groups. (**b**) Marginal average measurement frequencies of lab testing among race/ethnic groups

Racial and ethnic differences were also observed. Black and Hispanic patients had fewer vital sign measurements compared to White patients, although no significant differences were seen in the rate of missing data. For instance, the adjusted average number of blood pressure measurements within the first 24 h was 34.22 (95% CI: 34.00 to 34.44) for White patients, compared to 32.79 (95% CI: 32.11 to 33.46) for Black patients and 32.39 (95% CI: 31.82 to 32.98) for Hispanic patients (p-values < 0.001 for the comparisons between White and Black, and White and Hispanic) (Fig. 3). Notable differences were also observed in SpO2 and temperature, both in measurement frequencies and missing data rates. For SpO2, the adjusted average number of measurement s for White patients was 30.64 (95% CI: 30.48 to 30.81) with 2.60 h (10.8%) (95% CI: 2.55 to 2.65) of adjusted average missing data rates, compared to 28.83 measurements (95% CI: 28.32 to 29.34) (*p* < 0.001) and 3.09 h (12.9%) (95% CI: 2.85 to 3.32) (*p* < 0.001) for Black patients. Similar trends were seen for temperature measurements: White patients had an average of 11.70 measurements (95% CI: 11.54 to 11.86) and 14.90 h (62.1%) (95% CI: 14.79 to 15.01) of adjusted average missing data rates, compared to 9.65 measurements (95% CI: 9.21 to 10.08) (*p* < 0.001) and 15.82 h (65.9%) (95% CI: 14.54 to 16.0) (*p* = 0.0147) for Black patients. Laboratory test patterns did not show consistent disparities across racial/ethnic groups, although hematocrit tests revealed small but statistically significant differences (2.75 [95% CI: 2.73 to 2.78] for White patients vs. 2.49 [95% CI: 2.40 to 2.57] for Black patients, *p* < 0.001) (Fig. 3).

**Fig. 4:**
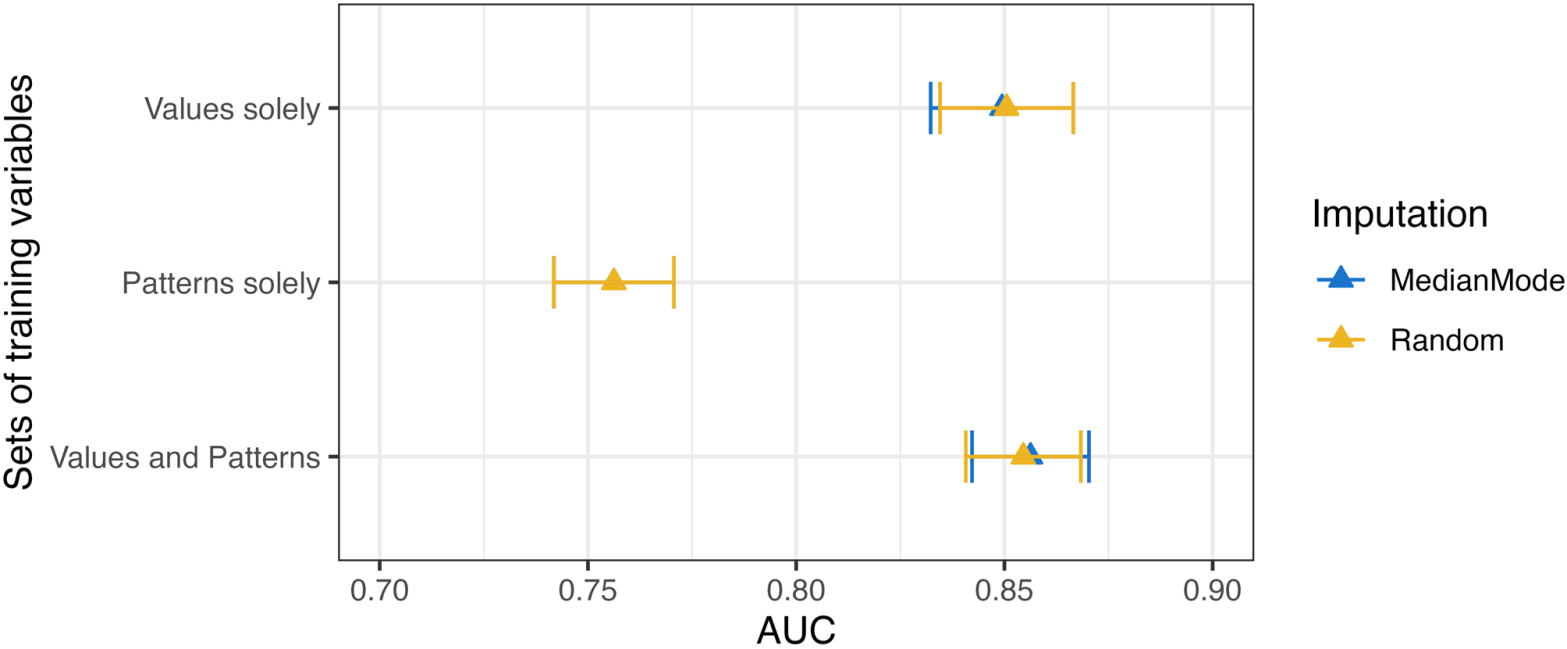
Area Under the Receiver Operating Characteristic Curve for mortality prediction

Other demographic factors, including insurance status, language, marital status, and religion, did not exhibit consistent patterns in measurement frequency (Appendix D). Sensitivity analyses confirmed the robustness of these findings, with consistent trends observed across unadjusted and adjusted models (Appendix G). For instance, in the sensitivity analysis, the expected measurement frequency for patients aged 65 and older was 12.5, compared to 12.2 in the primary analysis. Similarly, for patients aged 18–30, the expected measurement frequency was 9.7 in the primary analysis, compared to 8.9 in the unadjusted sensitivity analysis (See Fig. 4).

### Association of measurement patterns and mortality

The 12-hour mortality prediction model trained on both count variables (measurement frequency and missing data rate) and original value variables demonstrated the highest predictive accuracy, achieving an AUC of 0.86 (95% CI: 0.84 to 0.87) and an AUCPR of 0.19 (95% CI: 0.17, 0.22) on the CV testing sets, and both AUC and AUPRC of 1.0 for the full-fit training set (Figure 4, Table 2, Appendix E). This performance was comparable to the model trained solely on original value variables, which achieved an AUC of 0.85 (95% CI: 0.83 to 0.87) and an AUCPR of 0.18 (95% CI: 0.16, 0.20) for the CV testing set, and both AUC and AUCPR of 1.0 for the full-fit training set. Although the difference between these two models was not statistically significant, measurement patterns, which are often overlooked compared to clinical diagnosis and treatment, exhibited a discernible impact on mortality prediction. Notably, the model trained exclusively on measurement frequency and missing data rate variables show meaningful predictive power on its own, achieving an AUC of 0.76 (0.74, 0.77) and an AUCPR of 0.10 (95%CI: 0.09, 0.11) for the CV testing sets, and an AUC of 1.0 and AUCPR of 0.994 for the full fit training set. This result is much higher than the 0.019 AUCPR expected from a random guess in this imbalanced dataset.

**Table 2:** Performance metrics of random forest models across different variable combinations. This table summarizes the average negative Log-Likelihood (NLL), area under the ROC curve (AUC), and area under the Precision-Recall curve (AUCPR) for random forest models trained with different variable sets

Training	Full Fits			Cross-Validation Testing Sets		
Variables	AUC	AUCPR	NLL	AUC	AUCPR	NLL
Value	1.0	1.0	0.025	0.85 (0.83, 0.87)	0.18 (0.16, 0.20)	0.17 (0.16, 0.19)
Pattern	1.0	0.994	0.033	0.76 (0.74, 0.77)	0.10 (0.09, 0.11)	0.21 (0.18, 0.23)
Value & Pattern	1.0	1.0	0.025	0.86 (0.84, 0.87)	0.19 (0.17, 0.22)	0.17 (0.15, 0.19)

## Discussion

This study offers a novel perspective on measurement patterns within EHR for ICU patients, revealing systemic disparities in ICU monitoring and data collection practices. Even after aggressively adjusting for baseline clinical health measures, we found statistically significant disparities in the frequency of vital sign and laboratory test measurements and missing data rates across different demographic groups during the first 24 h of ICU admission. Importantly, the observed systematic disparities in measurement patterns were found to be associated with ICU mortality.

While our findings provide robust evidence of systemic disparities in ICU data collection, the generalizability of these findings is limited because the data analyzed originated from the MIMIC-III database, which represents a single healthcare system. However, many of our results align with prior disparities research that has documented demographic differences in healthcare delivery. We found systemic racial disparities, with Black and Hispanic patients receiving significantly fewer vital sign measurements compared to White patients. This aligns with previous studies showing that racial and ethnic minoritized groups often receive fewer, lower-quality, and less timely diagnostic procedures and more conservative treatment interventions [26–30].

We also found that males experienced slightly more frequent temperature checks than females, a pattern consistent with documented gender disparities in healthcare delivery and representation in medical research [31–34]. Moreover, we found that older patients received fewer laboratory tests than the younger patients but underwent more frequent vital sign measurements with fewer gaps in data collection, except for GCS measurements. This observation contrasts with reports of healthcare ageism in non-ICU settings [35, 36] and highlights the complex relationship between aging and disparities in healthcare delivery, which continues to be explored in the literature [37]. While we observed a linear decrease in the frequency of some laboratory tests with increasing age, no consistent disparities in laboratory testing were observed across other demographic groups. Additional factors, including insurance status, language, marital status, religion, and care unit, were explored but did not exhibit strong patterns in our analysis.

Although the study is based on a single database, the most widely used database in the literature, the theoretical implications of our findings are significant. Biases in measurement patterns, especially in ICUs, may reflect broader systemic issues such as staffing ratios or implicit prioritization strategies and may arise in other ICU health systems due to shared systemic factors. However, the specific manifestations of these biases may vary depending on the patient population, the technology and protocols governing data collection, and regional health care policies.

In the MIMIC-III database, physiologic variables, such as heart rate, represent nurse-verified vital signs recorded approximately hourly. This verification step introduces variability in recorded data because it depends on clinical workflows, staffing levels, and adherence to hourly documentation practices. As a result, recorded measurement frequencies may not always align with planned frequencies, particularly due to manual data collection processes. For instance, in systems with lower staffing levels or during acute crises, delays in nurse verification may lead to gaps or inconsistencies in recorded vital sign data, even for continuously monitored variables. Further research across diverse healthcare settings is needed to determine how widely these findings apply.

Furthermore, although we only assessed ICU mortality, these disparities in monitoring are likely to influence not only mortality but also other critical outcomes, such as infections, bleeding or clotting events, arrhythmias, length of ICU stay, and hospital or 30-day mortality. For example, in our additional analysis, we found that vital sign measurement patterns are associated with length of ICU stay (Appendix G).

Our findings contribute to the growing body of literature highlighting biases in healthcare data analytics by uniquely examining measurement patterns including missing data rates and measurement frequencies, providing a comprehensive review of systemic disparities in ICU data collection practices, and demonstrating the predictive value of measurement patterns for patient outcomes, rather than simply adjusting for these factors. These results align with prior research identifying significant sampling biases and missing data in healthcare data and medical algorithms, which can skew results and perpetuate inequalities [37–42]. This distinct focus on the implications of variability in measurement practices and missing values emphasizes the importance of accounting for these factors when analyzing and modeling critical care data to ensure more equitable and accurate insights.

### Limitations

This study has several limitations. First, the data originate from the MIMIC-III database, which represents a single healthcare system and predominantly consists of older and White patients which may limit the generalizability of findings. However the inclusion of hundreds of patients from minoritized groups provides sufficient statistical power for meaningful analysis. In addition, TMLE adjustments mitigate potential biases from uneven data distribution, ensuring valid comparisons across demographic groups. Second, measurement frequencies in this study reflect recorded data rather than planned or intended measurements. For physiologic variables in MIMIC-III, nurse-verified documentation introduces variability in recorded measurement frequency, even for continuously monitored vital signs. Manual processes and staffing levels may impact whether measurements are performed as planned, introducing potential variability in recorded frequencies. Additionally, our analysis assumes an hourly baseline expectation for continuously monitored variables, which may not fully capture variations in clinical needs during acute crises. This assumption likely does not affect our overall conclusions but adds nuances that merit further consideration. Third, our prediction analysis supports the conceptual link between measurement patterns and ICU mortality. However, additional statistical analyses examining whether measurement patterns mediate the relationship between patient demographics and hospital mortality are needed to confirm this conceptual link. Lastly, while TMLE adjustments and robust statistical methods mitigate biases, unmeasured confounders could still influence results.

### Implications and recommendations

Healthcare systems need to routinely audit their monitoring and data collection practices and address any disparities that may arise. By identifying and rectifying systemic patterns of bias, institutions can create a more equitable healthcare data landscape. Furthermore, EHR data should be analyzed and modeled with an acute awareness of potential biases. We caution against relying solely on advanced imputation techniques without first understanding the underlying measurement patterns. Imputation methods should be applied with careful consideration of demographic characteristics to ensure fair representation. Specifically, advanced imputation techniques such as multiple imputation by chained equations (MICE) and AI-based imputation methods can be adapted to account for the demographic factors that influence data collection [43, 44].

Furthermore, incorporating measurement patterns into statistical and machine learning models can mitigate the impact of varied measurement patterns. This can be achieved by weighting the samples and observations based on their measurement frequencies, modeling the data generation process as a multilevel model where the measurement pattern is treated as a latent variable, or employing a Bayesian model to estimate the impact of different measurement frequencies on model outcomes, thus providing a probabilistic framework to handle uncertainty [42, 45–47]. By incorporating these strategies, healthcare analytics can achieve greater fairness and accuracy.

## Conclusions

This study identified the significant systemic disparities in measurement patterns and data collection practices in ICU electronic health record (EHR) data. We found that demographic factors, including age, gender, and race/ethnicity, are associated with differences in measurement patterns, including both measurement frequency and missing data rates. Specifically, males underwent slightly more temperature checks than females, Black and Hispanic patients received fewer vital sign measurements compared to White patients, and older patients had fewer laboratory tests than younger patients but more frequent vital sign checks in the ICU.These variations in measurement patterns exhibited strong predictive power for ICU mortality suggesting a conceptual link between demographic disparities in monitoring and clinical outcomes.

Our findings emphasize the urgent need for healthcare systems to address disparities in patient monitoring, data collection, integrate measurement patterns into predictive modeling, and promote transparency in EHR practices. Such efforts are essential to advancing equitable care delivery and improving outcomes for all patients.

## Electronic supplementary material

Below is the link to the electronic supplementary material.

Supplementary Material 1

## Data Availability

The data that support the findings of this study are derived from the MIMIC-III database [15], which is publicly available but subject to specific access requirements. Access to MIMIC-III data requires users to complete a data use agreement and undergo a certification process, which includes completing the required training in human subjects research (CITI Program). While the data used in this study were obtained under this agreement and are not publicly available due to licensing restrictions, they may be made available by the authors upon reasonable request, contingent on approval from the MIMIC-III data custodians and adherence to the relevant data use agreements.

## Abbreviations

ICU: Intensive Care Unit
MIMIC-III: Medical Information Mart for Intensive Care III
EHR: Electronic Health Record
GCS: Glasgow Coma Scale
SOFA: Sequential Organ Failure Assessment
TMLE: Targeted Maximum Likelihood Estimator
SL: Super Learner
GLM: Generalized Linear Models
AUC: Area under the receiver operating characteristic curve
MICE: Multiple imputation by chained equations
