# Supplementary material for: Implicit bias in ICU electronic health record data: measurement frequencies and missing data rates of clinical variables
Source: BMC Med Inform Decis Mak. 2025 Jul 1;25:241. doi: 10.1186/s12911-025-03058-9 (PMC12220764; doi:10.1186/s12911-025-03058-9)
Supplement: Supplementary file 1 — Supplementary Material 1 [file 12911_2025_3058_MOESM1_ESM.docx]

### Appendix A. Extracted variable list from the HER

**Table S1: Extracted variable list from the EHR.** This table includes 7 baseline characteristics, 11 vital signs, and 35 laboratory tests. The laboratory tests were selected based on the top 80% of the most frequently ordered tests during ICU stays and the clinical expertise of Professor Romain Pirracchio, MD, PhD.

| **Baselines (W):**  • Age  • Gender  • Insurance  • Language  • Religion  • Marital status  • Ethnicity | **Vital Signs (VS):**  • Heart Rate (HR)  • Respiratory Rate (RR)  • O2 saturation pulseoxymetry (SpO2)  • Temperature Celsius (TempC)  • Blood Pressure Diastolic (BPd)  • Blood Pressure Systolic (BPs)  • Blood Pressure Mean (BPm)  • Glasgow Coma Scale Eye (GCS_Eye)  • Glasgow Coma Scale Verbal (GCS_Verbal)  • Glasgow Coma Scale Motor (GCS_Motor)  • Glasgow Coma Scale Total (GCS_Total) |
| --- | --- |
| **Lab Tests (LT):**  • Alanine aminotransferase (ALT)  • Alkaline phosphatase (ALK)  • Aspartate aminotransferase (AST)  • Base excess (BE)  • Lactate (Lac)  • Calcium (Ca)  • Magnesium (Mg)  • Phosphate (Phos)  • Partial thromboplastin time (PTT)  • Prothrombin time (PT)  • Total bilirubin (TBil)  • Arterial blood gases: pH (pH), partial pressure of carbon dioxide (PCO2), and partial pressure of oxygen (PO2)  • Basic metabolic panel: sodium (Na), potassium (K), chloride (Cl), bicarbonate (HCO3), anion gap (AG), blood glucose  (BG), blood urea nitrogen (BUN), and creatinine (Cr)  • Complete blood count: white blood cells (WBCs), red blood cells (RBCs), hemoglobin (HGB), hematocrit (HCT), mean  corpuscular volume (MCV), mean corpuscular hemoglobin (MCH), mean corpuscular hemoglobin concentration (MCHC),  red cell distribution width (RDW), platelet count (PLT), absolute monocytes (MO), absolute eosinophils (EO), absolute  basophils (BA), and absolute neutrophils (NE) | |

### Appendix B. Data Structure

We assume that the dataset comprises independent and identically distributed (i.i.d.) patients. Nominal variables within the data set are represented by dummy variables for analytical purposes. For each patient i in the dataset, the complete data is represented as (Wi, Tij , variable(Tij ), value(Tij )), for i = 1, ..., n and j = 1, ..., ni. Herein, Wi represents the baseline covariates, and Tij are the times where time-dependent variable variable(Tij ) is observed,

accompanying a specific measurement value(Tij ). The quantity ni is defined as the total count of measurements for the variable variable(Tij ) for the ith patient. The time-dependent variables are enumerated as variable(Ti.) ∈ ”HR”, ”RR”, ..., ”GCSTotal”, ”ALT”, ”ALK”, ..., ”NE”, ”Y ”. Within this set, ”HR”, ”RR”, ..., ”GCSTotal” are designated as abbreviations for seven selected vital signs, ”ALT”, ”ALK”, ..., ”NE” stand for abbreviations of thirty-five chosen laboratory tests, and Y represents the outcome of interest, namely ICU mortality.

In the dynamic environment of Intensive Care Units (ICUs), a patient’s condition can exhibit significant variability within short intervals. Given the diverse duration of ICU stays and our research goal, we extracted two datasets. The initial dataset encompasses the initial 24-hour period data for each patient, symbolized as Oi = (Wi, Xi), where Xi denotes the averaged values of vital signs and laboratory tests for patient i during the first 24 hours. Among cases that experienced mortality in the hospital, with ICU stays shorter than 24 hours, their instances have undergone standardization of their measurements.

Subsequently, the second dataset was formulated by segmenting the time into 12-hour intervals, resulting in a collection of observations Oi(b) = (Wi, Xi(b), Yi(b + 1)) for i = 1, ..., n, and b = 1, ..., bi. Here, b signifies each sequential 12-hour block, bi i is the total number of such blocks during a patient’s ICU stay until transfer or death, Xi are the average values of vital signs and laboratory tests for patient i in the 12-hour block b, and Yi(b + 1) denotes the subsequent ICU mortality outcome. Considering the variability in ICU stay lengths, the data includes at most the first ten blocks (120 hours), thus capping bi at ten. Incomplete time blocks, shorter than 12 hours due to discharges or in-hospital mortality, are excluded from the dataset. For instance, a patient who succumbs at the 30th hour in the ICU would have their data represented in two rows, corresponding to the first (0 - 12 hours) and second (12 - 24 hours) time blocks. Each row includes baseline covariates, average vital signs, and laboratory measurements for the block,

alongside the ICU mortality in the ensuing block. In this specific case, the outcome Yi(2) in the first row would be 0, and Yi(3) in the second row would be 1. The residual 6 hours (25 - 30 hours), constituting an incomplete time block, are omitted from the dataset.

### Appendix C. Detailed Statistical Analysis

C.1 Statistical Analysis

C.1.1 Missing data rates and measurement frequencies

In the absence of universally standardized frequencies for vital sign screening (VS) and laboratory testing (LT), quantifying the measurement patterns and the associated missing data becomes challenging. To address this, two distinct types of counting variables were devised. The first type is measurement frequency, which quantifies the total number of observations within a fixed length of time. This is expressed as
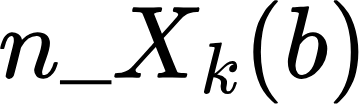
 or
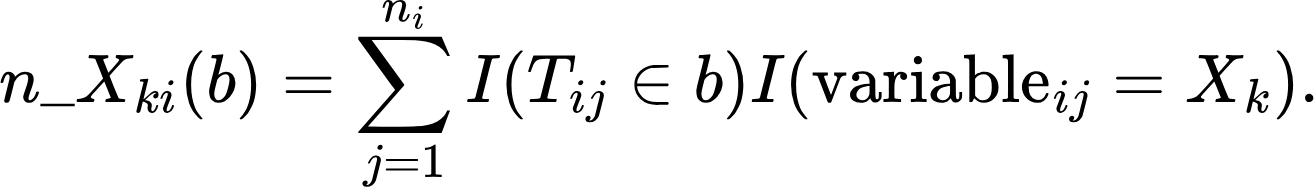


Acknowledging that groups of vital signs and laboratory tests are frequently ordered and measured concurrently, resulting in strongly correlated sampling frequencies, these groups of variables have been amalgamated into single variables representing group averages. The specifics of this grouping are detailed in Table 5. For baseline covariates, the notation
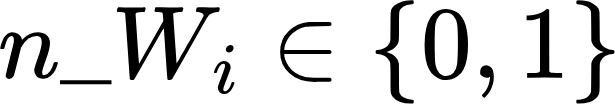
 is employed to signify the presence of baseline information.

The second type of counting variable is the missing data rate, which addresses the challenge of characterizing the missing data pattern of time-dependent variables, which are often recorded in a less structured manner. A summary measure, termed the missing data rate, was introduced to encapsulate the frequency of missing observations. This rate is calculated as the number of hours without observations per variable, both calculated for the initial 24-hour dataset and for the discretized 12-hour time blocks. Specifically, for a given covariate,
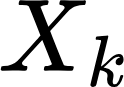
 , within a certain time block, b, the missing data rate is defined as
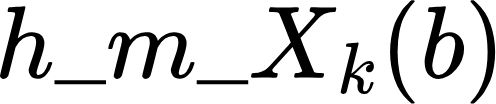
, or
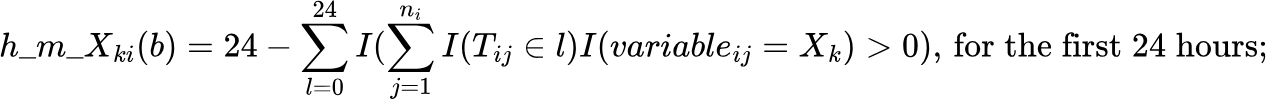


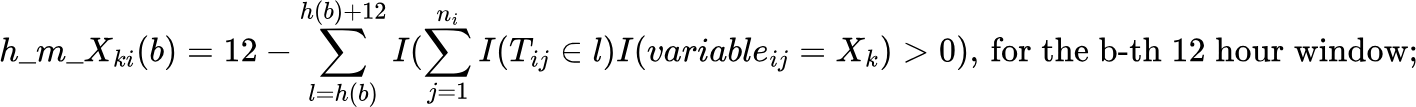


where h(b) represent the hour at the beginning of block, b. The missing data rate varies from 0 (indicating hourly monitoring within a time block) to 24 for the first 24-hour window or 12 for the 12-hour blocks (denoting no monitoring in a given time block). Analogous to the aggregation of sampling frequency variables, groups of vital sign missing data rates have been consolidated into single variables that reflect group averages, as enumerated in Table 5. Consequently, the 24-hour window data comprises observations of Oi = (Wi, Xi, n_Xi, h_m_Xki, while the second

stratified dataset contains observations Oi = (Wi, Xi(b), n_Xi(b), h_m_Xki(b), Yi(b + 1)) for i = 1, ..., n, and b = 1, ..., bi. n_X represents the measurement frequencies, and h_m_X denotes the missing data rates. The distribution and number of each variable type are presented in Table 4.

C.1.2 Association of demographic variables and measurement rates

For each demographic variable of interest A, such as age group, and each measurement pattern outcome variable Y, such as the number of heart rate measurements in the first 24 hours, we defined the observed data as Oi = (Wi, Ai, Yi), i = 1, ..., n, where i indexes the individual and Wi represents adjustment variables including all other demographic factors except A. Our parameter of interest can be described as the marginally adjusted mean outcome: θ(a) = E E[Y |A = a, W ], for each level a of the current demographic variable of interest. For example, theta can be used to describe the adjusted mean of the number of heart rate measures over a 12 hour time period in θ(Hispanic) vs θ(White) adjusted for confounding variables including health status variables such as the Sequential Organ Failure Assessment (SOFA) score and the other demographic variables.

**Table S2: Groupings of strongly correlated count variables.** This table presents grouped variables. Since many measurements and monitoring variables are often recorded together, we grouped those that are commonly co-measured and consolidated their measurement frequencies and missing data rates into average values. Similarly, laboratory tests frequently ordered together were grouped, with their measurement frequencies summarized as group-level averages.

| **Output Variables** | **Strongly Correlated Variables** |  |  |  |
| --- | --- | --- | --- | --- |
| ***Vital sign measurement frequencies (n_VS)*** | | | | |
| **n_BP** | n_BPs | n_BPd |  |  |
| **n_GCS** | n_GCS_Eye | n_GCS_Motor | n_GCS_Verbal |  |
| **n_HR_SpO2** | n_HR | n_SpO2 |  |  |
| ***Vital sign missing data rates (h_m_VS)*** | | | | |
| **h_m_BP** | h_m_BPs | h_m_BPd |  |  |
| **h_m_GCS** | h_m_GCS_Eye | h_m_GCS_Motor | h_m_GCS_Verbal |  |
| **h_m_HR_SpO2** | h_m_HR | h_m_SpO2 |  |  |
| ***Laboratory measurement frequencies (n_LT)*** | | | | |
| **n_lab_grp1** | n_ALT | n_ALK | n_AST | n_TBil |
| **n_lab_grp2** | n_pH | n_PCO2 | n_PO2 | n_BE |
| **n_lab_grp3** | n_Na | n_K | n_Cl | n_HCO3 |
|  | n_AG | n_BG | n_BUN | n_Cr |
| **n_lab_grp4** | n_WBCs | n_RBCs | n_HGB | n_MCV |
|  | n_MCH | n_MCHC | n_RDW | n_PLT |
| **n_lab_grp5** | n_MO | n_EO | n_BA | n_NE |
| **n_lab_grp6** | n_Ca | n_Mg | n_Phos |  |
| **n_lab_grp7** | n_PTT | n_PT |  |  |

**Table S3. List of variable types in the data matrix.** This table lists the variable types, number of variables in each variable type, and example variables in each variable type.

| **Variable Type** | **Count** | **Samples** |  |  |  |
| --- | --- | --- | --- | --- | --- |
| ID | 2 | ICUSTAY_ID | time_block |  |  |
| Outcome(Y) | 1 | Y_b_plus_1 |  |  |  |
| Baseline (W) | 10 | AGE | GENDER_F | ETHNICITY_W | ETHNICITY_B |
| Baseline frequency (n_W) | 7 | n_AGE | n_GENDER | n_ETHNICITY | n_LANGUAGE |
| Vital Sign (VS) | 11 | BPd | BPm | BPs | GCS_Eye |
| Vital sign measurement frequency (n_VS) | 7 | n_BP | n_BPm | n_GCS | n_GCS_Total |
| Vital sign missing data rates (h_m_VS) | 7 | h_m_BP | h_m_BPm | h_m_GCS | h_m_GCS_Total |
| Laboratory testings (LT) | 35 | ALT | ALK | AST | pH |
| Laboratory testing frequencies (n_LT) | 9 | n_HCT | n_Lac | n_lab_grp1 | n_lab_grp2 |

### Appendix D. TMLE estimated rates - complete plots

D.1. Age groups


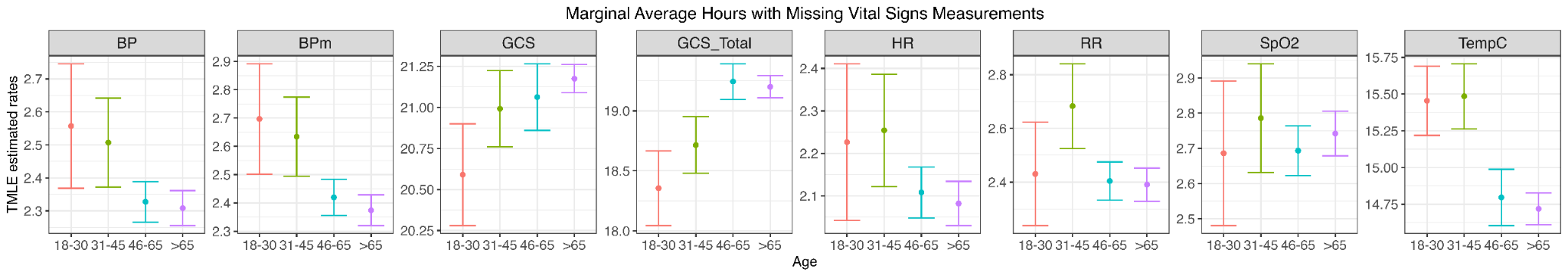

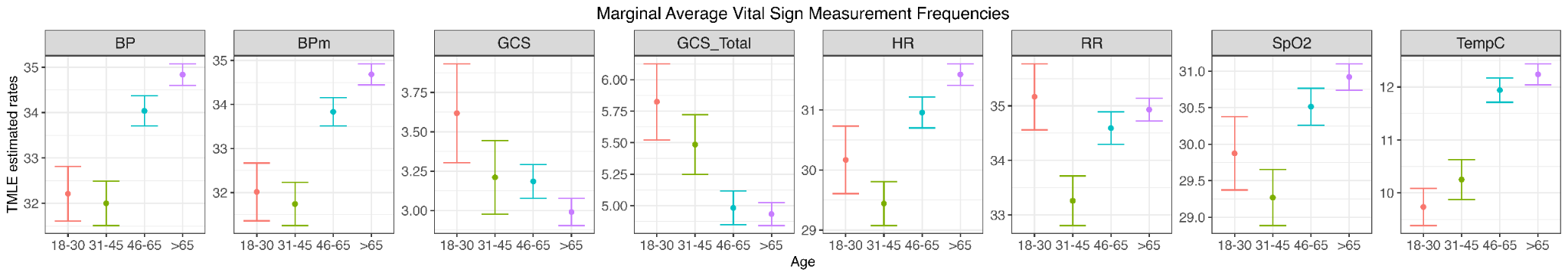

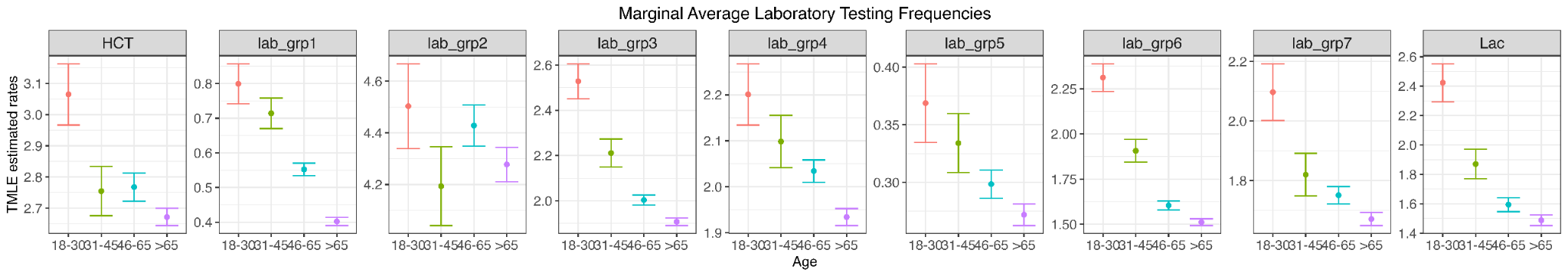


D.2. Gender


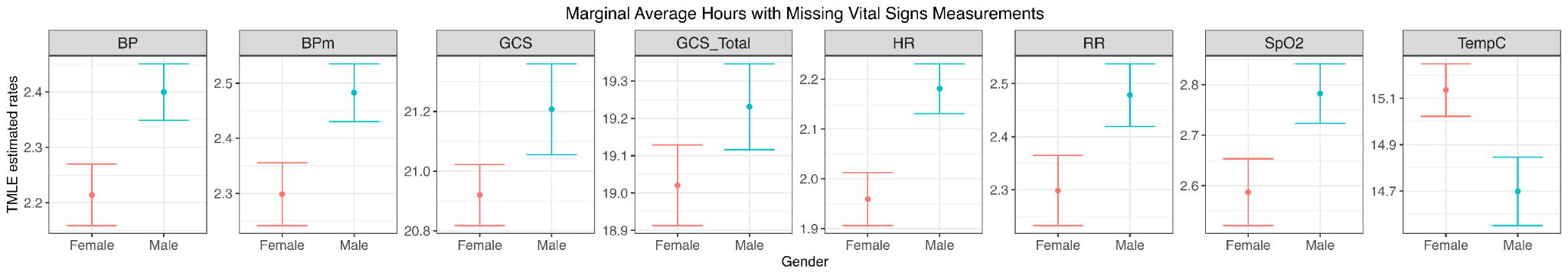

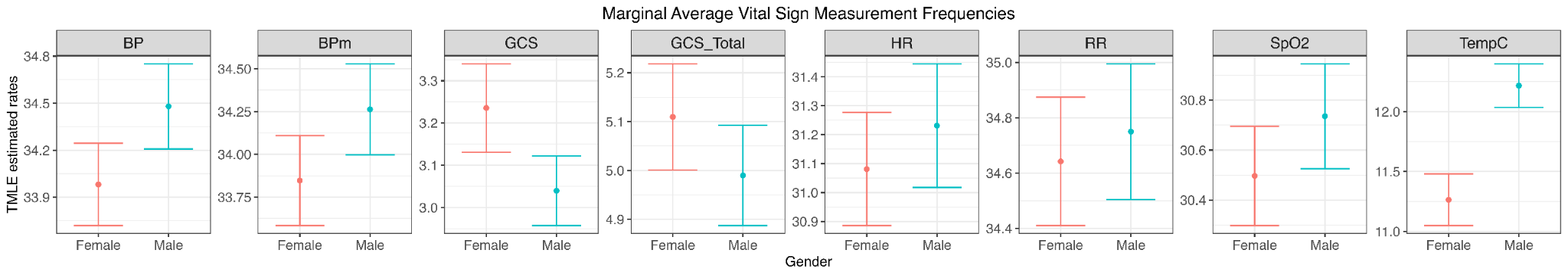

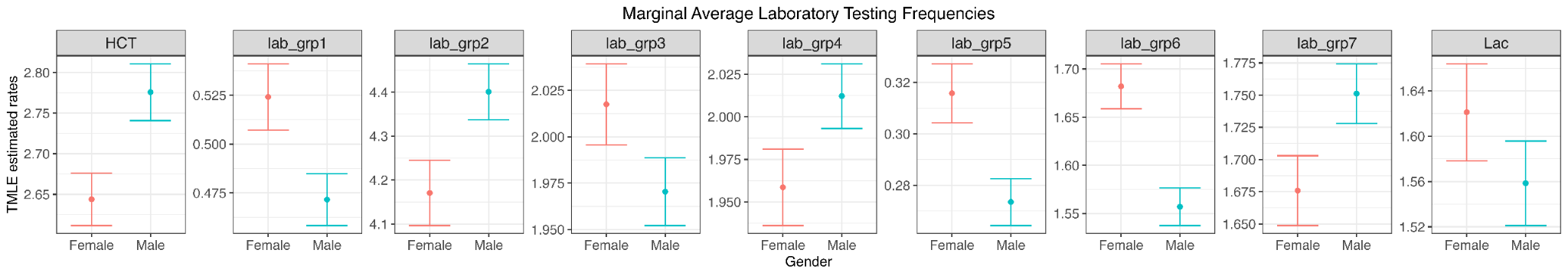


D.3. Ethnicity/Race


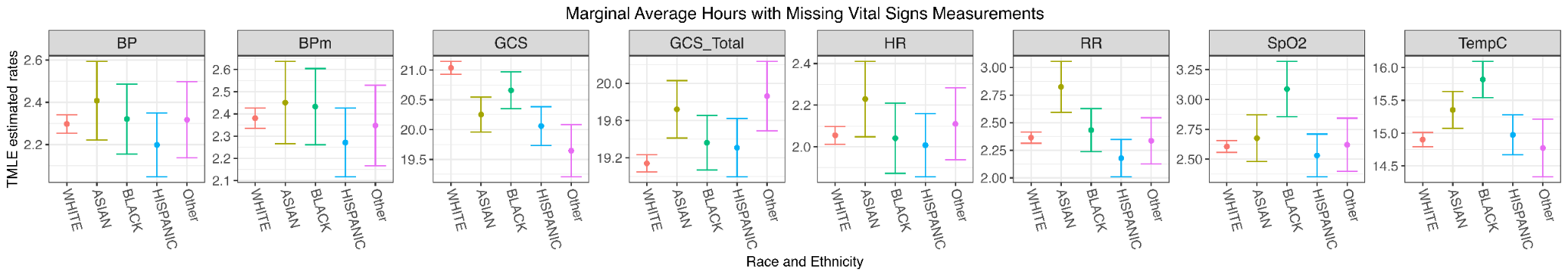

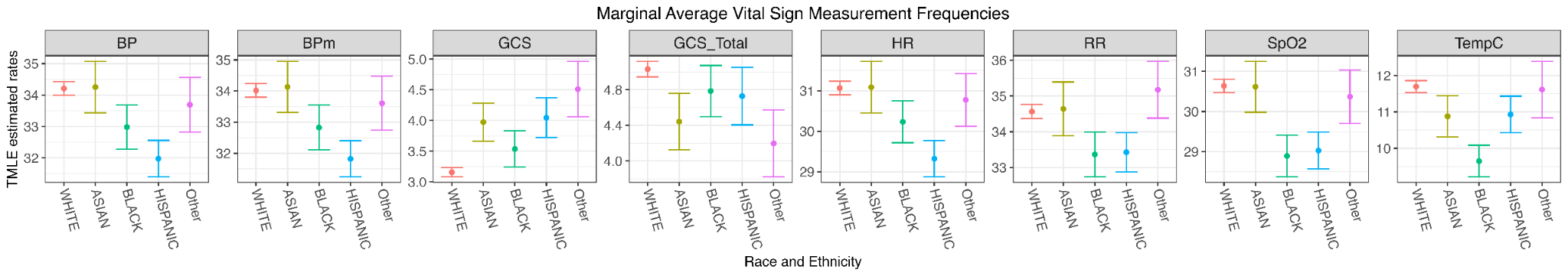

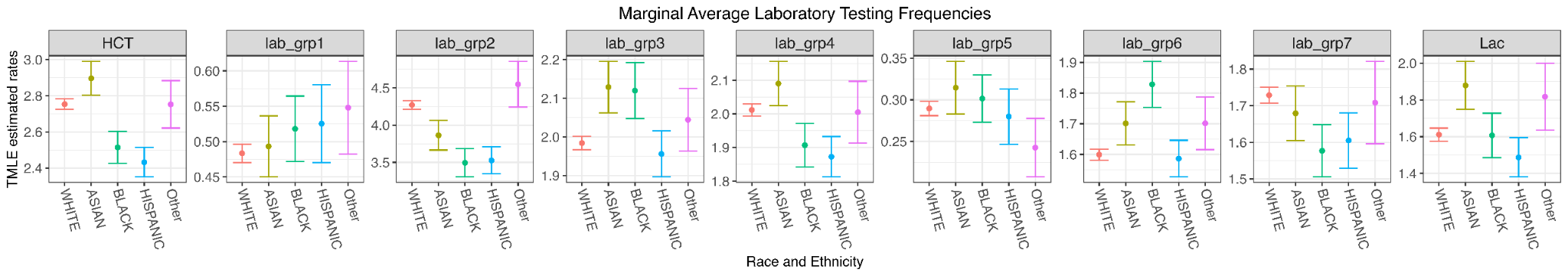


D.4. Insurance


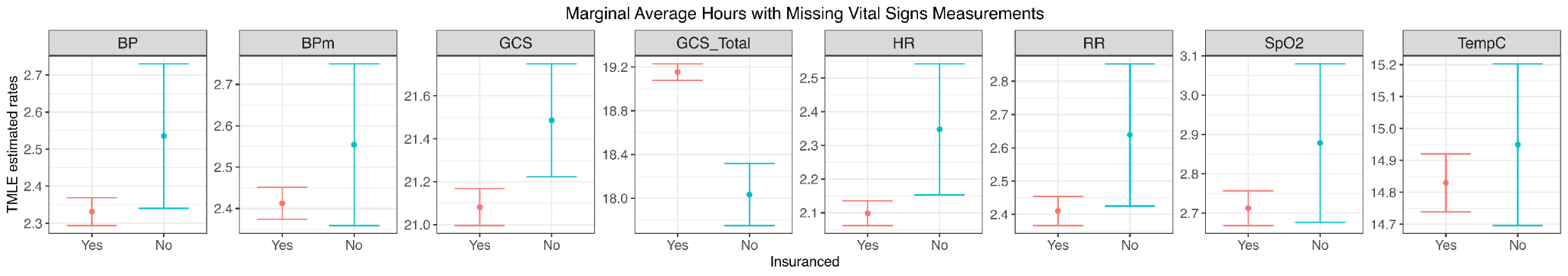

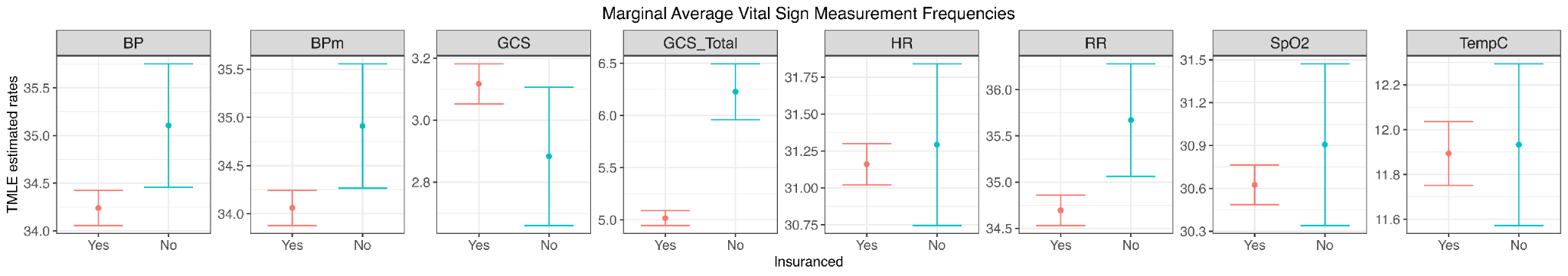

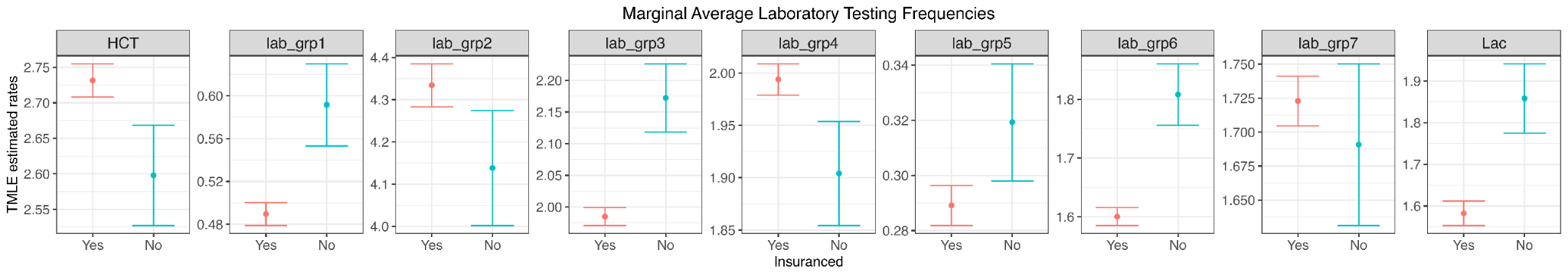


D.5. Language


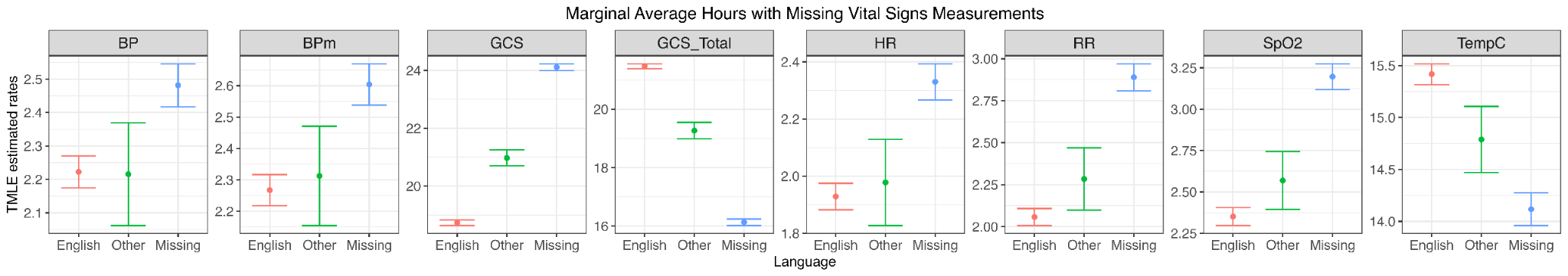

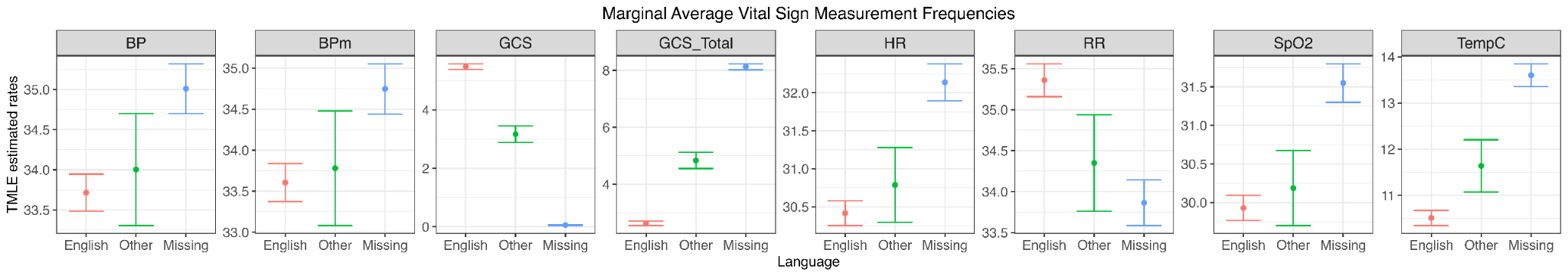

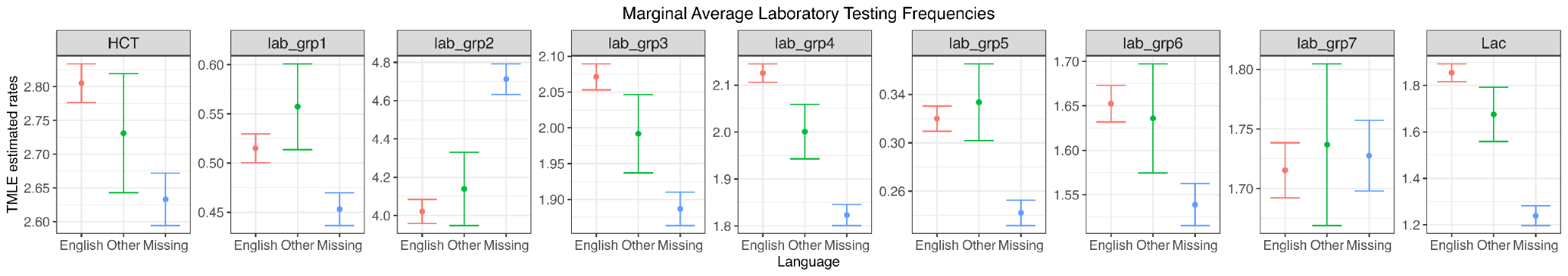


D.6. Marital status


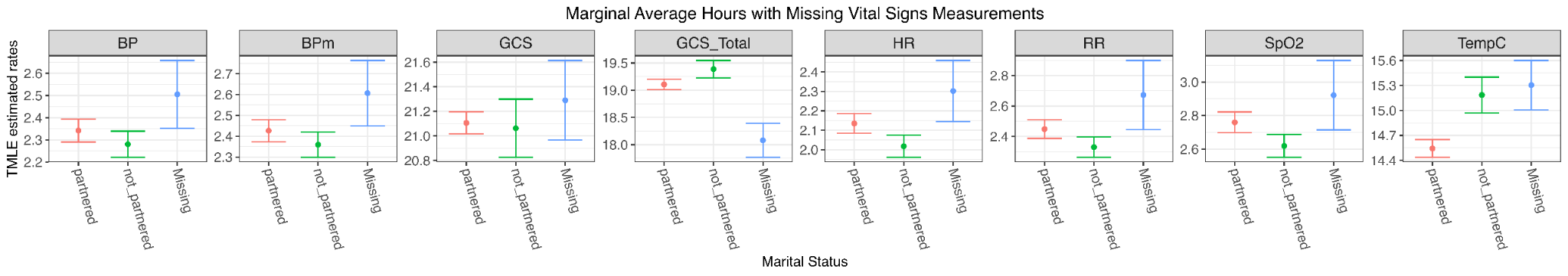

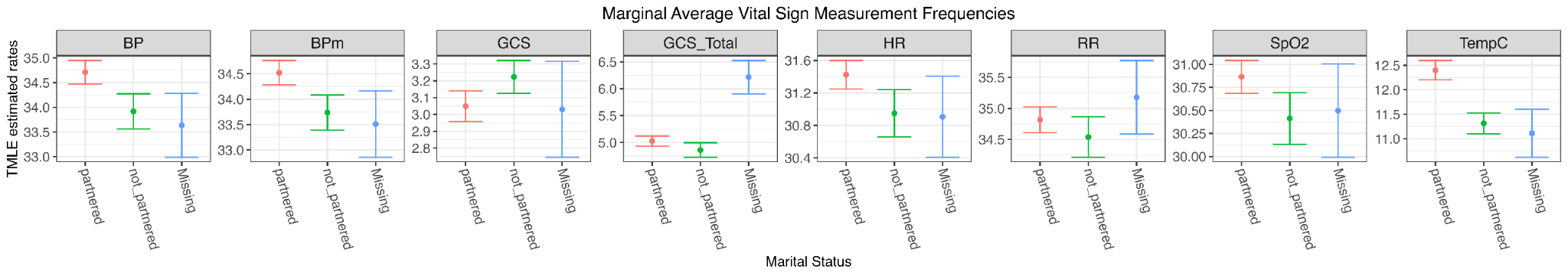

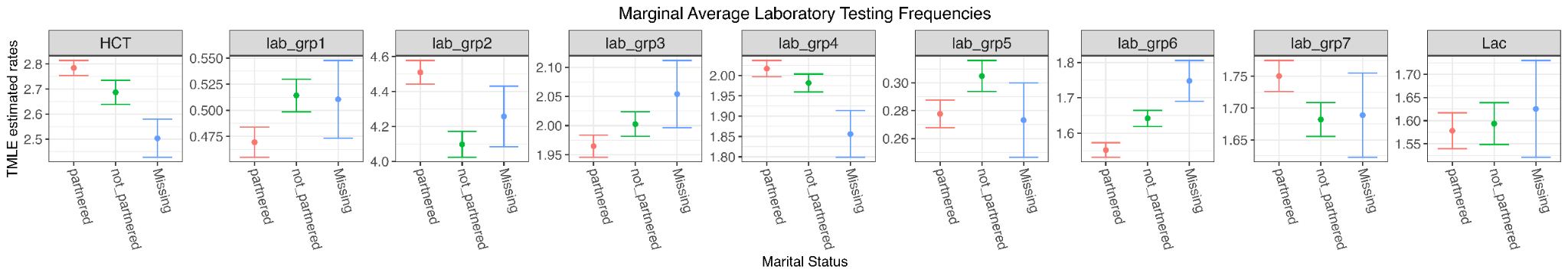


D.7. Religion


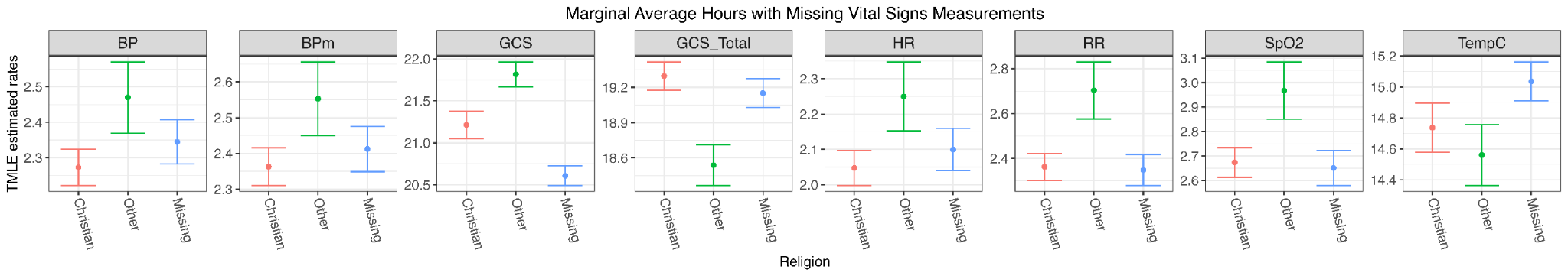

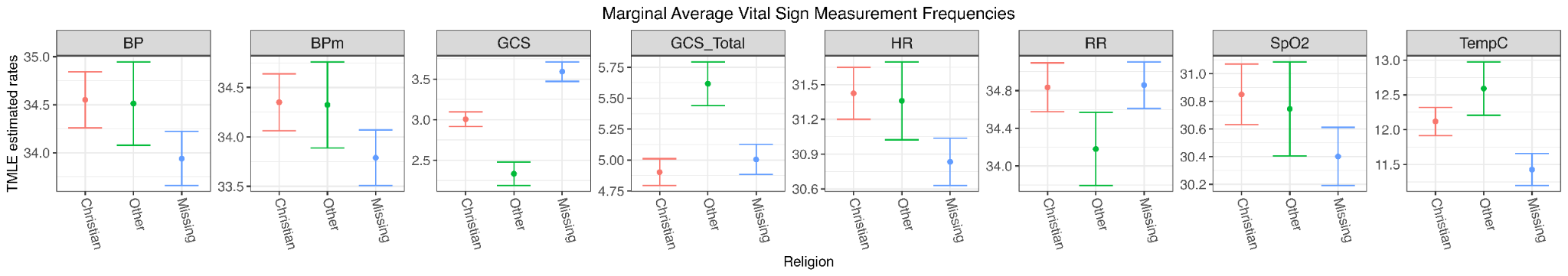

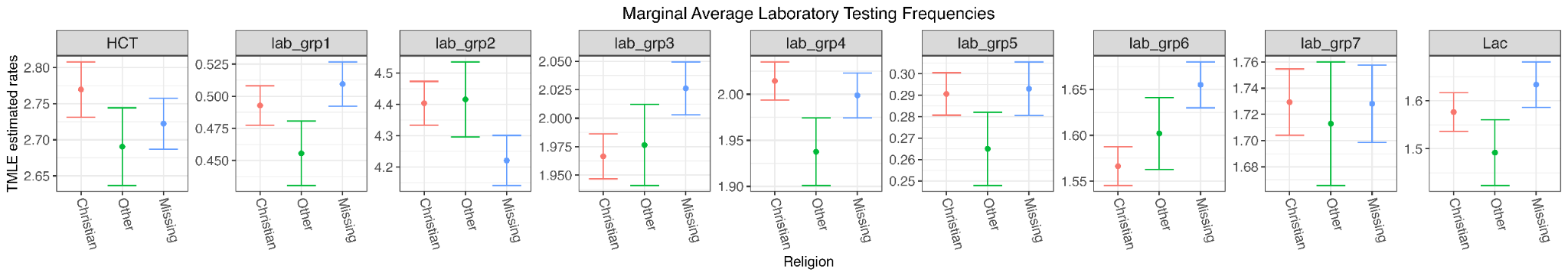


### Appendix E. Performances of SL candidate learners

Within the candidate learners explored in the discrete SuperLearner framework, Random Forest (RF) models demonstrated the best performance, as shown in Table 6. Figure 4 and Table 2 present the fit metrics of three RF models, including 95\% confidence intervals, which were evaluated through the ID-stratified 10-fold cross-validation process. Each RF model was trained using different combinations of predictors, which included the measured values, measurement rates, and missing data variables.

Among the three hhhgy7 models, the one trained on a combination of both counting variables and original value variables achieved the best fit, with an AUC of 0.86 (0.84, 0.87). The performance of this model was closely comparable to that of the model trained solely on original value variables, which had an AUC of 0.85 (0.83, 0.87). The p-value for the comparison of AUCs between these two top-performing models was approximately 0.437, indicating no statistically significant difference in their predictive performance.

In contrast, the model trained exclusively on generated measurement rate and missing data variables exhibited lower performance, with an AUC of 0.76 (0.74, 0.77). The p-values for the comparison of its AUC with those of the other two models were less than 0.001, indicating statistically significant differences. Despite its lower performance, this model still achieved notable predictive accuracy, suggesting that measurement rate variables alone can effectively predict mortality, albeit less effectively than when combined with original value variables.

**Table S4. Prediction average performance metrics across CV of candidate learners.** This table reports the average performance metrics—including negative log-likelihood (NLL), mean squared error (MSE), area under the precision-recall curve (AUCPR), and area under the receiver operating characteristic curve (AUC)—across cross-validation folds for various predictive models. Despite variability in performance, most models achieved comparable AUC values, with Random Forest, Ridge, ElasticNet, and XGBoost showing the strongest overall performance.

|  | **NLL** | **MSE** | **AUCPR** | **AUC** |
| --- | --- | --- | --- | --- |
| GLM | 0.1896 | 0.05073 | 0.1661 | 0.8459 |
| GAM | 0.2028 | 0.05186 | 0.1630 | 0.8175 |
| RF | 0.1716 | 0.04770 | 0.1910 | 0.8536 |
| BayesianGLM | 0.1891 | 0.05068 | 0.1669 | 0.8472 |
| Lassso | 0.1877 | 0.05094 | 0.1649 | 0.8529 |
| Ridge | 0.1873 | 0.05093 | 0.1671 | 0.8538 |
| ElasticNet | 0.1875 | 0.05094 | 0.1656 | 0.8534 |
| XGBoost | 0.2151 | 0.05143 | 0.1917 | 0.8540 |
| BayesianTrees | 0.7048 | 0.25576 | 0.1855 | 0.8722 |

### Appendix F. Relationship Between Measurement Patterns and Length of ICU Stay

To investigate the relationship between measurement patterns and an additional patient outcome, length of stay (LoS), we analyzed the association of measurement frequencies and missing data rates with LoS. Correlation analyses and linear regression models were used, including adjustments for SOFA scores to account for disease severity.

The results indicate that higher measurement frequencies of vital signs and lower missing data rates are associated with shorter LoS, while certain patterns in laboratory tests suggest a complex relationship with LoS. Detailed results for each variable, including correlation coefficients (corr), regression slopes (slope), and adjusted slopes with SOFA scores (slope_w_sofa), are presented in the following table.

**Table S5. Relationships Between Measurement Patterns and Length of ICU Stay Among Patients without ICU Mortality**

This table summarizes the correlation coefficients (Correlation), unadjusted regression slopes (Slope), and regression slopes adjusted for SOFA scores (Slope with SOFA) for the association between various measurement pattern variables (vital sign measurement frequencies, vital sign missing data rates, and laboratory measurement frequencies) and Length of ICU Stay among patients without ICU Mortality. P-values are provided with statistical significance indicated as p < 0.05 (*), < 0.01 (**), and < 0.001 (***).

|  | **Correlation** | **Slope** | **Slope (w/SOFA)** |
| --- | --- | --- | --- |
| Vital Sign Measurement Frequencies | | | |
| n_BP | 0.05 (2e-17***) | 0.75 (2e-17***) | 0.06 (5e-01) |
| n_BPm | 0.06 (1e-18***) | 0.78 (1e-18***) | 0.08 (4e-01) |
| n_GCS | -0.03 (2e-07***) | -1.17 (2e-07***) | -0.83 (2e-04***) |
| n_GCS_Total | 0.06 (5e-24***) | 2.19 (5e-24***) | 2.92 (6e-43***) |
| n_HR | 0.05 (3e-14***) | 0.92 (3e-14***) | -0.05 (7e-01) |
| n_SpO2 | 0.06 (2e-18***) | 1.04 (2e-18***) | 0.19 (1e-01) |
| n_RR | 0.1 (2e-60***) | 1.64 (2e-60***) | 0.7 (2e-11***) |
| n_TempC | -0.03 (9e-05***) | -0.4 (9e-05***) | -0.89 (2e-18***) |
| Vital Sign Missing Data Rates | | | |
| h_m_BP | 0.02 (5e-04***) | 1.43 (5e-04***) | 2.4 (4e-09***) |
| h_m_BPm | 0.02 (2e-03**) | 1.24 (2e-03**) | 2.22 (2e-08***) |
| h_m_GCS | 0.03 (2e-07***) | 1.24 (2e-07***) | 0.9 (1e-04***) |
| h_m_GCS_Total | -0.06 (5e-24***) | -2.23 (5e-24***) | -2.98 (8e-43***) |
| h_m_HR | 0.03 (4e-07***) | 2.17 (4e-07***) | 2.81 (2e-11***) |
| h_m_SpO2 | 0.02 (8e-04***) | 1.18 (8e-04***) | 1.61 (3e-06***) |
| h_m_RR | 0.03 (2e-05***) | 1.5 (2e-05***) | 1.9 (4e-08***) |
| h_m_TempC | 0.02 (2e-02*) | 0.46 (2e-02*) | 1.43 (7e-14***) |
| Lab Test Measurement Frequencies | | | |
| n_HCT | 0.04 (4e-08***) | 3.78 (4e-08***) | -2.18 (2e-03**) |
| n_Lac | 0.2 (2e-207***) | 15.69 (2e-207***) | 10.9 (1e-84***) |
| n_lab_grp1 | 0.14 (3e-109***) | 30.68 (3e-109***) | 18.12 (7e-36***) |
| n_lab_grp2 | 0.11 (6e-64***) | 4.86 (6e-64***) | 1.54 (6e-07***) |
| n_lab_grp3 | 0.14 (2e-110***) | 23.84 (2e-110***) | 14.81 (1e-40***) |
| n_lab_grp4 | 0.09 (2e-41***) | 13.5 (2e-41***) | 3.66 (5e-04***) |
| n_lab_grp5 | 0.1 (2e-54***) | 32.26 (2e-54***) | 21.07 (3e-24***) |
| n_lab_grp6 | 0.18 (3e-174***) | 27.72 (3e-174***) | 21.28 (8e-100***) |
| n_lab_grp7 | 0.13 (6e-91***) | 16.62 (6e-91***) | 9.57 (3e-29***) |

**Table S6. Relationships Between Measurement Patterns and Length of ICU Stay Among Patients with ICU Mortality**

This table summarizes the correlation coefficients (Correlation), unadjusted regression slopes (Slope), and regression slopes adjusted for SOFA scores (Slope with SOFA) for the association between various measurement pattern variables (vital sign measurement frequencies, vital sign missing data rates, and laboratory measurement frequencies) and Length of ICU Stay among patients with ICU Mortality. P-values are provided with statistical significance indicated as p < 0.05 (*), < 0.01 (**), and < 0.001 (***).

|  | **Correlation** | **Slope** | **Slope (w/SOFA)** |
| --- | --- | --- | --- |
| Vital Sign Measurement Frequencies | | | |
| n_BP | -0.39 (1e-08***) | -0.04 (1e-08***) | -0.04 (1e-08***) |
| n_BPm | -0.41 (2e-09***) | -0.04 (2e-09***) | -0.04 (2e-09***) |
| n_GCS | -0.04 (6e-01) | -0.03 (6e-01) | -0.03 (6e-01) |
| n_GCS_Total | -0.24 (6e-04***) | -0.07 (6e-04***) | -0.07 (7e-04***) |
| n_HR | -0.42 (5e-10***) | -0.05 (5e-10***) | -0.05 (6e-10***) |
| n_SpO2 | -0.36 (1e-07***) | -0.04 (1e-07***) | -0.04 (2e-07***) |
| n_RR | -0.41 (1e-09***) | -0.04 (1e-09***) | -0.04 (1e-09***) |
| n_TempC | -0.33 (2e-06***) | -0.13 (2e-06***) | -0.13 (2e-06***) |
| Vital Sign Missing Data Rates | | | |
| h_m_BP | -0.46 (4e-12***) | -0.28 (4e-12***) | -0.28 (5e-12***) |
| h_m_BPm | -0.47 (2e-12***) | -0.28 (2e-12***) | -0.28 (3e-12***) |
| h_m_GCS | -0.33 (1e-06***) | -0.04 (1e-06***) | -0.04 (2e-06***) |
| h_m_GCS_Total | -0.38 (3e-08***) | -0.08 (3e-08***) | -0.08 (3e-08***) |
| h_m_HR | -0.47 (2e-12***) | -0.28 (2e-12***) | -0.28 (3e-12***) |
| h_m_SpO2 | -0.48 (7e-13***) | -0.24 (7e-13***) | -0.24 (5e-13***) |
| h_m_RR | -0.44 (6e-11***) | -0.22 (6e-11***) | -0.22 (7e-11***) |
| h_m_TempC | -0.3 (1e-05***) | -0.05 (1e-05***) | -0.05 (1e-05***) |
| Lab Test Measurement Frequencies | | | |
| n_HCT | -0.27 (1e-04***) | -0.2 (1e-04***) | -0.2 (8e-05***) |
| n_Lac | -0.28 (5e-05***) | -0.26 (5e-05***) | -0.29 (1e-05***) |
| n_lab_grp1 | -0.19 (7e-03**) | -0.53 (7e-03**) | -0.62 (3e-03**) |
| n_lab_grp2 | -0.31 (8e-06***) | -0.25 (8e-06***) | -0.26 (3e-06***) |
| n_lab_grp3 | -0.27 (9e-05***) | -0.46 (9e-05***) | -0.49 (4e-05***) |
| n_lab_grp4 | -0.32 (3e-06***) | -0.62 (3e-06***) | -0.68 (8e-07***) |
| n_lab_grp5 | -0.25 (3e-04***) | -1.02 (3e-04***) | -1.1 (1e-04***) |
| n_lab_grp6 | -0.28 (7e-05***) | -0.48 (7e-05***) | -0.51 (3e-05***) |
| n_lab_grp7 | -0.26 (2e-04***) | -0.41 (2e-04***) | -0.44 (9e-05***) |

### Appendix G. Estimated Average Measurement Frequencies and Missing Data Rates

In the following tables, we present the estimated average measurement frequencies and missing data rates along with their 95% confidence intervals from both the primary and sensitive analyses. For the primary analysis, Targeted Maximum Likelihood Estimation (TMLE) was employed, adjusting for other baseline characteristics and Sequential Organ Failure Assessment (SOFA) scores as confounders. The sensitive analyses employed various methods to assess the robustness of the findings, including (1) linear regression without confounders, (2) linear regression with SOFA scores as a confounder, (3) linear regression with SAPS-II scores as a confounder, (4) TMLE adjusting for other baseline characteristics as confounders, and (5) TMLE adjusting for baseline characteristics and SAPS-II scores as confounders. These sensitivity analyses were conducted to evaluate the impact of different confounding adjustments on the estimation of measurement frequencies.

#### Appendix G.1.1. Estimated Average Measurement Frequencies for Vital Signs across Age Groups

| **Y** | **method** | **18-30** | **31-45** | **46-65** | **>65** |
| --- | --- | --- | --- | --- | --- |
| n_BP | [prm]TMLE-dmg_sofa | 32.211 (31.61, 32.81) | 32.002 (31.51, 32.49) | 34.038 (33.71, 34.37) | 34.837 (34.6, 35.08) |
|  | GLM | 31.038 (30.17, 31.9) | 31.26 (30.21, 32.31) | 33.734 (32.82, 34.65) | 34.909 (34.01, 35.81) |
|  | GLM-sofa | 27.443 (26.57, 28.31) | 27.133 (26.11, 28.16) | 29.121 (28.22, 30.02) | 30.007 (29.12, 30.89) |
|  | GLM-saps | 26.5 (25.59, 27.41) | 25.794 (24.76, 26.83) | 27.027 (26.11, 27.94) | 26.489 (25.57, 27.41) |
|  | TMLE-dmg | 31.157 (30.55, 31.77) | 31.469 (30.99, 31.95) | 33.942 (33.62, 34.27) | 35.125 (34.88, 35.37) |
|  | TMLE-dmg_saps | 35.413 (34.81, 36.02) | 33.6 (32.99, 34.21) | 34.557 (34.21, 34.9) | 34.005 (33.78, 34.24) |
| n_BPm | [prm]TMLE-dmg_sofa | 32.015 (31.36, 32.67) | 31.74 (31.25, 32.23) | 33.834 (33.51, 34.16) | 34.684 (34.44, 34.93) |
|  | GLM | 30.831 (29.97, 31.69) | 30.992 (29.95, 32.03) | 33.524 (32.61, 34.44) | 34.758 (33.86, 35.65) |
|  | GLM-sofa | 27.143 (26.28, 28.01) | 26.758 (25.74, 27.77) | 28.791 (27.9, 29.68) | 29.729 (28.85, 30.6) |
|  | GLM-saps | 26.151 (25.25, 27.05) | 25.355 (24.33, 26.38) | 26.608 (25.7, 27.51) | 26.075 (25.16, 26.99) |
|  | TMLE-dmg | 30.931 (30.26, 31.6) | 31.191 (30.71, 31.68) | 33.738 (33.42, 34.06) | 34.976 (34.73, 35.22) |
|  | TMLE-dmg_saps | 35.337 (34.72, 35.96) | 33.37 (32.76, 33.98) | 34.372 (34.03, 34.71) | 33.825 (33.59, 34.06) |
| n_GCS | [prm]TMLE-dmg_sofa | 3.618 (3.3, 3.93) | 3.211 (2.98, 3.44) | 3.185 (3.08, 3.29) | 2.99 (2.9, 3.08) |
|  | GLM | 3.937 (3.63, 4.25) | 3.356 (2.98, 3.73) | 3.214 (2.89, 3.54) | 2.911 (2.59, 3.23) |
|  | GLM-sofa | 4.204 (3.89, 4.52) | 3.662 (3.29, 4.04) | 3.557 (3.23, 3.89) | 3.275 (2.95, 3.6) |
|  | GLM-saps | 3.939 (3.61, 4.27) | 3.358 (2.98, 3.73) | 3.217 (2.88, 3.55) | 2.915 (2.58, 3.25) |
|  | TMLE-dmg | 3.69 (3.36, 4.02) | 3.273 (3.03, 3.51) | 3.2 (3.09, 3.31) | 2.972 (2.89, 3.06) |
|  | TMLE-dmg_saps | 3.899 (3.65, 4.15) | 3.255 (3.01, 3.5) | 3.204 (3.1, 3.31) | 3.029 (2.94, 3.12) |
| n_GCS_Total | [prm]TMLE-dmg_sofa | 5.824 (5.52, 6.13) | 5.486 (5.25, 5.72) | 4.982 (4.85, 5.12) | 4.934 (4.84, 5.03) |
|  | GLM | 5.709 (5.37, 6.05) | 5.553 (5.14, 5.97) | 4.949 (4.59, 5.31) | 4.884 (4.53, 5.24) |
|  | GLM-sofa | 6.367 (6.02, 6.72) | 6.31 (5.9, 6.72) | 5.794 (5.43, 6.16) | 5.782 (5.43, 6.14) |
|  | GLM-saps | 6.359 (6, 6.72) | 6.338 (5.92, 6.75) | 5.911 (5.54, 6.28) | 6.091 (5.72, 6.46) |
|  | TMLE-dmg | 6.147 (5.82, 6.48) | 5.617 (5.37, 5.86) | 4.993 (4.86, 5.13) | 4.878 (4.79, 4.97) |
|  | TMLE-dmg_saps | 5.191 (4.96, 5.42) | 5.249 (5.01, 5.49) | 4.904 (4.77, 5.04) | 4.949 (4.86, 5.04) |
| n_HR | [prm]TMLE-dmg_sofa | 30.168 (29.61, 30.73) | 29.441 (29.07, 29.81) | 30.957 (30.7, 31.21) | 31.592 (31.41, 31.77) |
|  | GLM | 28.903 (28.25, 29.56) | 28.853 (28.06, 29.65) | 30.685 (29.99, 31.38) | 31.636 (30.95, 32.32) |
|  | GLM-sofa | 26.25 (25.59, 26.91) | 25.807 (25.03, 26.58) | 27.28 (26.6, 27.96) | 28.018 (27.35, 28.69) |
|  | GLM-saps | 25.672 (24.98, 26.36) | 24.96 (24.18, 25.74) | 25.909 (25.22, 26.6) | 25.639 (24.94, 26.34) |
|  | TMLE-dmg | 29.267 (28.7, 29.84) | 29.059 (28.7, 29.42) | 30.887 (30.63, 31.14) | 31.825 (31.64, 32.01) |
|  | TMLE-dmg_saps | 32.448 (31.95, 32.94) | 30.445 (29.99, 30.9) | 31.301 (31.03, 31.57) | 30.969 (30.8, 31.14) |
| n_SpO2 | [prm]TMLE-dmg_sofa | 29.874 (29.37, 30.38) | 29.269 (28.89, 29.65) | 30.514 (30.26, 30.77) | 30.92 (30.74, 31.1) |
|  | GLM | 28.749 (28.09, 29.41) | 28.703 (27.9, 29.5) | 30.278 (29.58, 30.98) | 31.031 (30.34, 31.72) |
|  | GLM-sofa | 26.303 (25.64, 26.97) | 25.894 (25.11, 26.68) | 27.137 (26.45, 27.82) | 27.694 (27.02, 28.37) |
|  | GLM-saps | 25.93 (25.24, 26.62) | 25.307 (24.52, 26.1) | 26.11 (25.41, 26.81) | 25.799 (25.09, 26.51) |
|  | TMLE-dmg | 29.021 (28.51, 29.53) | 28.848 (28.47, 29.23) | 30.431 (30.18, 30.68) | 31.165 (30.98, 31.35) |
|  | TMLE-dmg_saps | 31.98 (31.5, 32.46) | 30.189 (29.72, 30.66) | 30.831 (30.57, 31.09) | 30.356 (30.18, 30.53) |
| n_RR | [prm]TMLE-dmg_sofa | 35.166 (34.56, 35.77) | 33.261 (32.81, 33.72) | 34.592 (34.3, 34.89) | 34.932 (34.72, 35.14) |
|  | GLM | 33.426 (32.65, 34.2) | 32.463 (31.52, 33.4) | 34.291 (33.47, 35.11) | 34.991 (34.18, 35.8) |
|  | GLM-sofa | 29.233 (28.47, 30) | 27.649 (26.75, 28.55) | 28.909 (28.12, 29.7) | 29.273 (28.5, 30.05) |
|  | GLM-saps | 27.666 (26.87, 28.46) | 25.524 (24.62, 26.43) | 25.777 (24.97, 26.58) | 24.302 (23.49, 25.12) |
|  | TMLE-dmg | 33.675 (33.03, 34.32) | 32.639 (32.18, 33.1) | 34.493 (34.2, 34.79) | 35.263 (35.04, 35.48) |
|  | TMLE-dmg_saps | 39.408 (38.87, 39.95) | 35.32 (34.76, 35.88) | 35.276 (34.97, 35.59) | 33.806 (33.6, 34.01) |
| n_TempC | [prm]TMLE-dmg_sofa | 9.735 (9.38, 10.09) | 10.251 (9.88, 10.63) | 11.939 (11.71, 12.17) | 12.238 (12.04, 12.44) |
|  | GLM | 8.933 (8.26, 9.61) | 9.629 (8.81, 10.45) | 11.836 (11.12, 12.55) | 12.537 (11.83, 13.24) |
|  | GLM-sofa | 7.157 (6.47, 7.85) | 7.59 (6.78, 8.4) | 9.556 (8.84, 10.27) | 10.115 (9.41, 10.82) |
|  | GLM-saps | 8.88 (8.16, 9.6) | 9.564 (8.74, 10.39) | 11.757 (11.03, 12.48) | 12.437 (11.7, 13.17) |
|  | TMLE-dmg | 9.163 (8.8, 9.53) | 9.879 (9.52, 10.23) | 11.798 (11.57, 12.03) | 12.501 (12.29, 12.71) |
|  | TMLE-dmg_saps | 10.365 (10.03, 10.7) | 10.196 (9.83, 10.56) | 11.792 (11.57, 12.02) | 12.267 (12.07, 12.47) |

#### Appendix G.1.2. Estimated Average Missing Data Rates for Vital Signs across Age Groups

| **Y** | **method** | **18-30** | **31-45** | **46-65** | **>65** |
| --- | --- | --- | --- | --- | --- |
| h_m_BP | [prm]TMLE-dmg_sofa | 2.557 (2.37, 2.75) | 2.507 (2.37, 2.64) | 2.328 (2.27, 2.39) | 2.308 (2.26, 2.36) |
|  | GLM | 2.522 (2.34, 2.7) | 2.445 (2.23, 2.66) | 2.305 (2.12, 2.49) | 2.233 (2.05, 2.42) |
|  | GLM-sofa | 2.753 (2.57, 2.94) | 2.711 (2.5, 2.93) | 2.602 (2.41, 2.79) | 2.549 (2.36, 2.73) |
|  | GLM-saps | 2.995 (2.81, 3.18) | 3.015 (2.8, 3.23) | 3.003 (2.81, 3.19) | 3.111 (2.92, 3.3) |
|  | TMLE-dmg | 2.642 (2.45, 2.83) | 2.561 (2.42, 2.7) | 2.34 (2.28, 2.4) | 2.275 (2.22, 2.33) |
|  | TMLE-dmg_saps | 2.261 (2.13, 2.39) | 2.299 (2.17, 2.43) | 2.279 (2.22, 2.34) | 2.429 (2.37, 2.49) |
| h_m_BPm | [prm]TMLE-dmg_sofa | 2.697 (2.5, 2.89) | 2.634 (2.49, 2.77) | 2.42 (2.36, 2.48) | 2.374 (2.32, 2.43) |
|  | GLM | 2.634 (2.45, 2.82) | 2.566 (2.35, 2.79) | 2.399 (2.21, 2.59) | 2.297 (2.11, 2.49) |
|  | GLM-sofa | 2.884 (2.7, 3.07) | 2.853 (2.63, 3.07) | 2.72 (2.53, 2.91) | 2.638 (2.45, 2.83) |
|  | GLM-saps | 3.131 (2.94, 3.32) | 3.164 (2.94, 3.38) | 3.133 (2.94, 3.33) | 3.219 (3.02, 3.42) |
|  | TMLE-dmg | 2.784 (2.59, 2.98) | 2.693 (2.55, 2.83) | 2.433 (2.37, 2.5) | 2.34 (2.29, 2.39) |
|  | TMLE-dmg_saps | 2.342 (2.21, 2.47) | 2.411 (2.28, 2.55) | 2.371 (2.31, 2.43) | 2.503 (2.44, 2.56) |
| h_m_GCS | [prm]TMLE-dmg_sofa | 20.59 (20.28, 20.9) | 20.992 (20.76, 21.22) | 21.063 (20.86, 21.27) | 21.175 (21.09, 21.26) |
|  | GLM | 20.172 (19.77, 20.58) | 20.754 (20.26, 21.24) | 20.915 (20.49, 21.34) | 21.128 (20.71, 21.55) |
|  | GLM-sofa | 19.98 (19.56, 20.4) | 20.534 (20.04, 21.02) | 20.669 (20.24, 21.1) | 20.866 (20.44, 21.29) |
|  | GLM-saps | 20.199 (19.77, 20.63) | 20.787 (20.3, 21.28) | 20.956 (20.52, 21.39) | 21.179 (20.74, 21.62) |
|  | TMLE-dmg | 20.533 (20.2, 20.86) | 20.941 (20.7, 21.18) | 21.043 (20.85, 21.24) | 21.19 (21.11, 21.27) |
|  | TMLE-dmg_saps | 20.28 (20.03, 20.53) | 20.96 (20.72, 21.21) | 21.05 (20.84, 21.26) | 21.169 (21.08, 21.26) |
| h_m_GCS_Total | [prm]TMLE-dmg_sofa | 18.355 (18.04, 18.67) | 18.715 (18.48, 18.95) | 19.244 (19.1, 19.39) | 19.201 (19.11, 19.29) |
|  | GLM | 18.328 (17.97, 18.69) | 18.54 (18.11, 18.97) | 19.157 (18.78, 19.54) | 19.129 (18.76, 19.5) |
|  | GLM-sofa | 17.73 (17.36, 18.1) | 17.854 (17.42, 18.28) | 18.39 (18.01, 18.77) | 18.313 (17.94, 18.68) |
|  | GLM-saps | 17.697 (17.32, 18.08) | 17.78 (17.35, 18.21) | 18.225 (17.84, 18.61) | 17.958 (17.57, 18.34) |
|  | TMLE-dmg | 18.077 (17.74, 18.42) | 18.596 (18.35, 18.84) | 19.228 (19.08, 19.37) | 19.253 (19.16, 19.34) |
|  | TMLE-dmg_saps | 18.977 (18.75, 19.21) | 18.953 (18.71, 19.19) | 19.327 (19.18, 19.48) | 19.175 (19.08, 19.27) |
| h_m_HR | [prm]TMLE-dmg_sofa | 2.227 (2.04, 2.41) | 2.254 (2.12, 2.39) | 2.108 (2.05, 2.17) | 2.082 (2.03, 2.13) |
|  | GLM | 2.174 (2, 2.35) | 2.169 (1.96, 2.38) | 2.086 (1.9, 2.27) | 2.014 (1.83, 2.19) |
|  | GLM-sofa | 2.324 (2.15, 2.5) | 2.341 (2.13, 2.55) | 2.278 (2.09, 2.46) | 2.218 (2.04, 2.4) |
|  | GLM-saps | 2.563 (2.38, 2.75) | 2.637 (2.43, 2.85) | 2.661 (2.48, 2.85) | 2.736 (2.55, 2.92) |
|  | TMLE-dmg | 2.262 (2.08, 2.45) | 2.294 (2.16, 2.43) | 2.119 (2.06, 2.18) | 2.056 (2.01, 2.11) |
|  | TMLE-dmg_saps | 2.034 (1.91, 2.16) | 2.099 (1.97, 2.23) | 2.072 (2.01, 2.13) | 2.194 (2.13, 2.25) |
| h_m_SpO2 | [prm]TMLE-dmg_sofa | 2.686 (2.48, 2.89) | 2.786 (2.63, 2.94) | 2.693 (2.62, 2.76) | 2.742 (2.68, 2.81) |
|  | GLM | 2.655 (2.45, 2.86) | 2.692 (2.44, 2.94) | 2.667 (2.45, 2.89) | 2.646 (2.43, 2.86) |
|  | GLM-sofa | 2.786 (2.57, 3) | 2.842 (2.59, 3.09) | 2.835 (2.61, 3.06) | 2.824 (2.61, 3.04) |
|  | GLM-saps | 2.941 (2.72, 3.16) | 3.036 (2.78, 3.29) | 3.089 (2.87, 3.31) | 3.176 (2.95, 3.4) |
|  | TMLE-dmg | 2.743 (2.53, 2.95) | 2.84 (2.68, 3) | 2.712 (2.64, 2.78) | 2.707 (2.65, 2.77) |
|  | TMLE-dmg_saps | 2.506 (2.36, 2.65) | 2.647 (2.49, 2.8) | 2.657 (2.59, 2.73) | 2.832 (2.76, 2.9) |
| h_m_RR | [prm]TMLE-dmg_sofa | 2.43 (2.24, 2.62) | 2.683 (2.53, 2.84) | 2.404 (2.33, 2.48) | 2.391 (2.33, 2.45) |
|  | GLM | 2.423 (2.21, 2.63) | 2.587 (2.33, 2.84) | 2.37 (2.15, 2.59) | 2.321 (2.1, 2.54) |
|  | GLM-sofa | 2.564 (2.35, 2.78) | 2.748 (2.5, 3) | 2.55 (2.33, 2.77) | 2.512 (2.29, 2.73) |
|  | GLM-saps | 2.757 (2.54, 2.98) | 2.989 (2.74, 3.24) | 2.863 (2.64, 3.09) | 2.94 (2.71, 3.17) |
|  | TMLE-dmg | 2.512 (2.31, 2.71) | 2.697 (2.54, 2.85) | 2.415 (2.34, 2.49) | 2.361 (2.3, 2.42) |
|  | TMLE-dmg_saps | 2.184 (2.05, 2.31) | 2.509 (2.35, 2.67) | 2.386 (2.31, 2.46) | 2.49 (2.42, 2.56) |
| h_m_TempC | [prm]TMLE-dmg_sofa | 15.455 (15.22, 15.69) | 15.485 (15.26, 15.71) | 14.796 (14.6, 14.99) | 14.719 (14.61, 14.83) |
|  | GLM | 15.881 (15.45, 16.31) | 15.724 (15.21, 16.24) | 14.724 (14.27, 15.18) | 14.476 (14.03, 14.92) |
|  | GLM-sofa | 16.932 (16.5, 17.37) | 16.931 (16.42, 17.45) | 16.074 (15.62, 16.52) | 15.91 (15.47, 16.35) |
|  | GLM-saps | 16.076 (15.62, 16.53) | 15.959 (15.44, 16.48) | 15.013 (14.55, 15.47) | 14.838 (14.37, 15.3) |
|  | TMLE-dmg | 15.857 (15.62, 16.1) | 15.709 (15.5, 15.92) | 14.855 (14.67, 15.04) | 14.587 (14.48, 14.7) |
|  | TMLE-dmg_saps | 15.046 (14.84, 15.26) | 15.43 (15.2, 15.66) | 14.836 (14.64, 15.03) | 14.683 (14.57, 14.79) |

#### Appendix G.1.3. Estimated Average Measurement Frequencies for Lab Tests across Age Groups

| **Y** | **method** | **18-30** | **31-45** | **46-65** | **>65** |
| --- | --- | --- | --- | --- | --- |
| n_HCT | [prm]TMLE-dmg_sofa | 3.065 (2.97, 3.16) | 2.755 (2.68, 2.83) | 2.768 (2.72, 2.81) | 2.672 (2.64, 2.7) |
| n_HCT | GLM | 2.856 (2.74, 2.97) | 2.645 (2.51, 2.78) | 2.75 (2.63, 2.87) | 2.697 (2.58, 2.81) |
|  | GLM-sofa | 2.331 (2.22, 2.44) | 2.042 (1.91, 2.17) | 2.076 (1.96, 2.19) | 1.982 (1.87, 2.1) |
|  | GLM-saps | 2.405 (2.29, 2.52) | 2.101 (1.97, 2.24) | 2.082 (1.96, 2.2) | 1.859 (1.74, 1.98) |
|  | TMLE-dmg | 2.883 (2.78, 2.99) | 2.675 (2.6, 2.75) | 2.751 (2.71, 2.8) | 2.711 (2.68, 2.74) |
|  | TMLE-dmg_saps | 3.422 (3.33, 3.52) | 2.899 (2.8, 2.99) | 2.826 (2.78, 2.87) | 2.609 (2.58, 2.64) |
| n_Lac | [prm]TMLE-dmg_sofa | 2.424 (2.29, 2.55) | 1.87 (1.77, 1.97) | 1.594 (1.55, 1.64) | 1.487 (1.45, 1.52) |
|  | GLM | 1.946 (1.81, 2.09) | 1.696 (1.53, 1.86) | 1.551 (1.4, 1.7) | 1.505 (1.36, 1.65) |
|  | GLM-sofa | 0.819 (0.69, 0.95) | 0.401 (0.25, 0.55) | 0.104 (-0.03, 0.24) | -0.032 (-0.16, 0.1) |
|  | GLM-saps | 0.538 (0.4, 0.68) | -0.001 (-0.16, 0.16) | -0.531 (-0.67, -0.39) | -1.108 (-1.25, -0.97) |
|  | TMLE-dmg | 1.987 (1.84, 2.13) | 1.718 (1.61, 1.82) | 1.578 (1.53, 1.63) | 1.546 (1.51, 1.58) |
|  | TMLE-dmg_saps | 3.453 (3.31, 3.6) | 2.438 (2.3, 2.58) | 1.791 (1.74, 1.84) | 1.281 (1.25, 1.31) |
| n_lab_grp1 | [prm]TMLE-dmg_sofa | 0.799 (0.74, 0.86) | 0.714 (0.67, 0.76) | 0.552 (0.53, 0.57) | 0.402 (0.39, 0.41) |
|  | GLM | 0.674 (0.62, 0.73) | 0.652 (0.59, 0.71) | 0.541 (0.49, 0.59) | 0.398 (0.34, 0.45) |
|  | GLM-sofa | 0.33 (0.28, 0.38) | 0.257 (0.2, 0.31) | 0.1 (0.05, 0.15) | -0.071 (-0.12, -0.02) |
|  | GLM-saps | 0.266 (0.21, 0.32) | 0.16 (0.1, 0.22) | -0.063 (-0.12, -0.01) | -0.36 (-0.41, -0.31) |
|  | TMLE-dmg | 0.654 (0.59, 0.72) | 0.656 (0.61, 0.7) | 0.549 (0.53, 0.57) | 0.411 (0.4, 0.42) |
|  | TMLE-dmg_saps | 1.082 (1.03, 1.14) | 0.918 (0.86, 0.98) | 0.622 (0.6, 0.64) | 0.354 (0.34, 0.36) |
| n_lab_grp2 | [prm]TMLE-dmg_sofa | 4.503 (4.34, 4.67) | 4.194 (4.04, 4.35) | 4.428 (4.35, 4.51) | 4.278 (4.21, 4.34) |
|  | GLM | 3.736 (3.49, 3.98) | 3.788 (3.49, 4.08) | 4.356 (4.1, 4.61) | 4.407 (4.15, 4.66) |
|  | GLM-sofa | 1.928 (1.7, 2.16) | 1.712 (1.44, 1.98) | 2.036 (1.8, 2.27) | 1.941 (1.71, 2.17) |
|  | GLM-saps | 2.134 (1.88, 2.39) | 1.858 (1.57, 2.15) | 1.988 (1.73, 2.24) | 1.434 (1.18, 1.69) |
|  | TMLE-dmg | 3.775 (3.59, 3.96) | 3.894 (3.74, 4.05) | 4.363 (4.28, 4.45) | 4.426 (4.35, 4.5) |
|  | TMLE-dmg_saps | 5.831 (5.65, 6.01) | 4.772 (4.59, 4.96) | 4.602 (4.52, 4.69) | 4.037 (3.97, 4.1) |
| n_lab_grp3 | [prm]TMLE-dmg_sofa | 2.529 (2.45, 2.61) | 2.211 (2.15, 2.27) | 2.003 (1.98, 2.03) | 1.908 (1.89, 1.92) |
|  | GLM | 2.344 (2.28, 2.41) | 2.142 (2.06, 2.22) | 1.985 (1.91, 2.06) | 1.906 (1.84, 1.98) |
|  | GLM-sofa | 1.933 (1.87, 2) | 1.67 (1.59, 1.75) | 1.458 (1.39, 1.53) | 1.346 (1.28, 1.41) |
|  | GLM-saps | 1.733 (1.67, 1.8) | 1.406 (1.33, 1.48) | 1.082 (1.01, 1.15) | 0.773 (0.7, 0.84) |
|  | TMLE-dmg | 2.333 (2.25, 2.42) | 2.142 (2.08, 2.2) | 1.998 (1.97, 2.02) | 1.928 (1.91, 1.95) |
|  | TMLE-dmg_saps | 3.068 (2.99, 3.15) | 2.503 (2.42, 2.58) | 2.092 (2.07, 2.12) | 1.814 (1.8, 1.83) |
| n_lab_grp4 | [prm]TMLE-dmg_sofa | 2.201 (2.13, 2.27) | 2.099 (2.04, 2.16) | 2.034 (2.01, 2.06) | 1.934 (1.92, 1.95) |
|  | GLM | 2.048 (1.98, 2.12) | 2.009 (1.92, 2.1) | 2.023 (1.95, 2.1) | 1.947 (1.87, 2.02) |
|  | GLM-sofa | 1.603 (1.53, 1.67) | 1.499 (1.42, 1.58) | 1.452 (1.38, 1.52) | 1.34 (1.27, 1.41) |
|  | GLM-saps | 1.595 (1.52, 1.67) | 1.464 (1.38, 1.55) | 1.355 (1.28, 1.43) | 1.108 (1.03, 1.18) |
|  | TMLE-dmg | 2.01 (1.94, 2.08) | 2.028 (1.97, 2.08) | 2.025 (2, 2.05) | 1.961 (1.94, 1.98) |
|  | TMLE-dmg_saps | 2.647 (2.57, 2.72) | 2.28 (2.2, 2.36) | 2.097 (2.07, 2.12) | 1.869 (1.85, 1.89) |
| n_lab_grp5 | [prm]TMLE-dmg_sofa | 0.369 (0.33, 0.4) | 0.334 (0.31, 0.36) | 0.298 (0.29, 0.31) | 0.272 (0.26, 0.28) |
|  | GLM | 0.341 (0.31, 0.38) | 0.319 (0.28, 0.36) | 0.287 (0.25, 0.32) | 0.271 (0.23, 0.31) |
|  | GLM-sofa | 0.217 (0.18, 0.25) | 0.177 (0.14, 0.22) | 0.128 (0.09, 0.16) | 0.102 (0.07, 0.14) |
|  | GLM-saps | 0.144 (0.11, 0.18) | 0.082 (0.04, 0.12) | -0.003 (-0.04, 0.03) | -0.094 (-0.13, -0.06) |
|  | TMLE-dmg | 0.315 (0.28, 0.35) | 0.313 (0.29, 0.34) | 0.297 (0.28, 0.31) | 0.276 (0.27, 0.29) |
|  | TMLE-dmg_saps | 0.505 (0.47, 0.54) | 0.406 (0.37, 0.44) | 0.329 (0.32, 0.34) | 0.24 (0.23, 0.25) |
| n_lab_grp6 | [prm]TMLE-dmg_sofa | 2.312 (2.24, 2.39) | 1.907 (1.84, 1.97) | 1.604 (1.58, 1.63) | 1.511 (1.49, 1.53) |
|  | GLM | 2.144 (2.07, 2.22) | 1.854 (1.77, 1.94) | 1.583 (1.51, 1.66) | 1.502 (1.43, 1.58) |
|  | GLM-sofa | 1.779 (1.71, 1.85) | 1.434 (1.35, 1.52) | 1.114 (1.04, 1.19) | 1.003 (0.93, 1.08) |
|  | GLM-saps | 1.533 (1.46, 1.61) | 1.117 (1.03, 1.2) | 0.679 (0.61, 0.75) | 0.367 (0.29, 0.44) |
|  | TMLE-dmg | 2.126 (2.04, 2.21) | 1.847 (1.79, 1.91) | 1.603 (1.58, 1.63) | 1.522 (1.5, 1.54) |
|  | TMLE-dmg_saps | 2.86 (2.78, 2.94) | 2.193 (2.11, 2.28) | 1.697 (1.67, 1.72) | 1.413 (1.4, 1.43) |
| n_lab_grp7 | [prm]TMLE-dmg_sofa | 2.097 (2, 2.19) | 1.82 (1.75, 1.89) | 1.751 (1.72, 1.78) | 1.671 (1.65, 1.69) |
|  | GLM | 1.814 (1.73, 1.9) | 1.716 (1.61, 1.82) | 1.739 (1.65, 1.83) | 1.686 (1.6, 1.78) |
|  | GLM-sofa | 1.301 (1.22, 1.39) | 1.128 (1.03, 1.23) | 1.081 (0.99, 1.17) | 0.986 (0.9, 1.07) |
|  | GLM-saps | 1.307 (1.22, 1.4) | 1.106 (1, 1.21) | 0.99 (0.9, 1.08) | 0.745 (0.65, 0.84) |
|  | TMLE-dmg | 1.844 (1.74, 1.94) | 1.735 (1.67, 1.8) | 1.741 (1.71, 1.77) | 1.697 (1.67, 1.72) |
|  | TMLE-dmg_saps | 2.633 (2.53, 2.73) | 2.038 (1.94, 2.13) | 1.829 (1.8, 1.86) | 1.6 (1.58, 1.62) |

####

#### Appendix G.2.1. Estimated Average Measurement Frequencies for Vital Signs across Race/Ethnicity Groups

| **Y** | **method** | **WHITE** | **ASIAN** | **BLACK** | **HISPANIC** | **Other** |
| --- | --- | --- | --- | --- | --- | --- |
| n_BP | [prm]TMLE-dmg_sofa | 34.217 (34, 34.44) | 34.452 (33.68, 35.22) | 32.789 (32.12, 33.46) | 32.4 (31.82, 32.98) | 33.737 (32.88, 34.59) |
|  | GLM | 34.075 (33.86, 34.29) | 33.768 (32.54, 35) | 32.294 (31.56, 33.03) | 32.119 (31.06, 33.18) | 33.823 (32.74, 34.9) |
|  | GLM-sofa | 29.356 (29.01, 29.7) | 28.829 (27.63, 30.03) | 27.513 (26.8, 28.23) | 27.69 (26.65, 28.73) | 29.283 (28.23, 30.34) |
|  | GLM-saps | 27.02 (26.48, 27.56) | 26.656 (25.45, 27.86) | 25.346 (24.62, 26.07) | 25.924 (24.88, 26.97) | 27.298 (26.24, 28.36) |
|  | TMLE-dmg | 34.209 (33.99, 34.43) | 34.253 (33.44, 35.07) | 32.981 (32.27, 33.69) | 31.978 (31.4, 32.56) | 33.691 (32.83, 34.56) |
|  | TMLE-dmg_saps | 34.225 (34.01, 34.44) | 34.136 (33.36, 34.91) | 32.752 (32.07, 33.44) | 32.307 (31.73, 32.88) | 33.88 (33, 34.76) |
| n_BPm | [prm]TMLE-dmg_sofa | 34.026 (33.81, 34.24) | 34.326 (33.55, 35.1) | 32.626 (31.94, 33.31) | 32.298 (31.73, 32.87) | 33.666 (32.81, 34.52) |
|  | GLM | 33.881 (33.67, 34.09) | 33.683 (32.46, 34.9) | 32.142 (31.41, 32.87) | 31.948 (30.89, 33) | 33.687 (32.61, 34.76) |
|  | GLM-sofa | 29.042 (28.7, 29.39) | 28.618 (27.43, 29.8) | 27.239 (26.53, 27.95) | 27.406 (26.38, 28.43) | 29.032 (27.99, 30.08) |
|  | GLM-saps | 26.599 (26.07, 27.13) | 26.341 (25.15, 27.54) | 24.969 (24.25, 25.68) | 25.553 (24.52, 26.59) | 26.952 (25.9, 28) |
|  | TMLE-dmg | 34.017 (33.8, 34.24) | 34.134 (33.32, 34.95) | 32.829 (32.11, 33.55) | 31.82 (31.24, 32.4) | 33.607 (32.74, 34.48) |
|  | TMLE-dmg_saps | 34.034 (33.82, 34.25) | 34.018 (33.25, 34.79) | 32.585 (31.89, 33.28) | 32.227 (31.66, 32.79) | 33.792 (32.91, 34.68) |
| n_GCS | [prm]TMLE-dmg_sofa | 3.156 (3.08, 3.23) | 4.023 (3.71, 4.34) | 3.551 (3.26, 3.85) | 4.08 (3.76, 4.4) | 4.455 (4.01, 4.9) |
|  | GLM | 3.148 (3.07, 3.22) | 3.795 (3.35, 4.24) | 3.545 (3.28, 3.81) | 4.033 (3.65, 4.41) | 4.284 (3.9, 4.67) |
|  | GLM-sofa | 3.595 (3.47, 3.72) | 4.264 (3.82, 4.7) | 3.998 (3.73, 4.26) | 4.453 (4.07, 4.83) | 4.714 (4.33, 5.1) |
|  | GLM-saps | 3.458 (3.26, 3.65) | 4.108 (3.67, 4.55) | 3.851 (3.59, 4.12) | 4.305 (3.92, 4.69) | 4.571 (4.18, 4.96) |
|  | TMLE-dmg | 3.157 (3.08, 3.23) | 3.973 (3.67, 4.28) | 3.536 (3.24, 3.83) | 4.045 (3.72, 4.37) | 4.511 (4.06, 4.96) |
|  | TMLE-dmg_saps | 3.158 (3.08, 3.23) | 4.012 (3.7, 4.32) | 3.56 (3.27, 3.85) | 4.071 (3.75, 4.39) | 4.46 (4.01, 4.91) |
| n_GCS_Total | [prm]TMLE-dmg_sofa | 5.03 (4.94, 5.12) | 4.361 (4.04, 4.68) | 4.801 (4.51, 5.09) | 4.668 (4.35, 4.99) | 4.223 (3.85, 4.6) |
|  | GLM | 4.973 (4.89, 5.06) | 4.484 (3.99, 4.98) | 4.707 (4.41, 5) | 4.85 (4.42, 5.28) | 4.524 (4.09, 4.96) |
|  | GLM-sofa | 5.844 (5.7, 5.99) | 5.395 (4.9, 5.89) | 5.589 (5.3, 5.88) | 5.668 (5.24, 6.09) | 5.362 (4.93, 5.79) |
|  | GLM-saps | 6.071 (5.85, 6.29) | 5.59 (5.1, 6.08) | 5.788 (5.49, 6.08) | 5.814 (5.39, 6.24) | 5.539 (5.11, 5.97) |
|  | TMLE-dmg | 5.031 (4.94, 5.12) | 4.442 (4.12, 4.76) | 4.786 (4.5, 5.07) | 4.729 (4.4, 5.05) | 4.197 (3.82, 4.57) |
|  | TMLE-dmg_saps | 5.028 (4.94, 5.12) | 4.404 (4.09, 4.72) | 4.818 (4.53, 5.11) | 4.691 (4.38, 5.01) | 4.187 (3.82, 4.56) |
| n_HR | [prm]TMLE-dmg_sofa | 31.069 (30.9, 31.24) | 31.16 (30.57, 31.75) | 30.11 (29.62, 30.6) | 29.63 (29.2, 30.06) | 30.79 (30.15, 31.43) |
|  | GLM | 30.92 (30.76, 31.08) | 31.043 (30.12, 31.97) | 29.752 (29.2, 30.31) | 29.351 (28.55, 30.15) | 30.676 (29.86, 31.49) |
|  | GLM-sofa | 27.42 (27.16, 27.68) | 27.38 (26.48, 28.28) | 26.207 (25.67, 26.75) | 26.067 (25.29, 26.85) | 27.309 (26.51, 28.1) |
|  | GLM-saps | 25.827 (25.42, 26.23) | 25.908 (25, 26.82) | 24.736 (24.19, 25.28) | 24.879 (24.09, 25.67) | 25.965 (25.16, 26.77) |
|  | TMLE-dmg | 31.066 (30.9, 31.23) | 31.085 (30.45, 31.72) | 30.232 (29.72, 30.75) | 29.321 (28.87, 29.77) | 30.773 (30.12, 31.42) |
|  | TMLE-dmg_saps | 31.076 (30.91, 31.24) | 30.936 (30.35, 31.52) | 30.077 (29.58, 30.58) | 29.559 (29.12, 30) | 30.848 (30.2, 31.5) |
| n_SpO2 | [prm]TMLE-dmg_sofa | 30.641 (30.48, 30.81) | 30.718 (30.12, 31.31) | 28.832 (28.32, 29.34) | 29.296 (28.85, 29.75) | 30.33 (29.67, 30.99) |
|  | GLM | 30.529 (30.37, 30.69) | 30.385 (29.46, 31.31) | 28.477 (27.92, 29.03) | 29.203 (28.4, 30.01) | 30.327 (29.51, 31.14) |
|  | GLM-sofa | 27.3 (27.04, 27.56) | 27.005 (26.1, 27.91) | 25.206 (24.66, 25.75) | 26.173 (25.39, 26.96) | 27.22 (26.42, 28.02) |
|  | GLM-saps | 26.115 (25.71, 26.52) | 25.935 (25.02, 26.85) | 24.13 (23.58, 24.68) | 25.328 (24.53, 26.12) | 26.244 (25.44, 27.05) |
|  | TMLE-dmg | 30.638 (30.47, 30.8) | 30.616 (29.98, 31.25) | 28.891 (28.37, 29.41) | 29.027 (28.56, 29.49) | 30.367 (29.7, 31.03) |
|  | TMLE-dmg_saps | 30.646 (30.48, 30.81) | 30.514 (29.93, 31.1) | 28.825 (28.31, 29.34) | 29.273 (28.82, 29.73) | 30.399 (29.72, 31.07) |
| n_RR | [prm]TMLE-dmg_sofa | 34.573 (34.38, 34.77) | 34.656 (33.97, 35.35) | 33.165 (32.57, 33.76) | 33.853 (33.32, 34.38) | 35.203 (34.44, 35.97) |
|  | GLM | 34.397 (34.21, 34.59) | 34.754 (33.66, 35.85) | 33.061 (32.41, 33.72) | 33.775 (32.83, 34.72) | 35.014 (34.05, 35.98) |
|  | GLM-sofa | 28.913 (28.61, 29.22) | 29.015 (27.97, 30.06) | 27.505 (26.88, 28.13) | 28.628 (27.72, 29.53) | 29.738 (28.82, 30.66) |
|  | GLM-saps | 26.001 (25.53, 26.47) | 26.29 (25.23, 27.35) | 24.792 (24.16, 25.42) | 26.402 (25.49, 27.32) | 27.248 (26.32, 28.18) |
|  | TMLE-dmg | 34.563 (34.37, 34.76) | 34.641 (33.89, 35.39) | 33.365 (32.74, 33.99) | 33.426 (32.88, 33.98) | 35.174 (34.38, 35.97) |
|  | TMLE-dmg_saps | 34.582 (34.39, 34.78) | 34.428 (33.75, 35.11) | 33.099 (32.5, 33.7) | 33.84 (33.3, 34.38) | 35.261 (34.47, 36.05) |
| n_TempC | [prm]TMLE-dmg_sofa | 11.694 (11.53, 11.86) | 11.046 (10.49, 11.6) | 9.645 (9.21, 10.08) | 11.099 (10.61, 11.59) | 11.546 (10.79, 12.31) |
|  | GLM | 11.753 (11.59, 11.91) | 11.198 (10.27, 12.12) | 9.341 (8.79, 9.89) | 10.666 (9.87, 11.47) | 11.318 (10.5, 12.13) |
|  | GLM-sofa | 9.391 (9.12, 9.66) | 8.725 (7.81, 9.64) | 6.948 (6.4, 7.49) | 8.449 (7.66, 9.24) | 9.045 (8.24, 9.85) |
|  | GLM-saps | 10.832 (10.42, 11.24) | 10.27 (9.35, 11.19) | 8.434 (7.88, 8.99) | 9.857 (9.06, 10.66) | 10.466 (9.65, 11.28) |
|  | TMLE-dmg | 11.698 (11.54, 11.86) | 10.88 (10.32, 11.44) | 9.649 (9.21, 10.08) | 10.934 (10.43, 11.44) | 11.617 (10.84, 12.39) |
|  | TMLE-dmg_saps | 11.693 (11.53, 11.86) | 10.935 (10.37, 11.5) | 9.639 (9.2, 10.08) | 11.085 (10.58, 11.59) | 11.574 (10.8, 12.35) |

####

#### Appendix G.2.2. Estimated Average Missing Data Rates for Vital Signs across Race/Ethnicity Groups

| **Y** | **method** | **WHITE** | **ASIAN** | **BLACK** | **HISPANIC** | **Other** |
| --- | --- | --- | --- | --- | --- | --- |
| h_m_BP | [prm]TMLE-dmg_sofa | 2.297 (2.25, 2.34) | 2.382 (2.2, 2.56) | 2.318 (2.16, 2.48) | 2.156 (2.01, 2.31) | 2.309 (2.13, 2.49) |
|  | GLM | 2.258 (2.22, 2.3) | 2.122 (1.88, 2.37) | 2.269 (2.12, 2.42) | 2.193 (1.98, 2.41) | 2.307 (2.09, 2.52) |
|  | GLM-sofa | 2.587 (2.52, 2.66) | 2.466 (2.22, 2.71) | 2.602 (2.46, 2.75) | 2.502 (2.29, 2.71) | 2.623 (2.41, 2.84) |
|  | GLM-saps | 2.987 (2.88, 3.1) | 2.856 (2.61, 3.1) | 2.987 (2.84, 3.13) | 2.833 (2.62, 3.04) | 2.981 (2.77, 3.2) |
|  | TMLE-dmg | 2.297 (2.25, 2.34) | 2.408 (2.22, 2.59) | 2.321 (2.16, 2.49) | 2.199 (2.05, 2.35) | 2.317 (2.14, 2.5) |
|  | TMLE-dmg_saps | 2.297 (2.25, 2.34) | 2.387 (2.21, 2.57) | 2.331 (2.17, 2.49) | 2.175 (2.02, 2.33) | 2.292 (2.11, 2.47) |
| h_m_BPm | [prm]TMLE-dmg_sofa | 2.381 (2.34, 2.43) | 2.417 (2.24, 2.6) | 2.435 (2.27, 2.6) | 2.23 (2.08, 2.38) | 2.338 (2.16, 2.52) |
|  | GLM | 2.342 (2.3, 2.39) | 2.162 (1.91, 2.42) | 2.371 (2.22, 2.52) | 2.287 (2.07, 2.51) | 2.338 (2.12, 2.56) |
|  | GLM-sofa | 2.697 (2.62, 2.77) | 2.535 (2.28, 2.79) | 2.731 (2.58, 2.88) | 2.62 (2.4, 2.84) | 2.68 (2.46, 2.9) |
|  | GLM-saps | 3.129 (3.02, 3.24) | 2.956 (2.7, 3.21) | 3.146 (3, 3.3) | 2.978 (2.76, 3.2) | 3.066 (2.84, 3.29) |
|  | TMLE-dmg | 2.381 (2.34, 2.43) | 2.451 (2.27, 2.64) | 2.433 (2.26, 2.6) | 2.271 (2.12, 2.43) | 2.348 (2.17, 2.53) |
|  | TMLE-dmg_saps | 2.38 (2.34, 2.43) | 2.423 (2.24, 2.6) | 2.451 (2.28, 2.62) | 2.246 (2.09, 2.4) | 2.327 (2.15, 2.51) |
| h_m_GCS | [prm]TMLE-dmg_sofa | 21.036 (20.93, 21.14) | 20.19 (19.9, 20.48) | 20.647 (20.34, 20.95) | 20.014 (19.7, 20.33) | 19.674 (19.24, 20.11) |
|  | GLM | 20.936 (20.83, 21.04) | 20.296 (19.7, 20.89) | 20.564 (20.21, 20.92) | 20.041 (19.52, 20.56) | 19.756 (19.23, 20.28) |
|  | GLM-sofa | 20.591 (20.42, 20.77) | 19.935 (19.34, 20.53) | 20.215 (19.86, 20.57) | 19.718 (19.2, 20.23) | 19.424 (18.9, 19.95) |
|  | GLM-saps | 20.712 (20.45, 20.98) | 20.07 (19.47, 20.67) | 20.343 (19.99, 20.7) | 19.845 (19.33, 20.36) | 19.549 (19.02, 20.07) |
|  | TMLE-dmg | 21.035 (20.93, 21.14) | 20.252 (19.96, 20.54) | 20.658 (20.35, 20.97) | 20.06 (19.73, 20.39) | 19.646 (19.21, 20.09) |
|  | TMLE-dmg_saps | 21.035 (20.93, 21.14) | 20.205 (19.92, 20.49) | 20.666 (20.36, 20.98) | 19.972 (19.65, 20.29) | 19.667 (19.23, 20.1) |
| h_m_GCS_Total | [prm]TMLE-dmg_sofa | 19.138 (19.05, 19.23) | 19.798 (19.49, 20.1) | 19.344 (19.05, 19.64) | 19.411 (19.1, 19.72) | 19.853 (19.48, 20.23) |
|  | GLM | 19.087 (19, 19.18) | 19.552 (19.04, 20.07) | 19.335 (19.03, 19.64) | 19.156 (18.71, 19.6) | 19.44 (18.99, 19.89) |
|  | GLM-sofa | 18.297 (18.15, 18.45) | 18.725 (18.21, 19.24) | 18.535 (18.23, 18.84) | 18.415 (17.97, 18.86) | 18.68 (18.23, 19.13) |
|  | GLM-saps | 18.058 (17.83, 18.29) | 18.514 (18, 19.03) | 18.321 (18.01, 18.63) | 18.252 (17.81, 18.7) | 18.487 (18.04, 18.94) |
|  | TMLE-dmg | 19.14 (19.05, 19.23) | 19.721 (19.41, 20.03) | 19.362 (19.07, 19.65) | 19.308 (18.99, 19.62) | 19.862 (19.49, 20.24) |
|  | TMLE-dmg_saps | 19.142 (19.05, 19.23) | 19.735 (19.43, 20.04) | 19.337 (19.04, 19.64) | 19.371 (19.06, 19.68) | 19.902 (19.53, 20.27) |
| h_m_HR | [prm]TMLE-dmg_sofa | 2.055 (2.01, 2.1) | 2.217 (2.04, 2.39) | 2.033 (1.87, 2.2) | 1.988 (1.84, 2.14) | 2.11 (1.94, 2.28) |
|  | GLM | 2.021 (1.98, 2.06) | 1.954 (1.72, 2.19) | 1.967 (1.82, 2.11) | 2.007 (1.8, 2.21) | 2.128 (1.92, 2.34) |
|  | GLM-sofa | 2.234 (2.16, 2.3) | 2.176 (1.94, 2.41) | 2.182 (2.04, 2.32) | 2.206 (2, 2.41) | 2.333 (2.12, 2.54) |
|  | GLM-saps | 2.603 (2.5, 2.71) | 2.54 (2.3, 2.78) | 2.539 (2.4, 2.68) | 2.517 (2.31, 2.72) | 2.666 (2.46, 2.88) |
|  | TMLE-dmg | 2.055 (2.01, 2.1) | 2.229 (2.05, 2.41) | 2.041 (1.87, 2.21) | 2.008 (1.86, 2.16) | 2.11 (1.94, 2.28) |
|  | TMLE-dmg_saps | 2.055 (2.01, 2.1) | 2.215 (2.04, 2.39) | 2.052 (1.88, 2.22) | 1.987 (1.83, 2.14) | 2.093 (1.92, 2.27) |
| h_m_SpO2 | [prm]TMLE-dmg_sofa | 2.608 (2.56, 2.66) | 2.651 (2.46, 2.84) | 3.053 (2.83, 3.27) | 2.514 (2.34, 2.69) | 2.626 (2.4, 2.85) |
|  | GLM | 2.562 (2.51, 2.61) | 2.477 (2.19, 2.76) | 2.992 (2.82, 3.16) | 2.451 (2.2, 2.7) | 2.601 (2.35, 2.85) |
|  | GLM-sofa | 2.728 (2.64, 2.81) | 2.651 (2.36, 2.94) | 3.16 (2.99, 3.33) | 2.608 (2.36, 2.86) | 2.762 (2.51, 3.01) |
|  | GLM-saps | 2.953 (2.83, 3.08) | 2.871 (2.58, 3.16) | 3.377 (3.2, 3.55) | 2.795 (2.55, 3.04) | 2.963 (2.71, 3.22) |
|  | TMLE-dmg | 2.606 (2.56, 2.66) | 2.676 (2.48, 2.87) | 3.087 (2.86, 3.32) | 2.531 (2.35, 2.71) | 2.621 (2.4, 2.84) |
|  | TMLE-dmg_saps | 2.607 (2.56, 2.66) | 2.63 (2.44, 2.82) | 3.047 (2.82, 3.27) | 2.534 (2.35, 2.71) | 2.617 (2.39, 2.84) |
| h_m_RR | [prm]TMLE-dmg_sofa | 2.364 (2.31, 2.42) | 2.782 (2.56, 3.01) | 2.429 (2.24, 2.62) | 2.166 (2, 2.33) | 2.329 (2.12, 2.54) |
|  | GLM | 2.32 (2.27, 2.37) | 2.379 (2.09, 2.67) | 2.284 (2.11, 2.46) | 2.169 (1.92, 2.42) | 2.361 (2.11, 2.62) |
|  | GLM-sofa | 2.521 (2.44, 2.6) | 2.589 (2.3, 2.88) | 2.487 (2.31, 2.66) | 2.358 (2.11, 2.61) | 2.553 (2.3, 2.81) |
|  | GLM-saps | 2.833 (2.7, 2.96) | 2.896 (2.61, 3.19) | 2.789 (2.62, 2.96) | 2.62 (2.37, 2.87) | 2.835 (2.58, 3.09) |
|  | TMLE-dmg | 2.364 (2.31, 2.42) | 2.824 (2.59, 3.05) | 2.432 (2.24, 2.63) | 2.177 (2.01, 2.35) | 2.336 (2.13, 2.54) |
|  | TMLE-dmg_saps | 2.366 (2.31, 2.42) | 2.745 (2.52, 2.97) | 2.44 (2.24, 2.64) | 2.199 (2.03, 2.37) | 2.322 (2.11, 2.53) |
| h_m_TempC | [prm]TMLE-dmg_sofa | 14.902 (14.79, 15.01) | 15.297 (15.02, 15.58) | 15.846 (15.58, 16.11) | 14.893 (14.6, 15.19) | 14.81 (14.38, 15.24) |
|  | GLM | 14.788 (14.68, 14.89) | 14.885 (14.28, 15.5) | 15.927 (15.56, 16.29) | 15.066 (14.54, 15.59) | 14.825 (14.29, 15.36) |
|  | GLM-sofa | 16.191 (16.02, 16.37) | 16.354 (15.75, 16.96) | 17.349 (16.99, 17.71) | 16.384 (15.86, 16.91) | 16.175 (15.64, 16.71) |
|  | GLM-saps | 15.412 (15.14, 15.68) | 15.514 (14.9, 16.12) | 16.541 (16.18, 16.91) | 15.614 (15.09, 16.14) | 15.402 (14.87, 15.94) |
|  | TMLE-dmg | 14.902 (14.79, 15.01) | 15.354 (15.07, 15.64) | 15.818 (15.54, 16.09) | 14.973 (14.67, 15.28) | 14.774 (14.33, 15.21) |
|  | TMLE-dmg_saps | 14.905 (14.79, 15.01) | 15.342 (15.06, 15.63) | 15.853 (15.57, 16.13) | 14.914 (14.61, 15.22) | 14.81 (14.37, 15.25) |

####

#### Appendix G.2.3. Estimated Average Measurement Frequencies for Lab Tests across Race/Ethnicity Groups

| **Y** | **method** | **WHITE** | **ASIAN** | **BLACK** | **HISPANIC** | **Other** |
| --- | --- | --- | --- | --- | --- | --- |
| n_HCT | [prm]TMLE-dmg_sofa | 2.754 (2.73, 2.78) | 2.91 (2.82, 3) | 2.486 (2.4, 2.57) | 2.483 (2.4, 2.56) | 2.761 (2.63, 2.89) |
|  | GLM | 2.743 (2.72, 2.77) | 2.89 (2.73, 3.05) | 2.483 (2.39, 2.58) | 2.654 (2.51, 2.79) | 2.75 (2.61, 2.89) |
|  | GLM-sofa | 2.074 (2.03, 2.12) | 2.19 (2.03, 2.35) | 1.805 (1.71, 1.9) | 2.026 (1.89, 2.16) | 2.107 (1.97, 2.24) |
|  | GLM-saps | 2.167 (2.1, 2.24) | 2.308 (2.15, 2.47) | 1.915 (1.82, 2.01) | 2.148 (2.01, 2.29) | 2.217 (2.08, 2.36) |
|  | TMLE-dmg | 2.753 (2.72, 2.78) | 2.896 (2.8, 2.99) | 2.514 (2.43, 2.6) | 2.432 (2.35, 2.51) | 2.752 (2.62, 2.88) |
|  | TMLE-dmg_saps | 2.755 (2.73, 2.78) | 2.908 (2.81, 3) | 2.493 (2.41, 2.58) | 2.465 (2.38, 2.55) | 2.756 (2.63, 2.89) |
| n_Lac | [prm]TMLE-dmg_sofa | 1.615 (1.58, 1.65) | 1.925 (1.81, 2.04) | 1.544 (1.44, 1.65) | 1.59 (1.49, 1.69) | 1.835 (1.67, 2) |
|  | GLM | 1.574 (1.54, 1.61) | 1.829 (1.63, 2.03) | 1.589 (1.47, 1.71) | 1.73 (1.56, 1.9) | 1.791 (1.62, 1.97) |
|  | GLM-sofa | 0.14 (0.09, 0.19) | 0.328 (0.15, 0.51) | 0.136 (0.03, 0.24) | 0.384 (0.23, 0.54) | 0.411 (0.25, 0.57) |
|  | GLM-saps | -0.254 (-0.34, -0.17) | -0.014 (-0.2, 0.18) | -0.211 (-0.32, -0.1) | 0.124 (-0.04, 0.29) | 0.1 (-0.07, 0.27) |
|  | TMLE-dmg | 1.611 (1.58, 1.65) | 1.879 (1.75, 2.01) | 1.607 (1.49, 1.73) | 1.487 (1.38, 1.59) | 1.817 (1.63, 2) |
|  | TMLE-dmg_saps | 1.617 (1.58, 1.65) | 1.872 (1.75, 1.99) | 1.534 (1.42, 1.64) | 1.568 (1.46, 1.67) | 1.837 (1.66, 2.01) |
| n_lab_grp1 | [prm]TMLE-dmg_sofa | 0.484 (0.47, 0.5) | 0.502 (0.46, 0.54) | 0.499 (0.46, 0.54) | 0.559 (0.51, 0.61) | 0.561 (0.5, 0.62) |
|  | GLM | 0.464 (0.45, 0.48) | 0.563 (0.49, 0.64) | 0.519 (0.48, 0.56) | 0.576 (0.51, 0.64) | 0.565 (0.5, 0.63) |
|  | GLM-sofa | 0.038 (0.02, 0.06) | 0.117 (0.05, 0.19) | 0.088 (0.05, 0.13) | 0.176 (0.12, 0.23) | 0.155 (0.1, 0.21) |
|  | GLM-saps | 0.008 (-0.02, 0.04) | 0.104 (0.03, 0.17) | 0.071 (0.03, 0.11) | 0.176 (0.11, 0.24) | 0.144 (0.08, 0.21) |
|  | TMLE-dmg | 0.484 (0.47, 0.5) | 0.493 (0.45, 0.54) | 0.518 (0.47, 0.56) | 0.525 (0.47, 0.58) | 0.548 (0.48, 0.61) |
|  | TMLE-dmg_saps | 0.484 (0.47, 0.5) | 0.487 (0.45, 0.53) | 0.494 (0.45, 0.54) | 0.548 (0.49, 0.6) | 0.561 (0.5, 0.63) |
| n_lab_grp2 | [prm]TMLE-dmg_sofa | 4.277 (4.22, 4.34) | 3.899 (3.72, 4.08) | 3.431 (3.26, 3.61) | 3.662 (3.49, 3.83) | 4.55 (4.27, 4.83) |
|  | GLM | 4.264 (4.21, 4.32) | 4.059 (3.72, 4.4) | 3.437 (3.23, 3.64) | 3.982 (3.69, 4.28) | 4.412 (4.11, 4.71) |
|  | GLM-sofa | 1.939 (1.85, 2.03) | 1.625 (1.31, 1.94) | 1.082 (0.9, 1.27) | 1.8 (1.53, 2.07) | 2.175 (1.9, 2.45) |
|  | GLM-saps | 1.981 (1.83, 2.13) | 1.756 (1.43, 2.09) | 1.188 (0.99, 1.39) | 1.976 (1.69, 2.26) | 2.3 (2.01, 2.59) |
|  | TMLE-dmg | 4.271 (4.21, 4.33) | 3.863 (3.66, 4.06) | 3.495 (3.3, 3.69) | 3.527 (3.35, 3.71) | 4.549 (4.24, 4.86) |
|  | TMLE-dmg_saps | 4.278 (4.22, 4.34) | 3.848 (3.66, 4.04) | 3.44 (3.26, 3.62) | 3.658 (3.48, 3.83) | 4.566 (4.27, 4.87) |
| n_lab_grp3 | [prm]TMLE-dmg_sofa | 1.985 (1.97, 2) | 2.143 (2.08, 2.21) | 2.102 (2.03, 2.17) | 2.001 (1.94, 2.06) | 2.053 (1.98, 2.13) |
|  | GLM | 1.964 (1.95, 1.98) | 2.121 (2.02, 2.22) | 2.109 (2.05, 2.17) | 2.026 (1.94, 2.11) | 2.049 (1.96, 2.13) |
|  | GLM-sofa | 1.451 (1.42, 1.48) | 1.585 (1.49, 1.68) | 1.59 (1.54, 1.64) | 1.545 (1.47, 1.62) | 1.556 (1.48, 1.64) |
|  | GLM-saps | 1.231 (1.19, 1.27) | 1.383 (1.29, 1.48) | 1.388 (1.33, 1.44) | 1.382 (1.3, 1.46) | 1.372 (1.29, 1.45) |
|  | TMLE-dmg | 1.984 (1.97, 2) | 2.129 (2.06, 2.2) | 2.12 (2.05, 2.19) | 1.956 (1.9, 2.02) | 2.044 (1.96, 2.13) |
|  | TMLE-dmg_saps | 1.986 (1.97, 2) | 2.131 (2.07, 2.19) | 2.081 (2.01, 2.15) | 1.994 (1.93, 2.05) | 2.058 (1.98, 2.14) |
| n_lab_grp4 | [prm]TMLE-dmg_sofa | 2.013 (2, 2.03) | 2.102 (2.04, 2.16) | 1.881 (1.82, 1.94) | 1.915 (1.86, 1.97) | 2.017 (1.93, 2.1) |
|  | GLM | 2.002 (1.98, 2.02) | 2.115 (2.01, 2.22) | 1.905 (1.84, 1.97) | 1.995 (1.91, 2.08) | 1.987 (1.9, 2.08) |
|  | GLM-sofa | 1.427 (1.4, 1.45) | 1.513 (1.42, 1.61) | 1.322 (1.26, 1.38) | 1.455 (1.37, 1.54) | 1.433 (1.35, 1.52) |
|  | GLM-saps | 1.425 (1.38, 1.47) | 1.533 (1.43, 1.63) | 1.337 (1.28, 1.4) | 1.488 (1.4, 1.57) | 1.453 (1.37, 1.54) |
|  | TMLE-dmg | 2.012 (1.99, 2.03) | 2.09 (2.02, 2.16) | 1.907 (1.84, 1.97) | 1.873 (1.81, 1.93) | 2.005 (1.91, 2.1) |
|  | TMLE-dmg_saps | 2.013 (2, 2.03) | 2.098 (2.03, 2.16) | 1.883 (1.82, 1.95) | 1.895 (1.84, 1.96) | 2.012 (1.92, 2.1) |
| n_lab_grp5 | [prm]TMLE-dmg_sofa | 0.29 (0.28, 0.3) | 0.317 (0.29, 0.35) | 0.291 (0.26, 0.32) | 0.284 (0.25, 0.32) | 0.246 (0.21, 0.28) |
|  | GLM | 0.28 (0.27, 0.29) | 0.33 (0.28, 0.38) | 0.312 (0.28, 0.34) | 0.321 (0.28, 0.36) | 0.258 (0.21, 0.3) |
|  | GLM-sofa | 0.12 (0.11, 0.13) | 0.163 (0.11, 0.21) | 0.15 (0.12, 0.18) | 0.171 (0.13, 0.21) | 0.104 (0.06, 0.15) |
|  | GLM-saps | 0.024 (0, 0.04) | 0.072 (0.02, 0.12) | 0.06 (0.03, 0.09) | 0.096 (0.05, 0.14) | 0.021 (-0.02, 0.06) |
|  | TMLE-dmg | 0.29 (0.28, 0.3) | 0.314 (0.28, 0.35) | 0.301 (0.27, 0.33) | 0.28 (0.25, 0.31) | 0.243 (0.21, 0.28) |
|  | TMLE-dmg_saps | 0.29 (0.28, 0.3) | 0.313 (0.28, 0.34) | 0.285 (0.26, 0.31) | 0.29 (0.26, 0.32) | 0.249 (0.21, 0.28) |
| n_lab_grp6 | [prm]TMLE-dmg_sofa | 1.6 (1.58, 1.62) | 1.715 (1.65, 1.78) | 1.813 (1.74, 1.89) | 1.628 (1.57, 1.68) | 1.714 (1.63, 1.8) |
|  | GLM | 1.572 (1.55, 1.59) | 1.727 (1.62, 1.83) | 1.832 (1.77, 1.89) | 1.653 (1.56, 1.74) | 1.72 (1.63, 1.81) |
|  | GLM-sofa | 1.132 (1.1, 1.16) | 1.266 (1.17, 1.37) | 1.386 (1.33, 1.45) | 1.24 (1.15, 1.33) | 1.297 (1.21, 1.38) |
|  | GLM-saps | 0.882 (0.84, 0.93) | 1.031 (0.93, 1.13) | 1.152 (1.09, 1.21) | 1.046 (0.96, 1.13) | 1.082 (0.99, 1.17) |
|  | TMLE-dmg | 1.599 (1.58, 1.62) | 1.701 (1.63, 1.77) | 1.828 (1.75, 1.9) | 1.586 (1.53, 1.65) | 1.702 (1.62, 1.79) |
|  | TMLE-dmg_saps | 1.602 (1.58, 1.62) | 1.697 (1.63, 1.76) | 1.786 (1.72, 1.86) | 1.618 (1.56, 1.68) | 1.714 (1.63, 1.8) |
| n_lab_grp7 | [prm]TMLE-dmg_sofa | 1.73 (1.71, 1.75) | 1.691 (1.62, 1.76) | 1.547 (1.48, 1.61) | 1.662 (1.59, 1.74) | 1.723 (1.62, 1.83) |
|  | GLM | 1.718 (1.7, 1.74) | 1.741 (1.62, 1.86) | 1.558 (1.48, 1.63) | 1.697 (1.59, 1.8) | 1.707 (1.6, 1.81) |
|  | GLM-sofa | 1.059 (1.03, 1.09) | 1.051 (0.93, 1.17) | 0.89 (0.82, 0.96) | 1.078 (0.98, 1.18) | 1.072 (0.97, 1.18) |
|  | GLM-saps | 1.053 (1, 1.11) | 1.07 (0.95, 1.19) | 0.903 (0.83, 0.98) | 1.112 (1.01, 1.22) | 1.091 (0.98, 1.2) |
|  | TMLE-dmg | 1.729 (1.71, 1.75) | 1.679 (1.6, 1.75) | 1.577 (1.51, 1.65) | 1.605 (1.53, 1.68) | 1.708 (1.6, 1.82) |
|  | TMLE-dmg_saps | 1.73 (1.71, 1.75) | 1.687 (1.61, 1.76) | 1.543 (1.48, 1.61) | 1.644 (1.57, 1.72) | 1.723 (1.61, 1.84) |

####

#### Appendix G.3.1. Estimated Average Measurement Frequencies for Vital Signs across Gender Groups

| **Y** | **method** | **Male** | **Female** |
| --- | --- | --- | --- |
| n_BP | [prm]TMLE-dmg_sofa | 34.48 (34.21, 34.75) | 33.982 (33.72, 34.24) |
|  | GLM | 34.41 (34.18, 34.64) | 33.431 (33.07, 33.79) |
|  | GLM-sofa | 29.471 (29.12, 29.82) | 28.871 (28.52, 29.22) |
|  | GLM-saps | 27.07 (26.57, 27.57) | 25.791 (25.44, 26.15) |
|  | TMLE-dmg | 34.72 (34.44, 35) | 33.738 (33.48, 34) |
|  | TMLE-dmg_saps | 34.688 (34.41, 34.97) | 33.719 (33.46, 33.98) |
| n_BPm | [prm]TMLE-dmg_sofa | 34.263 (34, 34.53) | 33.847 (33.58, 34.11) |
|  | GLM | 34.191 (33.96, 34.42) | 33.295 (32.94, 33.65) |
|  | GLM-sofa | 29.119 (28.77, 29.47) | 28.613 (28.26, 28.96) |
|  | GLM-saps | 26.63 (26.13, 27.13) | 25.426 (25.07, 25.78) |
|  | TMLE-dmg | 34.504 (34.23, 34.78) | 33.596 (33.33, 33.86) |
|  | TMLE-dmg_saps | 34.471 (34.19, 34.75) | 33.574 (33.31, 33.83) |
| n_GCS | [prm]TMLE-dmg_sofa | 3.04 (2.96, 3.12) | 3.236 (3.13, 3.34) |
|  | GLM | 3 (2.92, 3.08) | 3.242 (3.11, 3.37) |
|  | GLM-sofa | 3.383 (3.25, 3.51) | 3.595 (3.47, 3.72) |
|  | GLM-saps | 3.262 (3.08, 3.44) | 3.514 (3.39, 3.64) |
|  | TMLE-dmg | 3.021 (2.94, 3.1) | 3.268 (3.16, 3.37) |
|  | TMLE-dmg_saps | 3.028 (2.95, 3.11) | 3.258 (3.15, 3.36) |
| n_GCS_Total | [prm]TMLE-dmg_sofa | 4.99 (4.89, 5.09) | 5.11 (5, 5.22) |
|  | GLM | 4.958 (4.87, 5.05) | 5.063 (4.92, 5.21) |
|  | GLM-sofa | 5.868 (5.73, 6.01) | 5.903 (5.76, 6.04) |
|  | GLM-saps | 6.039 (5.84, 6.24) | 6.187 (6.05, 6.33) |
|  | TMLE-dmg | 4.968 (4.86, 5.07) | 5.155 (5.04, 5.27) |
|  | TMLE-dmg_saps | 4.963 (4.86, 5.07) | 5.165 (5.05, 5.28) |
| n_HR | [prm]TMLE-dmg_sofa | 31.231 (31.02, 31.45) | 31.081 (30.89, 31.28) |
|  | GLM | 31.133 (30.96, 31.31) | 30.658 (30.38, 30.93) |
|  | GLM-sofa | 27.476 (27.21, 27.74) | 27.282 (27.01, 27.55) |
|  | GLM-saps | 25.869 (25.49, 26.25) | 25.179 (24.91, 25.45) |
|  | TMLE-dmg | 31.407 (31.18, 31.63) | 30.888 (30.69, 31.08) |
|  | TMLE-dmg_saps | 31.381 (31.16, 31.6) | 30.874 (30.68, 31.07) |
| n_SpO2 | [prm]TMLE-dmg_sofa | 30.736 (30.53, 30.95) | 30.497 (30.3, 30.7) |
|  | GLM | 30.727 (30.55, 30.9) | 30.084 (29.81, 30.36) |
|  | GLM-sofa | 27.375 (27.11, 27.64) | 26.99 (26.72, 27.26) |
|  | GLM-saps | 26.162 (25.78, 26.55) | 25.333 (25.06, 25.6) |
|  | TMLE-dmg | 30.912 (30.69, 31.13) | 30.308 (30.11, 30.5) |
|  | TMLE-dmg_saps | 30.894 (30.68, 31.11) | 30.282 (30.08, 30.48) |
| n_RR | [prm]TMLE-dmg_sofa | 34.75 (34.5, 35) | 34.642 (34.41, 34.87) |
|  | GLM | 34.717 (34.51, 34.92) | 34.075 (33.75, 34.4) |
|  | GLM-sofa | 29.047 (28.74, 29.35) | 28.842 (28.53, 29.15) |
|  | GLM-saps | 26.292 (25.85, 26.73) | 25.308 (25, 25.62) |
|  | TMLE-dmg | 35.005 (34.75, 35.26) | 34.365 (34.13, 34.6) |
|  | TMLE-dmg_saps | 34.98 (34.73, 35.23) | 34.337 (34.11, 34.57) |
| n_TempC | [prm]TMLE-dmg_sofa | 12.216 (12.03, 12.4) | 11.264 (11.05, 11.48) |
|  | GLM | 12.487 (12.3, 12.67) | 11.015 (10.73, 11.3) |
|  | GLM-sofa | 10.024 (9.75, 10.3) | 8.741 (8.46, 9.02) |
|  | GLM-saps | 11.332 (10.93, 11.73) | 9.813 (9.53, 10.09) |
|  | TMLE-dmg | 12.372 (12.19, 12.56) | 11.071 (10.86, 11.28) |
|  | TMLE-dmg_saps | 12.355 (12.17, 12.54) | 11.075 (10.86, 11.29) |

#### Appendix G.3.2. Estimated Average Missing Data Rates for Vital Signs across Gender Groups

| **Y** | **method** | **Male** | **Female** |
| --- | --- | --- | --- |
| h_m_BP | [prm]TMLE-dmg_sofa | 2.4 (2.35, 2.45) | 2.214 (2.16, 2.27) |
|  | GLM | 2.365 (2.32, 2.41) | 2.184 (2.11, 2.26) |
|  | GLM-sofa | 2.694 (2.62, 2.77) | 2.488 (2.41, 2.56) |
|  | GLM-saps | 3.062 (2.96, 3.17) | 2.909 (2.84, 2.98) |
|  | TMLE-dmg | 2.388 (2.34, 2.44) | 2.233 (2.18, 2.29) |
|  | TMLE-dmg_saps | 2.388 (2.34, 2.44) | 2.24 (2.18, 2.3) |
| h_m_BPm | [prm]TMLE-dmg_sofa | 2.483 (2.43, 2.54) | 2.299 (2.24, 2.36) |
|  | GLM | 2.448 (2.4, 2.5) | 2.263 (2.19, 2.34) |
|  | GLM-sofa | 2.805 (2.73, 2.88) | 2.592 (2.52, 2.67) |
|  | GLM-saps | 3.197 (3.09, 3.3) | 3.043 (2.97, 3.12) |
|  | TMLE-dmg | 2.47 (2.42, 2.52) | 2.32 (2.26, 2.38) |
|  | TMLE-dmg_saps | 2.472 (2.42, 2.52) | 2.326 (2.27, 2.39) |
| h_m_GCS | [prm]TMLE-dmg_sofa | 21.207 (21.05, 21.36) | 20.92 (20.82, 21.02) |
|  | GLM | 21.105 (21, 21.21) | 20.802 (20.63, 20.97) |
|  | GLM-sofa | 20.83 (20.66, 21) | 20.548 (20.38, 20.72) |
|  | GLM-saps | 20.916 (20.68, 21.15) | 20.606 (20.44, 20.77) |
|  | TMLE-dmg | 21.233 (21.07, 21.4) | 20.893 (20.79, 21) |
|  | TMLE-dmg_saps | 21.223 (21.06, 21.39) | 20.903 (20.8, 21.01) |
| h_m_GCS_Total | [prm]TMLE-dmg_sofa | 19.231 (19.12, 19.35) | 19.02 (18.91, 19.13) |
|  | GLM | 19.124 (19.03, 19.22) | 18.95 (18.8, 19.1) |
|  | GLM-sofa | 18.302 (18.15, 18.45) | 18.191 (18.04, 18.34) |
|  | GLM-saps | 18.101 (17.89, 18.31) | 17.886 (17.74, 18.03) |
|  | TMLE-dmg | 19.263 (19.14, 19.38) | 18.974 (18.86, 19.08) |
|  | TMLE-dmg_saps | 19.262 (19.14, 19.38) | 18.969 (18.86, 19.08) |
| h_m_HR | [prm]TMLE-dmg_sofa | 2.181 (2.13, 2.23) | 1.959 (1.91, 2.01) |
|  | GLM | 2.161 (2.11, 2.21) | 1.917 (1.84, 1.99) |
|  | GLM-sofa | 2.38 (2.31, 2.45) | 2.119 (2.05, 2.19) |
|  | GLM-saps | 2.72 (2.62, 2.82) | 2.499 (2.43, 2.57) |
|  | TMLE-dmg | 2.176 (2.13, 2.23) | 1.97 (1.92, 2.02) |
|  | TMLE-dmg_saps | 2.175 (2.13, 2.22) | 1.977 (1.92, 2.03) |
| h_m_SpO2 | [prm]TMLE-dmg_sofa | 2.782 (2.72, 2.84) | 2.587 (2.52, 2.65) |
|  | GLM | 2.732 (2.68, 2.79) | 2.552 (2.47, 2.64) |
|  | GLM-sofa | 2.917 (2.83, 3) | 2.722 (2.64, 2.81) |
|  | GLM-saps | 3.114 (2.99, 3.24) | 2.949 (2.86, 3.04) |
|  | TMLE-dmg | 2.771 (2.71, 2.83) | 2.606 (2.54, 2.67) |
|  | TMLE-dmg_saps | 2.768 (2.71, 2.83) | 2.617 (2.55, 2.69) |
| h_m_RR | [prm]TMLE-dmg_sofa | 2.478 (2.42, 2.54) | 2.298 (2.23, 2.36) |
|  | GLM | 2.453 (2.4, 2.51) | 2.244 (2.16, 2.33) |
|  | GLM-sofa | 2.659 (2.57, 2.75) | 2.434 (2.35, 2.52) |
|  | GLM-saps | 2.947 (2.82, 3.07) | 2.758 (2.67, 2.84) |
|  | TMLE-dmg | 2.475 (2.42, 2.53) | 2.304 (2.24, 2.37) |
|  | TMLE-dmg_saps | 2.475 (2.42, 2.53) | 2.31 (2.24, 2.38) |
| h_m_TempC | [prm]TMLE-dmg_sofa | 14.698 (14.55, 14.85) | 15.136 (15.02, 15.25) |
|  | GLM | 14.43 (14.31, 14.54) | 15.166 (14.99, 15.34) |
|  | GLM-sofa | 15.864 (15.69, 16.04) | 16.49 (16.31, 16.67) |
|  | GLM-saps | 15.135 (14.88, 15.39) | 15.9 (15.72, 16.08) |
|  | TMLE-dmg | 14.618 (14.46, 14.78) | 15.241 (15.13, 15.35) |
|  | TMLE-dmg_saps | 14.628 (14.47, 14.78) | 15.243 (15.13, 15.36) |

####

#### Appendix G.3.3. Estimated Average Measurement Frequencies for Lab Tests across Gender Groups

| **Y** | **method** | **Male** | **Female** |
| --- | --- | --- | --- |
| n_HCT | [prm]TMLE-dmg_sofa | 2.776 (2.74, 2.81) | 2.644 (2.61, 2.68) |
|  | GLM | 2.809 (2.78, 2.84) | 2.588 (2.54, 2.63) |
|  | GLM-sofa | 2.122 (2.08, 2.17) | 1.953 (1.91, 2) |
|  | GLM-saps | 2.214 (2.15, 2.28) | 1.968 (1.92, 2.01) |
|  | TMLE-dmg | 2.809 (2.77, 2.85) | 2.606 (2.57, 2.64) |
|  | TMLE-dmg_saps | 2.806 (2.77, 2.84) | 2.603 (2.57, 2.64) |
| n_Lac | [prm]TMLE-dmg_sofa | 1.558 (1.52, 1.6) | 1.621 (1.58, 1.66) |
|  | GLM | 1.583 (1.55, 1.62) | 1.52 (1.46, 1.58) |
|  | GLM-sofa | 0.097 (0.04, 0.15) | 0.148 (0.1, 0.2) |
|  | GLM-saps | -0.21 (-0.29, -0.13) | -0.346 (-0.4, -0.29) |
|  | TMLE-dmg | 1.615 (1.57, 1.65) | 1.55 (1.51, 1.59) |
|  | TMLE-dmg_saps | 1.612 (1.57, 1.65) | 1.545 (1.5, 1.59) |
| n_lab_grp1 | [prm]TMLE-dmg_sofa | 0.472 (0.46, 0.48) | 0.524 (0.51, 0.54) |
|  | GLM | 0.479 (0.47, 0.49) | 0.487 (0.47, 0.51) |
|  | GLM-sofa | 0.034 (0.01, 0.05) | 0.076 (0.06, 0.1) |
|  | GLM-saps | 0.036 (0.01, 0.07) | 0.026 (0, 0.05) |
|  | TMLE-dmg | 0.485 (0.47, 0.5) | 0.504 (0.49, 0.52) |
|  | TMLE-dmg_saps | 0.484 (0.47, 0.5) | 0.502 (0.49, 0.52) |
| n_lab_grp2 | [prm]TMLE-dmg_sofa | 4.401 (4.34, 4.46) | 4.171 (4.1, 4.24) |
|  | GLM | 4.547 (4.48, 4.61) | 3.964 (3.86, 4.06) |
|  | GLM-sofa | 2.13 (2.04, 2.22) | 1.733 (1.64, 1.83) |
|  | GLM-saps | 2.197 (2.06, 2.34) | 1.518 (1.42, 1.62) |
|  | TMLE-dmg | 4.51 (4.44, 4.58) | 4.029 (3.95, 4.1) |
|  | TMLE-dmg_saps | 4.507 (4.44, 4.57) | 4.024 (3.95, 4.1) |
| n_lab_grp3 | [prm]TMLE-dmg_sofa | 1.97 (1.95, 1.99) | 2.018 (2, 2.04) |
|  | GLM | 1.975 (1.96, 1.99) | 1.972 (1.94, 2) |
|  | GLM-sofa | 1.443 (1.42, 1.47) | 1.482 (1.45, 1.51) |
|  | GLM-saps | 1.261 (1.22, 1.3) | 1.23 (1.2, 1.26) |
|  | TMLE-dmg | 1.991 (1.97, 2.01) | 1.992 (1.97, 2.01) |
|  | TMLE-dmg_saps | 1.99 (1.97, 2.01) | 1.989 (1.97, 2.01) |
| n_lab_grp4 | [prm]TMLE-dmg_sofa | 2.012 (1.99, 2.03) | 1.959 (1.94, 1.98) |
|  | GLM | 2.033 (2.01, 2.05) | 1.914 (1.88, 1.94) |
|  | GLM-sofa | 1.449 (1.42, 1.48) | 1.375 (1.35, 1.4) |
|  | GLM-saps | 1.46 (1.42, 1.5) | 1.318 (1.29, 1.35) |
|  | TMLE-dmg | 2.036 (2.02, 2.06) | 1.929 (1.91, 1.95) |
|  | TMLE-dmg_saps | 2.035 (2.02, 2.05) | 1.927 (1.9, 1.95) |
| n_lab_grp5 | [prm]TMLE-dmg_sofa | 0.274 (0.26, 0.28) | 0.316 (0.3, 0.33) |
|  | GLM | 0.27 (0.26, 0.28) | 0.304 (0.29, 0.32) |
|  | GLM-sofa | 0.106 (0.09, 0.12) | 0.153 (0.14, 0.17) |
|  | GLM-saps | 0.029 (0.01, 0.05) | 0.053 (0.04, 0.07) |
|  | TMLE-dmg | 0.278 (0.27, 0.29) | 0.308 (0.3, 0.32) |
|  | TMLE-dmg_saps | 0.278 (0.27, 0.29) | 0.308 (0.3, 0.32) |
| n_lab_grp6 | [prm]TMLE-dmg_sofa | 1.557 (1.54, 1.58) | 1.682 (1.66, 1.71) |
|  | GLM | 1.549 (1.53, 1.57) | 1.645 (1.61, 1.68) |
|  | GLM-sofa | 1.081 (1.05, 1.11) | 1.213 (1.18, 1.24) |
|  | GLM-saps | 0.882 (0.84, 0.92) | 0.951 (0.92, 0.98) |
|  | TMLE-dmg | 1.572 (1.55, 1.59) | 1.662 (1.64, 1.69) |
|  | TMLE-dmg_saps | 1.571 (1.55, 1.59) | 1.657 (1.63, 1.68) |
| n_lab_grp7 | [prm]TMLE-dmg_sofa | 1.751 (1.73, 1.77) | 1.676 (1.65, 1.7) |
|  | GLM | 1.776 (1.75, 1.8) | 1.623 (1.59, 1.66) |
|  | GLM-sofa | 1.103 (1.07, 1.14) | 1.002 (0.97, 1.04) |
|  | GLM-saps | 1.121 (1.07, 1.17) | 0.941 (0.91, 0.98) |
|  | TMLE-dmg | 1.776 (1.75, 1.8) | 1.642 (1.61, 1.67) |
|  | TMLE-dmg_saps | 1.775 (1.75, 1.8) | 1.64 (1.61, 1.67) |

#### Appendix G.4.1. Estimated Average Measurement Frequencies for Vital Signs across Insurance Groups

| **Y** | **method** | **No Insurance** | **Insurance** |
| --- | --- | --- | --- |
| n_BP | [prm]TMLE-dmg_sofa | 35.105 (34.46, 35.75) | 34.239 (34.06, 34.42) |
|  | GLM | 34.424 (32.72, 36.13) | 34 (32.28, 35.72) |
|  | GLM-sofa | 30.06 (28.38, 31.74) | 29.185 (27.51, 30.86) |
|  | GLM-saps | 28.402 (26.69, 30.12) | 26.579 (24.89, 28.26) |
|  | TMLE-dmg | 34.731 (34.07, 35.4) | 34.239 (34.05, 34.42) |
|  | TMLE-dmg_saps | 34.521 (33.89, 35.15) | 34.241 (34.06, 34.43) |
| n_BPm | [prm]TMLE-dmg_sofa | 34.911 (34.27, 35.56) | 34.061 (33.88, 34.24) |
|  | GLM | 34.164 (32.47, 35.86) | 33.816 (32.11, 35.52) |
|  | GLM-sofa | 29.687 (28.02, 31.35) | 28.877 (27.22, 30.54) |
|  | GLM-saps | 27.956 (26.25, 29.66) | 26.166 (24.49, 27.84) |
|  | TMLE-dmg | 34.525 (33.86, 35.19) | 34.061 (33.88, 34.24) |
|  | TMLE-dmg_saps | 34.329 (33.7, 34.96) | 34.063 (33.88, 34.25) |
| n_GCS | [prm]TMLE-dmg_sofa | 2.883 (2.66, 3.11) | 3.117 (3.05, 3.18) |
|  | GLM | 2.987 (2.38, 3.6) | 3.102 (2.49, 3.71) |
|  | GLM-sofa | 3.332 (2.72, 3.95) | 3.482 (2.87, 4.09) |
|  | GLM-saps | 3.192 (2.57, 3.82) | 3.354 (2.74, 3.97) |
|  | TMLE-dmg | 2.906 (2.68, 3.13) | 3.116 (3.05, 3.18) |
|  | TMLE-dmg_saps | 2.963 (2.73, 3.19) | 3.117 (3.05, 3.18) |
| n_GCS_Total | [prm]TMLE-dmg_sofa | 6.228 (5.96, 6.5) | 5.016 (4.94, 5.09) |
|  | GLM | 6.594 (5.92, 7.27) | 4.985 (4.31, 5.66) |
|  | GLM-sofa | 7.391 (6.71, 8.07) | 5.864 (5.19, 6.54) |
|  | GLM-saps | 7.464 (6.78, 8.15) | 6.056 (5.38, 6.73) |
|  | TMLE-dmg | 6.251 (5.98, 6.52) | 5.016 (4.94, 5.09) |
|  | TMLE-dmg_saps | 6.176 (5.91, 6.44) | 5.016 (4.94, 5.09) |
| n_HR | [prm]TMLE-dmg_sofa | 31.293 (30.74, 31.84) | 31.16 (31.02, 31.3) |
|  | GLM | 30.913 (29.62, 32.21) | 30.936 (29.64, 32.24) |
|  | GLM-sofa | 27.692 (26.42, 28.97) | 27.383 (26.11, 28.65) |
|  | GLM-saps | 26.586 (25.29, 27.89) | 25.604 (24.33, 26.88) |
|  | TMLE-dmg | 31.136 (30.58, 31.7) | 31.16 (31.02, 31.3) |
|  | TMLE-dmg_saps | 30.977 (30.44, 31.52) | 31.161 (31.02, 31.3) |
| n_SpO2 | [prm]TMLE-dmg_sofa | 30.906 (30.34, 31.47) | 30.625 (30.49, 30.76) |
|  | GLM | 30.788 (29.49, 32.09) | 30.456 (29.15, 31.76) |
|  | GLM-sofa | 27.828 (26.54, 29.11) | 27.191 (25.91, 28.47) |
|  | GLM-saps | 27.045 (25.73, 28.36) | 25.843 (24.55, 27.13) |
|  | TMLE-dmg | 30.84 (30.26, 31.42) | 30.625 (30.49, 30.76) |
|  | TMLE-dmg_saps | 30.689 (30.13, 31.25) | 30.626 (30.49, 30.77) |
| n_RR | [prm]TMLE-dmg_sofa | 35.671 (35.06, 36.28) | 34.695 (34.53, 34.86) |
|  | GLM | 35.408 (33.88, 36.94) | 34.44 (32.9, 35.98) |
|  | GLM-sofa | 30.417 (28.94, 31.89) | 28.934 (27.46, 30.4) |
|  | GLM-saps | 28.466 (26.96, 29.98) | 25.886 (24.4, 27.37) |
|  | TMLE-dmg | 35.466 (34.84, 36.09) | 34.695 (34.53, 34.86) |
|  | TMLE-dmg_saps | 35.184 (34.6, 35.77) | 34.697 (34.53, 34.86) |
| n_TempC | [prm]TMLE-dmg_sofa | 11.934 (11.57, 12.29) | 11.894 (11.75, 12.04) |
|  | GLM | 10.146 (8.81, 11.48) | 11.895 (10.55, 13.24) |
|  | GLM-sofa | 7.935 (6.6, 9.27) | 9.457 (8.13, 10.79) |
|  | GLM-saps | 9.288 (7.92, 10.66) | 10.839 (9.49, 12.18) |
|  | TMLE-dmg | 11.877 (11.5, 12.25) | 11.896 (11.75, 12.04) |
|  | TMLE-dmg_saps | 11.49 (11.13, 11.85) | 11.896 (11.75, 12.04) |

####

#### Appendix G.4.2. Estimated Average Missing Data Rates for Vital Signs across Insurance Groups

| **Y** | **method** | **No Insurance** | **Insurance** |
| --- | --- | --- | --- |
| h_m_BP | [prm]TMLE-dmg_sofa | 2.535 (2.34, 2.73) | 2.331 (2.29, 2.37) |
|  | GLM | 2.427 (2.08, 2.78) | 2.288 (1.94, 2.64) |
|  | GLM-sofa | 2.708 (2.36, 3.06) | 2.598 (2.25, 2.95) |
|  | GLM-saps | 3.011 (2.66, 3.37) | 3.008 (2.66, 3.36) |
|  | TMLE-dmg | 2.545 (2.35, 2.74) | 2.331 (2.29, 2.37) |
|  | TMLE-dmg_saps | 2.53 (2.34, 2.72) | 2.331 (2.29, 2.37) |
| h_m_BPm | [prm]TMLE-dmg_sofa | 2.554 (2.36, 2.75) | 2.413 (2.37, 2.45) |
|  | GLM | 2.48 (2.12, 2.84) | 2.37 (2.01, 2.73) |
|  | GLM-sofa | 2.785 (2.42, 3.15) | 2.707 (2.35, 3.07) |
|  | GLM-saps | 3.108 (2.74, 3.47) | 3.144 (2.78, 3.5) |
|  | TMLE-dmg | 2.545 (2.35, 2.74) | 2.413 (2.37, 2.45) |
|  | TMLE-dmg_saps | 2.549 (2.36, 2.74) | 2.413 (2.37, 2.45) |
| h_m_GCS | [prm]TMLE-dmg_sofa | 21.486 (21.22, 21.75) | 21.081 (21, 21.17) |
|  | GLM | 21.203 (20.41, 22) | 20.977 (20.18, 21.78) |
|  | GLM-sofa | 20.951 (20.15, 21.76) | 20.698 (19.9, 21.5) |
|  | GLM-saps | 21.061 (20.24, 21.88) | 20.801 (20, 21.6) |
|  | TMLE-dmg | 21.494 (21.22, 21.77) | 21.082 (21, 21.17) |
|  | TMLE-dmg_saps | 21.439 (21.17, 21.71) | 21.082 (21, 21.17) |
| h_m_GCS_Total | [prm]TMLE-dmg_sofa | 18.033 (17.75, 18.32) | 19.155 (19.08, 19.23) |
|  | GLM | 17.537 (16.83, 18.24) | 19.068 (18.36, 19.77) |
|  | GLM-sofa | 16.813 (16.11, 17.52) | 18.27 (17.57, 18.97) |
|  | GLM-saps | 16.717 (16, 17.43) | 18.059 (17.35, 18.76) |
|  | TMLE-dmg | 18.021 (17.73, 18.31) | 19.155 (19.08, 19.23) |
|  | TMLE-dmg_saps | 18.085 (17.81, 18.36) | 19.155 (19.08, 19.23) |
| h_m_HR | [prm]TMLE-dmg_sofa | 2.348 (2.15, 2.54) | 2.098 (2.06, 2.13) |
|  | GLM | 2.257 (1.92, 2.6) | 2.058 (1.72, 2.4) |
|  | GLM-sofa | 2.439 (2.1, 2.78) | 2.258 (1.92, 2.6) |
|  | GLM-saps | 2.73 (2.38, 3.08) | 2.64 (2.3, 2.98) |
|  | TMLE-dmg | 2.349 (2.15, 2.54) | 2.098 (2.06, 2.13) |
|  | TMLE-dmg_saps | 2.328 (2.14, 2.52) | 2.099 (2.06, 2.14) |
| h_m_SpO2 | [prm]TMLE-dmg_sofa | 2.878 (2.68, 3.08) | 2.713 (2.67, 2.76) |
|  | GLM | 2.705 (2.29, 3.12) | 2.657 (2.24, 3.07) |
|  | GLM-sofa | 2.859 (2.44, 3.27) | 2.827 (2.41, 3.24) |
|  | GLM-saps | 3.03 (2.61, 3.45) | 3.057 (2.64, 3.47) |
|  | TMLE-dmg | 2.881 (2.68, 3.08) | 2.713 (2.67, 2.76) |
|  | TMLE-dmg_saps | 2.853 (2.66, 3.05) | 2.713 (2.67, 2.76) |
| h_m_RR | [prm]TMLE-dmg_sofa | 2.639 (2.43, 2.85) | 2.411 (2.37, 2.45) |
|  | GLM | 2.558 (2.15, 2.97) | 2.364 (1.95, 2.78) |
|  | GLM-sofa | 2.73 (2.32, 3.14) | 2.554 (2.14, 2.97) |
|  | GLM-saps | 2.976 (2.56, 3.4) | 2.879 (2.47, 3.29) |
|  | TMLE-dmg | 2.62 (2.41, 2.83) | 2.411 (2.37, 2.45) |
|  | TMLE-dmg_saps | 2.6 (2.39, 2.81) | 2.411 (2.37, 2.45) |
| h_m_TempC | [prm]TMLE-dmg_sofa | 14.949 (14.7, 15.2) | 14.83 (14.74, 14.92) |
|  | GLM | 15.538 (14.69, 16.38) | 14.726 (13.88, 15.57) |
|  | GLM-sofa | 16.821 (15.98, 17.66) | 16.142 (15.3, 16.98) |
|  | GLM-saps | 16.073 (15.21, 16.94) | 15.386 (14.54, 16.23) |
|  | TMLE-dmg | 15.029 (14.75, 15.31) | 14.829 (14.74, 14.92) |
|  | TMLE-dmg_saps | 15.171 (14.91, 15.43) | 14.83 (14.74, 14.92) |

####

#### Appendix G.4.3. Estimated Average Measurement Frequencies for Lab Tests across Insurance Groups

| **Y** | **method** | **No Insurance** | **Insurance** |
| --- | --- | --- | --- |
| n_HCT | [prm]TMLE-dmg_sofa | 2.598 (2.53, 2.67) | 2.732 (2.71, 2.76) |
|  | GLM | 2.684 (2.46, 2.9) | 2.718 (2.5, 2.94) |
|  | GLM-sofa | 2.074 (1.86, 2.29) | 2.044 (1.83, 2.26) |
|  | GLM-saps | 2.204 (1.98, 2.43) | 2.126 (1.91, 2.35) |
|  | TMLE-dmg | 2.624 (2.55, 2.7) | 2.732 (2.71, 2.76) |
|  | TMLE-dmg_saps | 2.562 (2.49, 2.63) | 2.732 (2.71, 2.76) |
| n_Lac | [prm]TMLE-dmg_sofa | 1.858 (1.77, 1.94) | 1.582 (1.55, 1.61) |
|  | GLM | 1.877 (1.6, 2.15) | 1.554 (1.28, 1.83) |
|  | GLM-sofa | 0.572 (0.32, 0.82) | 0.114 (-0.14, 0.36) |
|  | GLM-saps | 0.394 (0.13, 0.66) | -0.274 (-0.54, -0.01) |
|  | TMLE-dmg | 1.82 (1.72, 1.92) | 1.583 (1.55, 1.61) |
|  | TMLE-dmg_saps | 1.758 (1.68, 1.84) | 1.583 (1.55, 1.61) |
| n_lab_grp1 | [prm]TMLE-dmg_sofa | 0.592 (0.55, 0.63) | 0.49 (0.48, 0.5) |
|  | GLM | 0.666 (0.56, 0.77) | 0.48 (0.38, 0.58) |
|  | GLM-sofa | 0.277 (0.18, 0.37) | 0.051 (-0.04, 0.15) |
|  | GLM-saps | 0.297 (0.2, 0.4) | 0.026 (-0.07, 0.13) |
|  | TMLE-dmg | 0.595 (0.55, 0.64) | 0.49 (0.48, 0.5) |
|  | TMLE-dmg_saps | 0.567 (0.53, 0.61) | 0.49 (0.48, 0.5) |
| n_lab_grp2 | [prm]TMLE-dmg_sofa | 4.138 (4, 4.27) | 4.334 (4.28, 4.39) |
|  | GLM | 3.893 (3.41, 4.37) | 4.31 (3.83, 4.79) |
|  | GLM-sofa | 1.754 (1.31, 2.2) | 1.951 (1.51, 2.39) |
|  | GLM-saps | 1.985 (1.51, 2.46) | 1.959 (1.49, 2.43) |
|  | TMLE-dmg | 4.062 (3.9, 4.22) | 4.335 (4.28, 4.39) |
|  | TMLE-dmg_saps | 4.006 (3.86, 4.15) | 4.335 (4.28, 4.39) |
| n_lab_grp3 | [prm]TMLE-dmg_sofa | 2.172 (2.12, 2.23) | 1.985 (1.97, 2) |
|  | GLM | 2.277 (2.14, 2.41) | 1.97 (1.84, 2.1) |
|  | GLM-sofa | 1.811 (1.68, 1.94) | 1.456 (1.33, 1.58) |
|  | GLM-saps | 1.684 (1.55, 1.82) | 1.24 (1.11, 1.37) |
|  | TMLE-dmg | 2.16 (2.1, 2.22) | 1.985 (1.97, 2) |
|  | TMLE-dmg_saps | 2.143 (2.09, 2.2) | 1.985 (1.97, 2) |
| n_lab_grp4 | [prm]TMLE-dmg_sofa | 1.904 (1.85, 1.95) | 1.994 (1.98, 2.01) |
|  | GLM | 1.961 (1.82, 2.1) | 1.984 (1.84, 2.12) |
|  | GLM-sofa | 1.446 (1.31, 1.58) | 1.415 (1.28, 1.55) |
|  | GLM-saps | 1.494 (1.35, 1.64) | 1.408 (1.27, 1.55) |
|  | TMLE-dmg | 1.907 (1.85, 1.96) | 1.994 (1.98, 2.01) |
|  | TMLE-dmg_saps | 1.87 (1.82, 1.92) | 1.994 (1.98, 2.01) |
| n_lab_grp5 | [prm]TMLE-dmg_sofa | 0.319 (0.3, 0.34) | 0.289 (0.28, 0.3) |
|  | GLM | 0.331 (0.26, 0.4) | 0.283 (0.22, 0.35) |
|  | GLM-sofa | 0.189 (0.12, 0.26) | 0.127 (0.06, 0.19) |
|  | GLM-saps | 0.129 (0.06, 0.2) | 0.035 (-0.03, 0.1) |
|  | TMLE-dmg | 0.327 (0.3, 0.35) | 0.289 (0.28, 0.3) |
|  | TMLE-dmg_saps | 0.309 (0.29, 0.33) | 0.289 (0.28, 0.3) |
| n_lab_grp6 | [prm]TMLE-dmg_sofa | 1.808 (1.76, 1.86) | 1.6 (1.58, 1.62) |
|  | GLM | 1.976 (1.83, 2.12) | 1.585 (1.44, 1.73) |
|  | GLM-sofa | 1.568 (1.43, 1.71) | 1.136 (1, 1.27) |
|  | GLM-saps | 1.414 (1.27, 1.56) | 0.894 (0.75, 1.03) |
|  | TMLE-dmg | 1.795 (1.74, 1.85) | 1.6 (1.58, 1.62) |
|  | TMLE-dmg_saps | 1.782 (1.73, 1.83) | 1.6 (1.58, 1.62) |
| n_lab_grp7 | [prm]TMLE-dmg_sofa | 1.691 (1.63, 1.75) | 1.723 (1.7, 1.74) |
|  | GLM | 1.726 (1.55, 1.9) | 1.712 (1.54, 1.88) |
|  | GLM-sofa | 1.13 (0.97, 1.29) | 1.056 (0.89, 1.22) |
|  | GLM-saps | 1.192 (1.02, 1.36) | 1.055 (0.89, 1.22) |
|  | TMLE-dmg | 1.706 (1.64, 1.77) | 1.723 (1.7, 1.74) |
|  | TMLE-dmg_saps | 1.669 (1.61, 1.73) | 1.723 (1.7, 1.74) |

#### Appendix G.5.1. Estimated Average Measurement Frequencies for Vital Signs across Language Groups

| **Y** | **method** | **ENGL** | **Other** | **Missing** |
| --- | --- | --- | --- | --- |
| n_BP | [prm]TMLE-dmg_sofa | 33.715 (33.48, 33.95) | 34.003 (33.3, 34.7) | 35.01 (34.7, 35.32) |
|  | GLM | 33.424 (33.18, 33.67) | 33.804 (33.14, 34.47) | 34.775 (34.4, 35.15) |
|  | GLM-sofa | 28.608 (28.25, 28.96) | 28.648 (28, 29.3) | 30.012 (29.65, 30.38) |
|  | GLM-saps | 26.097 (25.59, 26.61) | 25.995 (25.34, 26.65) | 27.395 (27.03, 27.76) |
|  | TMLE-dmg | 33.706 (33.47, 33.94) | 34.354 (33.62, 35.09) | 34.928 (34.61, 35.25) |
|  | TMLE-dmg_saps | 33.703 (33.47, 33.93) | 34.032 (33.33, 34.74) | 34.998 (34.68, 35.31) |
| n_BPm | [prm]TMLE-dmg_sofa | 33.605 (33.37, 33.84) | 33.78 (33.08, 34.48) | 34.747 (34.44, 35.05) |
|  | GLM | 33.309 (33.06, 33.56) | 33.627 (32.96, 34.29) | 34.502 (34.13, 34.87) |
|  | GLM-sofa | 28.369 (28.02, 28.72) | 28.339 (27.69, 28.98) | 29.617 (29.26, 29.98) |
|  | GLM-saps | 25.752 (25.25, 26.26) | 25.574 (24.92, 26.22) | 26.891 (26.53, 27.25) |
|  | TMLE-dmg | 33.597 (33.36, 33.83) | 34.135 (33.4, 34.87) | 34.661 (34.35, 34.98) |
|  | TMLE-dmg_saps | 33.593 (33.36, 33.83) | 33.806 (33.1, 34.51) | 34.737 (34.43, 35.05) |
| n_GCS | [prm]TMLE-dmg_sofa | 5.489 (5.39, 5.59) | 3.175 (2.89, 3.46) | 0.056 (0.04, 0.07) |
|  | GLM | 5.482 (5.41, 5.56) | 3.41 (3.21, 3.61) | 0.037 (-0.08, 0.15) |
|  | GLM-sofa | 5.896 (5.78, 6.01) | 3.853 (3.65, 4.06) | 0.446 (0.33, 0.56) |
|  | GLM-saps | 5.683 (5.52, 5.84) | 3.623 (3.42, 3.83) | 0.239 (0.12, 0.35) |
|  | TMLE-dmg | 5.501 (5.4, 5.6) | 3.198 (2.92, 3.48) | 0.056 (0.04, 0.07) |
|  | TMLE-dmg_saps | 5.498 (5.4, 5.6) | 3.197 (2.91, 3.48) | 0.057 (0.04, 0.07) |
| n_GCS_Total | [prm]TMLE-dmg_sofa | 2.633 (2.55, 2.71) | 4.837 (4.55, 5.12) | 8.126 (8.02, 8.23) |
|  | GLM | 2.613 (2.53, 2.7) | 4.687 (4.45, 4.92) | 8.076 (7.94, 8.21) |
|  | GLM-sofa | 3.46 (3.33, 3.59) | 5.593 (5.36, 5.83) | 8.913 (8.78, 9.04) |
|  | GLM-saps | 3.743 (3.56, 3.92) | 5.891 (5.66, 6.12) | 9.214 (9.08, 9.34) |
|  | TMLE-dmg | 2.629 (2.55, 2.71) | 4.756 (4.48, 5.04) | 8.129 (8.02, 8.24) |
|  | TMLE-dmg_saps | 2.631 (2.55, 2.71) | 4.819 (4.53, 5.1) | 8.126 (8.02, 8.23) |
| n_HR | [prm]TMLE-dmg_sofa | 30.416 (30.25, 30.58) | 30.788 (30.3, 31.28) | 32.138 (31.89, 32.38) |
|  | GLM | 30.168 (29.98, 30.36) | 30.741 (30.24, 31.25) | 31.942 (31.66, 32.22) |
|  | GLM-sofa | 26.608 (26.34, 26.88) | 26.931 (26.44, 27.42) | 28.422 (28.15, 28.7) |
|  | GLM-saps | 24.906 (24.52, 25.29) | 25.134 (24.64, 25.63) | 26.642 (26.36, 26.92) |
|  | TMLE-dmg | 30.41 (30.25, 30.58) | 31.015 (30.5, 31.53) | 32.065 (31.81, 32.32) |
|  | TMLE-dmg_saps | 30.408 (30.24, 30.57) | 30.777 (30.29, 31.27) | 32.124 (31.88, 32.37) |
| n_SpO2 | [prm]TMLE-dmg_sofa | 29.929 (29.77, 30.09) | 30.186 (29.7, 30.67) | 31.55 (31.3, 31.8) |
|  | GLM | 29.735 (29.55, 29.92) | 30.192 (29.69, 30.7) | 31.427 (31.14, 31.71) |
|  | GLM-sofa | 26.462 (26.19, 26.73) | 26.689 (26.19, 27.19) | 28.191 (27.91, 28.47) |
|  | GLM-saps | 25.185 (24.8, 25.58) | 25.344 (24.84, 25.84) | 26.845 (26.56, 27.13) |
|  | TMLE-dmg | 29.92 (29.76, 30.08) | 30.368 (29.87, 30.87) | 31.496 (31.24, 31.75) |
|  | TMLE-dmg_saps | 29.914 (29.75, 30.08) | 30.19 (29.71, 30.67) | 31.552 (31.3, 31.8) |
| n_RR | [prm]TMLE-dmg_sofa | 35.36 (35.16, 35.56) | 34.35 (33.76, 34.94) | 33.864 (33.59, 34.14) |
|  | GLM | 35.086 (34.86, 35.31) | 34.649 (34.05, 35.25) | 33.609 (33.28, 33.94) |
|  | GLM-sofa | 29.602 (29.29, 29.91) | 28.778 (28.21, 29.35) | 28.185 (27.87, 28.5) |
|  | GLM-saps | 26.629 (26.18, 27.08) | 25.635 (25.06, 26.21) | 25.09 (24.77, 25.41) |
|  | TMLE-dmg | 35.356 (35.15, 35.56) | 34.739 (34.11, 35.37) | 33.767 (33.48, 34.05) |
|  | TMLE-dmg_saps | 35.354 (35.15, 35.56) | 34.283 (33.69, 34.87) | 33.853 (33.57, 34.14) |
| n_TempC | [prm]TMLE-dmg_sofa | 10.508 (10.34, 10.67) | 11.638 (11.07, 12.2) | 13.603 (13.35, 13.85) |
|  | GLM | 10.448 (10.25, 10.64) | 11.498 (10.98, 12.02) | 13.753 (13.46, 14.04) |
|  | GLM-sofa | 7.986 (7.71, 8.26) | 8.863 (8.35, 9.37) | 11.319 (11.03, 11.61) |
|  | GLM-saps | 9.401 (9, 9.8) | 10.383 (9.87, 10.9) | 12.7 (12.41, 12.99) |
|  | TMLE-dmg | 10.483 (10.32, 10.65) | 11.746 (11.17, 12.32) | 13.596 (13.34, 13.85) |
|  | TMLE-dmg_saps | 10.479 (10.31, 10.64) | 11.716 (11.14, 12.29) | 13.621 (13.37, 13.87) |

####

#### Appendix G.5.2. Estimated Average Missing Data Rates for Vital Signs across Language Groups

| **Y** | **method** | **ENGL** | **Other** | **Missing** |
| --- | --- | --- | --- | --- |
| h_m_BP | [prm]TMLE-dmg_sofa | 2.222 (2.17, 2.27) | 2.215 (2.06, 2.37) | 2.482 (2.42, 2.55) |
|  | GLM | 2.188 (2.14, 2.24) | 2.118 (1.98, 2.25) | 2.453 (2.38, 2.53) |
|  | GLM-sofa | 2.494 (2.42, 2.57) | 2.446 (2.31, 2.58) | 2.756 (2.68, 2.83) |
|  | GLM-saps | 2.899 (2.79, 3) | 2.876 (2.74, 3.01) | 3.17 (3.09, 3.25) |
|  | TMLE-dmg | 2.224 (2.18, 2.27) | 2.185 (2.04, 2.33) | 2.492 (2.43, 2.56) |
|  | TMLE-dmg_saps | 2.223 (2.18, 2.27) | 2.207 (2.06, 2.36) | 2.485 (2.42, 2.55) |
| h_m_BPm | [prm]TMLE-dmg_sofa | 2.267 (2.22, 2.32) | 2.313 (2.15, 2.47) | 2.604 (2.54, 2.67) |
|  | GLM | 2.235 (2.18, 2.29) | 2.205 (2.06, 2.35) | 2.577 (2.5, 2.66) |
|  | GLM-sofa | 2.567 (2.49, 2.64) | 2.56 (2.42, 2.7) | 2.906 (2.83, 2.98) |
|  | GLM-saps | 3 (2.89, 3.11) | 3.02 (2.88, 3.16) | 3.348 (3.27, 3.43) |
|  | TMLE-dmg | 2.269 (2.22, 2.32) | 2.28 (2.13, 2.43) | 2.615 (2.55, 2.68) |
|  | TMLE-dmg_saps | 2.268 (2.22, 2.32) | 2.304 (2.15, 2.46) | 2.607 (2.54, 2.67) |
| h_m_GCS | [prm]TMLE-dmg_sofa | 18.73 (18.63, 18.83) | 20.972 (20.7, 21.25) | 24.109 (23.99, 24.23) |
|  | GLM | 18.622 (18.52, 18.73) | 20.632 (20.34, 20.92) | 24.019 (23.86, 24.18) |
|  | GLM-sofa | 18.309 (18.15, 18.47) | 20.297 (20.01, 20.58) | 23.71 (23.55, 23.87) |
|  | GLM-saps | 18.498 (18.27, 18.72) | 20.499 (20.21, 20.79) | 23.894 (23.73, 24.05) |
|  | TMLE-dmg | 18.717 (18.62, 18.81) | 20.949 (20.68, 21.22) | 24.112 (23.99, 24.24) |
|  | TMLE-dmg_saps | 18.719 (18.62, 18.82) | 20.95 (20.67, 21.23) | 24.114 (23.99, 24.24) |
| h_m_GCS_Total | [prm]TMLE-dmg_sofa | 21.472 (21.39, 21.56) | 19.267 (18.99, 19.55) | 16.13 (16.02, 16.24) |
|  | GLM | 21.379 (21.29, 21.47) | 19.299 (19.05, 19.55) | 16.07 (15.93, 16.21) |
|  | GLM-sofa | 20.612 (20.48, 20.75) | 18.478 (18.23, 18.72) | 15.311 (15.17, 15.45) |
|  | GLM-saps | 20.311 (20.12, 20.5) | 18.161 (17.91, 18.41) | 14.994 (14.86, 15.13) |
|  | TMLE-dmg | 21.475 (21.39, 21.56) | 19.332 (19.06, 19.61) | 16.129 (16.01, 16.25) |
|  | TMLE-dmg_saps | 21.472 (21.39, 21.56) | 19.276 (19, 19.56) | 16.132 (16.02, 16.25) |
| h_m_HR | [prm]TMLE-dmg_sofa | 1.929 (1.88, 1.97) | 1.978 (1.83, 2.13) | 2.33 (2.27, 2.39) |
|  | GLM | 1.891 (1.84, 1.94) | 1.901 (1.77, 2.03) | 2.305 (2.23, 2.38) |
|  | GLM-sofa | 2.087 (2.02, 2.16) | 2.111 (1.98, 2.24) | 2.499 (2.42, 2.57) |
|  | GLM-saps | 2.469 (2.37, 2.57) | 2.517 (2.38, 2.65) | 2.887 (2.81, 2.96) |
|  | TMLE-dmg | 1.929 (1.88, 1.97) | 1.951 (1.81, 2.09) | 2.341 (2.28, 2.41) |
|  | TMLE-dmg_saps | 1.929 (1.88, 1.97) | 1.971 (1.82, 2.12) | 2.335 (2.27, 2.4) |
| h_m_SpO2 | [prm]TMLE-dmg_sofa | 2.351 (2.3, 2.41) | 2.569 (2.39, 2.74) | 3.197 (3.12, 3.27) |
|  | GLM | 2.297 (2.24, 2.36) | 2.458 (2.3, 2.62) | 3.153 (3.06, 3.24) |
|  | GLM-sofa | 2.459 (2.37, 2.55) | 2.632 (2.47, 2.79) | 3.314 (3.22, 3.4) |
|  | GLM-saps | 2.695 (2.57, 2.82) | 2.883 (2.72, 3.04) | 3.554 (3.47, 3.64) |
|  | TMLE-dmg | 2.352 (2.3, 2.41) | 2.545 (2.37, 2.72) | 3.2 (3.12, 3.28) |
|  | TMLE-dmg_saps | 2.353 (2.3, 2.41) | 2.552 (2.38, 2.73) | 3.194 (3.12, 3.27) |
| h_m_RR | [prm]TMLE-dmg_sofa | 2.057 (2.01, 2.11) | 2.284 (2.1, 2.47) | 2.89 (2.81, 2.97) |
|  | GLM | 2.013 (1.95, 2.07) | 2.161 (2, 2.32) | 2.854 (2.76, 2.94) |
|  | GLM-sofa | 2.195 (2.11, 2.28) | 2.355 (2.2, 2.51) | 3.033 (2.94, 3.12) |
|  | GLM-saps | 2.526 (2.4, 2.65) | 2.708 (2.55, 2.87) | 3.371 (3.28, 3.46) |
|  | TMLE-dmg | 2.058 (2.01, 2.11) | 2.255 (2.08, 2.43) | 2.9 (2.82, 2.98) |
|  | TMLE-dmg_saps | 2.057 (2.01, 2.11) | 2.287 (2.1, 2.47) | 2.893 (2.81, 2.97) |
| h_m_TempC | [prm]TMLE-dmg_sofa | 15.417 (15.32, 15.52) | 14.788 (14.47, 15.11) | 14.117 (13.96, 14.27) |
|  | GLM | 15.343 (15.22, 15.47) | 14.722 (14.39, 15.05) | 13.971 (13.79, 14.15) |
|  | GLM-sofa | 16.766 (16.59, 16.94) | 16.245 (15.92, 16.57) | 15.378 (15.2, 15.56) |
|  | GLM-saps | 15.992 (15.74, 16.25) | 15.413 (15.08, 15.74) | 14.624 (14.44, 14.81) |
|  | TMLE-dmg | 15.427 (15.33, 15.53) | 14.715 (14.39, 15.04) | 14.134 (13.97, 14.3) |
|  | TMLE-dmg_saps | 15.427 (15.33, 15.53) | 14.738 (14.42, 15.06) | 14.12 (13.96, 14.28) |

####

#### Appendix G.5.3. Estimated Average Measurement Frequencies for Lab Tests across Language Groups

| **Y** | **method** | **ENGL** | **Other** | **Missing** |
| --- | --- | --- | --- | --- |
| n_HCT | [prm]TMLE-dmg_sofa | 2.805 (2.78, 2.83) | 2.731 (2.64, 2.82) | 2.633 (2.59, 2.67) |
|  | GLM | 2.792 (2.76, 2.82) | 2.734 (2.65, 2.82) | 2.62 (2.57, 2.67) |
|  | GLM-sofa | 2.121 (2.08, 2.17) | 2.016 (1.93, 2.1) | 1.956 (1.91, 2) |
|  | GLM-saps | 2.205 (2.14, 2.27) | 2.109 (2.02, 2.19) | 2.029 (1.98, 2.08) |
|  | TMLE-dmg | 2.804 (2.77, 2.83) | 2.771 (2.68, 2.86) | 2.624 (2.58, 2.66) |
|  | TMLE-dmg_saps | 2.803 (2.77, 2.83) | 2.744 (2.65, 2.83) | 2.631 (2.59, 2.67) |
| n_Lac | [prm]TMLE-dmg_sofa | 1.854 (1.82, 1.89) | 1.675 (1.56, 1.79) | 1.239 (1.2, 1.28) |
|  | GLM | 1.828 (1.79, 1.87) | 1.754 (1.65, 1.86) | 1.176 (1.12, 1.24) |
|  | GLM-sofa | 0.398 (0.35, 0.45) | 0.223 (0.13, 0.32) | -0.238 (-0.29, -0.18) |
|  | GLM-saps | 0.024 (-0.05, 0.1) | -0.168 (-0.27, -0.07) | -0.64 (-0.7, -0.58) |
|  | TMLE-dmg | 1.856 (1.81, 1.9) | 1.771 (1.64, 1.91) | 1.217 (1.17, 1.26) |
|  | TMLE-dmg_saps | 1.856 (1.82, 1.9) | 1.66 (1.54, 1.78) | 1.234 (1.19, 1.28) |
| n_lab_grp1 | [prm]TMLE-dmg_sofa | 0.515 (0.5, 0.53) | 0.557 (0.51, 0.6) | 0.453 (0.44, 0.47) |
|  | GLM | 0.513 (0.5, 0.53) | 0.559 (0.52, 0.6) | 0.428 (0.41, 0.45) |
|  | GLM-sofa | 0.086 (0.07, 0.11) | 0.102 (0.06, 0.14) | 0.005 (-0.02, 0.03) |
|  | GLM-saps | 0.068 (0.04, 0.1) | 0.084 (0.05, 0.12) | -0.021 (-0.04, 0) |
|  | TMLE-dmg | 0.515 (0.5, 0.53) | 0.589 (0.54, 0.64) | 0.446 (0.43, 0.46) |
|  | TMLE-dmg_saps | 0.515 (0.5, 0.53) | 0.553 (0.51, 0.6) | 0.449 (0.43, 0.47) |
| n_lab_grp2 | [prm]TMLE-dmg_sofa | 4.021 (3.96, 4.08) | 4.139 (3.95, 4.33) | 4.713 (4.63, 4.79) |
|  | GLM | 3.992 (3.92, 4.06) | 4.233 (4.05, 4.42) | 4.714 (4.61, 4.82) |
|  | GLM-sofa | 1.631 (1.54, 1.72) | 1.706 (1.53, 1.88) | 2.379 (2.28, 2.47) |
|  | GLM-saps | 1.666 (1.52, 1.81) | 1.755 (1.57, 1.94) | 2.372 (2.27, 2.47) |
|  | TMLE-dmg | 4.018 (3.95, 4.08) | 4.264 (4.05, 4.48) | 4.693 (4.61, 4.78) |
|  | TMLE-dmg_saps | 4.014 (3.95, 4.08) | 4.156 (3.96, 4.36) | 4.719 (4.64, 4.8) |
| n_lab_grp3 | [prm]TMLE-dmg_sofa | 2.071 (2.05, 2.09) | 1.992 (1.94, 2.05) | 1.887 (1.86, 1.91) |
|  | GLM | 2.061 (2.04, 2.08) | 2.011 (1.96, 2.06) | 1.856 (1.83, 1.89) |
|  | GLM-sofa | 1.55 (1.52, 1.58) | 1.464 (1.41, 1.51) | 1.351 (1.32, 1.38) |
|  | GLM-saps | 1.342 (1.3, 1.38) | 1.244 (1.19, 1.29) | 1.131 (1.1, 1.16) |
|  | TMLE-dmg | 2.07 (2.05, 2.09) | 2.026 (1.97, 2.08) | 1.88 (1.86, 1.9) |
|  | TMLE-dmg_saps | 2.07 (2.05, 2.09) | 1.981 (1.93, 2.04) | 1.886 (1.86, 1.91) |
| n_lab_grp4 | [prm]TMLE-dmg_sofa | 2.126 (2.11, 2.15) | 2.001 (1.94, 2.06) | 1.823 (1.8, 1.85) |
|  | GLM | 2.118 (2.1, 2.14) | 2.017 (1.96, 2.07) | 1.807 (1.78, 1.84) |
|  | GLM-sofa | 1.552 (1.52, 1.58) | 1.411 (1.36, 1.46) | 1.248 (1.22, 1.28) |
|  | GLM-saps | 1.546 (1.5, 1.59) | 1.407 (1.35, 1.46) | 1.231 (1.2, 1.26) |
|  | TMLE-dmg | 2.125 (2.1, 2.15) | 2.032 (1.97, 2.09) | 1.816 (1.79, 1.84) |
|  | TMLE-dmg_saps | 2.125 (2.1, 2.14) | 2 (1.94, 2.06) | 1.822 (1.8, 1.84) |
| n_lab_grp5 | [prm]TMLE-dmg_sofa | 0.32 (0.31, 0.33) | 0.334 (0.3, 0.37) | 0.242 (0.23, 0.25) |
|  | GLM | 0.315 (0.31, 0.33) | 0.33 (0.3, 0.36) | 0.235 (0.22, 0.25) |
|  | GLM-sofa | 0.16 (0.15, 0.17) | 0.164 (0.14, 0.19) | 0.081 (0.07, 0.1) |
|  | GLM-saps | 0.071 (0.05, 0.09) | 0.069 (0.04, 0.1) | -0.011 (-0.03, 0) |
|  | TMLE-dmg | 0.32 (0.31, 0.33) | 0.34 (0.31, 0.37) | 0.24 (0.23, 0.25) |
|  | TMLE-dmg_saps | 0.32 (0.31, 0.33) | 0.326 (0.3, 0.36) | 0.242 (0.23, 0.25) |
| n_lab_grp6 | [prm]TMLE-dmg_sofa | 1.653 (1.63, 1.67) | 1.636 (1.57, 1.7) | 1.539 (1.52, 1.56) |
|  | GLM | 1.645 (1.62, 1.67) | 1.641 (1.59, 1.7) | 1.508 (1.48, 1.54) |
|  | GLM-sofa | 1.199 (1.17, 1.23) | 1.164 (1.11, 1.22) | 1.067 (1.04, 1.1) |
|  | GLM-saps | 0.967 (0.92, 1.01) | 0.918 (0.86, 0.97) | 0.825 (0.79, 0.86) |
|  | TMLE-dmg | 1.652 (1.63, 1.67) | 1.666 (1.6, 1.73) | 1.532 (1.51, 1.56) |
|  | TMLE-dmg_saps | 1.653 (1.63, 1.67) | 1.626 (1.56, 1.69) | 1.538 (1.51, 1.56) |
| n_lab_grp7 | [prm]TMLE-dmg_sofa | 1.715 (1.69, 1.74) | 1.737 (1.67, 1.8) | 1.728 (1.7, 1.76) |
|  | GLM | 1.707 (1.68, 1.73) | 1.734 (1.67, 1.8) | 1.716 (1.68, 1.75) |
|  | GLM-sofa | 1.051 (1.02, 1.09) | 1.032 (0.97, 1.1) | 1.067 (1.03, 1.1) |
|  | GLM-saps | 1.058 (1.01, 1.11) | 1.042 (0.98, 1.11) | 1.062 (1.03, 1.1) |
|  | TMLE-dmg | 1.714 (1.69, 1.74) | 1.776 (1.7, 1.85) | 1.718 (1.69, 1.75) |
|  | TMLE-dmg_saps | 1.714 (1.69, 1.74) | 1.745 (1.67, 1.82) | 1.726 (1.7, 1.76) |

####

#### Appendix G.6.1. Estimated Average Measurement Frequencies for Vital Signs across Partner Groups

| **Y** | **method** | **With partner** | **No partner** | **Missing** |
| --- | --- | --- | --- | --- |
| n_BP | [prm]TMLE-dmg_sofa | 34.711 (34.47, 34.95) | 33.918 (33.56, 34.28) | 33.636 (32.99, 34.29) |
|  | GLM | 34.612 (34.24, 34.98) | 33.378 (33.11, 33.65) | 33.432 (32.7, 34.17) |
|  | GLM-sofa | 29.837 (29.48, 30.2) | 28.521 (28.15, 28.89) | 28.476 (27.76, 29.19) |
|  | GLM-saps | 27.244 (26.88, 27.61) | 25.96 (25.44, 26.48) | 25.691 (24.97, 26.41) |
|  | TMLE-dmg | 34.559 (34.32, 34.8) | 34.086 (33.72, 34.45) | 33.75 (33.07, 34.43) |
|  | TMLE-dmg_saps | 34.684 (34.45, 34.92) | 33.944 (33.59, 34.29) | 33.4 (32.76, 34.04) |
| n_BPm | [prm]TMLE-dmg_sofa | 34.523 (34.28, 34.76) | 33.74 (33.39, 34.09) | 33.51 (32.85, 34.17) |
|  | GLM | 34.405 (34.04, 34.77) | 33.211 (32.94, 33.48) | 33.306 (32.58, 34.04) |
|  | GLM-sofa | 29.507 (29.15, 29.86) | 28.229 (27.86, 28.6) | 28.222 (27.51, 28.93) |
|  | GLM-saps | 26.811 (26.45, 27.17) | 25.565 (25.04, 26.09) | 25.327 (24.61, 26.04) |
|  | TMLE-dmg | 34.373 (34.13, 34.62) | 33.909 (33.55, 34.26) | 33.624 (32.94, 34.31) |
|  | TMLE-dmg_saps | 34.498 (34.26, 34.74) | 33.768 (33.43, 34.11) | 33.275 (32.63, 33.92) |
| n_GCS | [prm]TMLE-dmg_sofa | 3.049 (2.96, 3.14) | 3.223 (3.13, 3.32) | 3.03 (2.74, 3.32) |
|  | GLM | 2.955 (2.82, 3.09) | 3.251 (3.15, 3.35) | 3.234 (2.97, 3.5) |
|  | GLM-sofa | 3.334 (3.2, 3.47) | 3.636 (3.5, 3.77) | 3.627 (3.37, 3.89) |
|  | GLM-saps | 3.21 (3.08, 3.34) | 3.507 (3.32, 3.7) | 3.501 (3.24, 3.76) |
|  | TMLE-dmg | 3.066 (2.97, 3.16) | 3.21 (3.11, 3.31) | 3.067 (2.78, 3.35) |
|  | TMLE-dmg_saps | 3.067 (2.97, 3.16) | 3.218 (3.12, 3.32) | 3.045 (2.76, 3.33) |
| n_GCS_Total | [prm]TMLE-dmg_sofa | 5.027 (4.93, 5.12) | 4.86 (4.72, 5) | 6.216 (5.91, 6.53) |
|  | GLM | 4.974 (4.83, 5.12) | 4.866 (4.76, 4.97) | 6.084 (5.79, 6.37) |
|  | GLM-sofa | 5.851 (5.71, 6) | 5.758 (5.61, 5.91) | 6.994 (6.71, 7.28) |
|  | GLM-saps | 6.079 (5.93, 6.22) | 5.978 (5.77, 6.19) | 7.245 (6.96, 7.53) |
|  | TMLE-dmg | 5.046 (4.95, 5.14) | 4.848 (4.71, 4.99) | 6.143 (5.83, 6.45) |
|  | TMLE-dmg_saps | 5.032 (4.94, 5.13) | 4.854 (4.72, 4.99) | 6.242 (5.93, 6.56) |
| n_HR | [prm]TMLE-dmg_sofa | 31.425 (31.25, 31.6) | 30.95 (30.66, 31.24) | 30.907 (30.41, 31.41) |
|  | GLM | 31.286 (31.01, 31.57) | 30.556 (30.35, 30.76) | 30.722 (30.17, 31.28) |
|  | GLM-sofa | 27.764 (27.49, 28.04) | 26.974 (26.69, 27.26) | 27.067 (26.52, 27.61) |
|  | GLM-saps | 25.994 (25.72, 26.27) | 25.228 (24.83, 25.63) | 25.162 (24.62, 25.71) |
|  | TMLE-dmg | 31.328 (31.15, 31.5) | 31.067 (30.77, 31.37) | 30.993 (30.48, 31.5) |
|  | TMLE-dmg_saps | 31.4 (31.22, 31.58) | 30.971 (30.69, 31.25) | 30.735 (30.24, 31.23) |
| n_SpO2 | [prm]TMLE-dmg_sofa | 30.864 (30.69, 31.04) | 30.413 (30.13, 30.69) | 30.498 (29.99, 31) |
|  | GLM | 30.802 (30.52, 31.08) | 30.048 (29.84, 30.26) | 30.519 (29.96, 31.08) |
|  | GLM-sofa | 27.566 (27.29, 27.84) | 26.757 (26.47, 27.04) | 27.16 (26.61, 27.71) |
|  | GLM-saps | 26.232 (25.95, 26.51) | 25.447 (25.05, 25.85) | 25.717 (25.17, 26.27) |
|  | TMLE-dmg | 30.766 (30.59, 30.94) | 30.528 (30.24, 30.82) | 30.548 (30.03, 31.07) |
|  | TMLE-dmg_saps | 30.827 (30.65, 31.01) | 30.436 (30.16, 30.71) | 30.375 (29.87, 30.88) |
| n_RR | [prm]TMLE-dmg_sofa | 34.818 (34.61, 35.02) | 34.538 (34.21, 34.86) | 35.18 (34.59, 35.77) |
|  | GLM | 34.576 (34.25, 34.91) | 34.162 (33.92, 34.41) | 35.346 (34.69, 36) |
|  | GLM-sofa | 29.131 (28.81, 29.45) | 28.624 (28.3, 28.95) | 29.695 (29.07, 30.32) |
|  | GLM-saps | 26.134 (25.81, 26.45) | 25.662 (25.2, 26.13) | 26.476 (25.84, 27.11) |
|  | TMLE-dmg | 34.657 (34.45, 34.86) | 34.721 (34.38, 35.06) | 35.372 (34.74, 36.01) |
|  | TMLE-dmg_saps | 34.806 (34.6, 35.01) | 34.554 (34.24, 34.87) | 34.895 (34.3, 35.49) |
| n_TempC | [prm]TMLE-dmg_sofa | 12.401 (12.2, 12.6) | 11.314 (11.1, 11.52) | 11.112 (10.62, 11.61) |
|  | GLM | 12.755 (12.47, 13.04) | 10.964 (10.75, 11.18) | 11.098 (10.52, 11.67) |
|  | GLM-sofa | 10.321 (10.04, 10.61) | 8.487 (8.19, 8.78) | 8.571 (8, 9.14) |
|  | GLM-saps | 11.65 (11.36, 11.94) | 9.851 (9.43, 10.27) | 9.937 (9.36, 10.51) |
|  | TMLE-dmg | 12.324 (12.13, 12.52) | 11.368 (11.16, 11.58) | 11.131 (10.62, 11.64) |
|  | TMLE-dmg_saps | 12.332 (12.13, 12.53) | 11.365 (11.15, 11.58) | 11.161 (10.66, 11.67) |

####

#### Appendix G.6.2. Estimated Average Missing Data Rates for Vital Signs across Partner Groups

| **Y** | **method** | **With partner** | **No partner** | **Missing** |
| --- | --- | --- | --- | --- |
| h_m_BP | [prm]TMLE-dmg_sofa | 2.342 (2.29, 2.39) | 2.28 (2.22, 2.34) | 2.505 (2.35, 2.66) |
|  | GLM | 2.322 (2.25, 2.4) | 2.229 (2.17, 2.28) | 2.433 (2.28, 2.58) |
|  | GLM-sofa | 2.629 (2.55, 2.7) | 2.541 (2.46, 2.62) | 2.751 (2.6, 2.9) |
|  | GLM-saps | 3.039 (2.96, 3.11) | 2.951 (2.84, 3.06) | 3.186 (3.04, 3.34) |
|  | TMLE-dmg | 2.352 (2.3, 2.4) | 2.276 (2.22, 2.34) | 2.521 (2.36, 2.68) |
|  | TMLE-dmg_saps | 2.337 (2.28, 2.39) | 2.287 (2.23, 2.35) | 2.544 (2.39, 2.7) |
| h_m_BPm | [prm]TMLE-dmg_sofa | 2.426 (2.37, 2.48) | 2.359 (2.3, 2.42) | 2.606 (2.45, 2.76) |
|  | GLM | 2.405 (2.33, 2.48) | 2.308 (2.25, 2.37) | 2.521 (2.37, 2.68) |
|  | GLM-sofa | 2.738 (2.66, 2.82) | 2.646 (2.57, 2.73) | 2.866 (2.71, 3.02) |
|  | GLM-saps | 3.175 (3.1, 3.25) | 3.083 (2.97, 3.2) | 3.33 (3.18, 3.48) |
|  | TMLE-dmg | 2.437 (2.38, 2.49) | 2.354 (2.29, 2.42) | 2.618 (2.45, 2.78) |
|  | TMLE-dmg_saps | 2.421 (2.37, 2.47) | 2.366 (2.3, 2.43) | 2.643 (2.48, 2.81) |
| h_m_GCS | [prm]TMLE-dmg_sofa | 21.106 (21.02, 21.2) | 21.062 (20.82, 21.3) | 21.29 (20.97, 21.61) |
|  | GLM | 21.077 (20.9, 21.25) | 20.872 (20.75, 21) | 20.925 (20.58, 21.27) |
|  | GLM-sofa | 20.8 (20.63, 20.97) | 20.59 (20.41, 20.77) | 20.637 (20.29, 20.98) |
|  | GLM-saps | 20.903 (20.73, 21.08) | 20.697 (20.45, 20.95) | 20.742 (20.4, 21.08) |
|  | TMLE-dmg | 21.09 (21, 21.18) | 21.088 (20.84, 21.33) | 21.284 (20.95, 21.62) |
|  | TMLE-dmg_saps | 21.099 (21.01, 21.19) | 21.067 (20.84, 21.29) | 21.304 (20.95, 21.65) |
| h_m_GCS_Total | [prm]TMLE-dmg_sofa | 19.106 (19.01, 19.2) | 19.387 (19.23, 19.55) | 18.08 (17.77, 18.39) |
|  | GLM | 19.045 (18.89, 19.2) | 19.22 (19.11, 19.33) | 18.036 (17.73, 18.34) |
|  | GLM-sofa | 18.249 (18.1, 18.4) | 18.41 (18.25, 18.57) | 17.21 (16.91, 17.51) |
|  | GLM-saps | 18.004 (17.85, 18.16) | 18.172 (17.95, 18.39) | 16.943 (16.64, 17.24) |
|  | TMLE-dmg | 19.081 (18.99, 19.18) | 19.416 (19.25, 19.58) | 18.165 (17.84, 18.49) |
|  | TMLE-dmg_saps | 19.097 (19, 19.19) | 19.395 (19.24, 19.55) | 18.092 (17.77, 18.42) |
| h_m_HR | [prm]TMLE-dmg_sofa | 2.135 (2.08, 2.19) | 2.018 (1.96, 2.08) | 2.302 (2.14, 2.46) |
|  | GLM | 2.122 (2.05, 2.2) | 1.957 (1.9, 2.01) | 2.245 (2.1, 2.39) |
|  | GLM-sofa | 2.321 (2.25, 2.39) | 2.159 (2.08, 2.23) | 2.451 (2.3, 2.6) |
|  | GLM-saps | 2.704 (2.63, 2.78) | 2.543 (2.44, 2.65) | 2.856 (2.71, 3) |
|  | TMLE-dmg | 2.141 (2.09, 2.19) | 2.017 (1.96, 2.07) | 2.32 (2.14, 2.5) |
|  | TMLE-dmg_saps | 2.129 (2.08, 2.18) | 2.026 (1.97, 2.08) | 2.339 (2.16, 2.52) |
| h_m_SpO2 | [prm]TMLE-dmg_sofa | 2.759 (2.7, 2.82) | 2.618 (2.55, 2.69) | 2.922 (2.71, 3.13) |
|  | GLM | 2.725 (2.64, 2.81) | 2.561 (2.5, 2.63) | 2.77 (2.59, 2.95) |
|  | GLM-sofa | 2.892 (2.8, 2.98) | 2.731 (2.64, 2.82) | 2.944 (2.77, 3.12) |
|  | GLM-saps | 3.122 (3.03, 3.21) | 2.961 (2.83, 3.09) | 3.188 (3.01, 3.36) |
|  | TMLE-dmg | 2.767 (2.7, 2.83) | 2.617 (2.55, 2.68) | 2.952 (2.71, 3.19) |
|  | TMLE-dmg_saps | 2.756 (2.69, 2.82) | 2.625 (2.56, 2.69) | 2.961 (2.72, 3.2) |
| h_m_RR | [prm]TMLE-dmg_sofa | 2.449 (2.39, 2.51) | 2.33 (2.26, 2.4) | 2.672 (2.45, 2.9) |
|  | GLM | 2.426 (2.34, 2.52) | 2.268 (2.2, 2.33) | 2.544 (2.37, 2.72) |
|  | GLM-sofa | 2.614 (2.52, 2.7) | 2.458 (2.37, 2.55) | 2.738 (2.56, 2.91) |
|  | GLM-saps | 2.941 (2.85, 3.03) | 2.785 (2.66, 2.91) | 3.084 (2.91, 3.26) |
|  | TMLE-dmg | 2.455 (2.39, 2.52) | 2.33 (2.26, 2.4) | 2.662 (2.45, 2.87) |
|  | TMLE-dmg_saps | 2.442 (2.38, 2.5) | 2.337 (2.27, 2.41) | 2.693 (2.46, 2.93) |
| h_m_TempC | [prm]TMLE-dmg_sofa | 14.542 (14.44, 14.65) | 15.186 (14.97, 15.4) | 15.304 (15.01, 15.6) |
|  | GLM | 14.293 (14.11, 14.48) | 15.195 (15.06, 15.33) | 15.118 (14.76, 15.48) |
|  | GLM-sofa | 15.705 (15.52, 15.89) | 16.631 (16.44, 16.82) | 16.583 (16.22, 16.94) |
|  | GLM-saps | 14.975 (14.79, 15.16) | 15.881 (15.62, 16.14) | 15.834 (15.47, 16.2) |
|  | TMLE-dmg | 14.585 (14.48, 14.69) | 15.167 (14.94, 15.39) | 15.307 (15, 15.62) |
|  | TMLE-dmg_saps | 14.578 (14.47, 14.69) | 15.154 (14.94, 15.36) | 15.34 (15.02, 15.66) |

####

#### Appendix G.6.3. Estimated Average Measurement Frequencies for Lab Tests across Partner Groups

| **Y** | **method** | **With partner** | **No partner** | **Missing** |
| --- | --- | --- | --- | --- |
| n_HCT | [prm]TMLE-dmg_sofa | 2.784 (2.75, 2.81) | 2.687 (2.64, 2.74) | 2.503 (2.43, 2.58) |
|  | GLM | 2.784 (2.74, 2.83) | 2.66 (2.63, 2.7) | 2.586 (2.49, 2.68) |
|  | GLM-sofa | 2.116 (2.07, 2.16) | 1.981 (1.93, 2.03) | 1.893 (1.8, 1.98) |
|  | GLM-saps | 2.193 (2.15, 2.24) | 2.066 (2, 2.13) | 1.965 (1.87, 2.06) |
|  | TMLE-dmg | 2.763 (2.73, 2.79) | 2.713 (2.66, 2.76) | 2.523 (2.44, 2.61) |
|  | TMLE-dmg_saps | 2.776 (2.75, 2.81) | 2.694 (2.65, 2.74) | 2.486 (2.4, 2.57) |
| n_Lac | [prm]TMLE-dmg_sofa | 1.578 (1.54, 1.62) | 1.593 (1.55, 1.64) | 1.625 (1.52, 1.73) |
|  | GLM | 1.501 (1.44, 1.56) | 1.598 (1.55, 1.64) | 1.723 (1.6, 1.84) |
|  | GLM-sofa | 0.079 (0.03, 0.13) | 0.152 (0.1, 0.21) | 0.248 (0.14, 0.35) |
|  | GLM-saps | -0.3 (-0.36, -0.24) | -0.215 (-0.3, -0.13) | -0.169 (-0.28, -0.06) |
|  | TMLE-dmg | 1.541 (1.5, 1.58) | 1.635 (1.59, 1.68) | 1.669 (1.55, 1.79) |
|  | TMLE-dmg_saps | 1.579 (1.54, 1.62) | 1.595 (1.55, 1.64) | 1.552 (1.44, 1.66) |
| n_lab_grp1 | [prm]TMLE-dmg_sofa | 0.469 (0.45, 0.48) | 0.514 (0.5, 0.53) | 0.51 (0.47, 0.55) |
|  | GLM | 0.44 (0.42, 0.46) | 0.524 (0.51, 0.54) | 0.534 (0.49, 0.58) |
|  | GLM-sofa | 0.016 (0, 0.04) | 0.093 (0.07, 0.11) | 0.095 (0.05, 0.14) |
|  | GLM-saps | -0.006 (-0.03, 0.02) | 0.076 (0.04, 0.11) | 0.066 (0.02, 0.11) |
|  | TMLE-dmg | 0.457 (0.44, 0.47) | 0.527 (0.51, 0.54) | 0.523 (0.48, 0.56) |
|  | TMLE-dmg_saps | 0.469 (0.45, 0.48) | 0.515 (0.5, 0.53) | 0.497 (0.46, 0.54) |
| n_lab_grp2 | [prm]TMLE-dmg_sofa | 4.51 (4.44, 4.58) | 4.097 (4.02, 4.17) | 4.257 (4.08, 4.43) |
|  | GLM | 4.558 (4.45, 4.66) | 4.003 (3.93, 4.08) | 4.333 (4.13, 4.54) |
|  | GLM-sofa | 2.218 (2.12, 2.31) | 1.624 (1.53, 1.72) | 1.905 (1.72, 2.09) |
|  | GLM-saps | 2.219 (2.12, 2.32) | 1.649 (1.5, 1.79) | 1.876 (1.68, 2.08) |
|  | TMLE-dmg | 4.438 (4.37, 4.51) | 4.167 (4.09, 4.25) | 4.33 (4.14, 4.53) |
|  | TMLE-dmg_saps | 4.482 (4.41, 4.55) | 4.125 (4.05, 4.2) | 4.184 (4, 4.37) |
| n_lab_grp3 | [prm]TMLE-dmg_sofa | 1.965 (1.95, 1.98) | 2.002 (1.98, 2.02) | 2.054 (2, 2.11) |
|  | GLM | 1.921 (1.89, 1.95) | 2.019 (2, 2.04) | 2.086 (2.03, 2.14) |
|  | GLM-sofa | 1.414 (1.39, 1.44) | 1.503 (1.48, 1.53) | 1.56 (1.51, 1.61) |
|  | GLM-saps | 1.205 (1.18, 1.23) | 1.298 (1.26, 1.34) | 1.334 (1.28, 1.39) |
|  | TMLE-dmg | 1.951 (1.93, 1.97) | 2.019 (2, 2.04) | 2.074 (2.01, 2.14) |
|  | TMLE-dmg_saps | 1.968 (1.95, 1.99) | 2.003 (1.98, 2.02) | 2.018 (1.96, 2.07) |
| n_lab_grp4 | [prm]TMLE-dmg_sofa | 2.017 (2, 2.04) | 1.982 (1.96, 2) | 1.856 (1.8, 1.91) |
|  | GLM | 2.005 (1.98, 2.04) | 1.973 (1.95, 1.99) | 1.887 (1.83, 1.95) |
|  | GLM-sofa | 1.442 (1.41, 1.47) | 1.4 (1.37, 1.43) | 1.303 (1.25, 1.36) |
|  | GLM-saps | 1.433 (1.4, 1.46) | 1.396 (1.35, 1.44) | 1.286 (1.23, 1.34) |
|  | TMLE-dmg | 2.001 (1.98, 2.02) | 2 (1.98, 2.02) | 1.876 (1.81, 1.94) |
|  | TMLE-dmg_saps | 2.015 (1.99, 2.04) | 1.987 (1.96, 2.01) | 1.836 (1.77, 1.9) |
| n_lab_grp5 | [prm]TMLE-dmg_sofa | 0.278 (0.27, 0.29) | 0.305 (0.29, 0.32) | 0.273 (0.25, 0.3) |
|  | GLM | 0.263 (0.25, 0.28) | 0.31 (0.3, 0.32) | 0.274 (0.24, 0.3) |
|  | GLM-sofa | 0.108 (0.09, 0.12) | 0.153 (0.14, 0.17) | 0.113 (0.08, 0.14) |
|  | GLM-saps | 0.018 (0, 0.03) | 0.063 (0.04, 0.08) | 0.016 (-0.01, 0.04) |
|  | TMLE-dmg | 0.274 (0.26, 0.28) | 0.309 (0.3, 0.32) | 0.277 (0.25, 0.3) |
|  | TMLE-dmg_saps | 0.279 (0.27, 0.29) | 0.305 (0.29, 0.32) | 0.262 (0.24, 0.29) |
| n_lab_grp6 | [prm]TMLE-dmg_sofa | 1.552 (1.53, 1.57) | 1.642 (1.62, 1.66) | 1.748 (1.69, 1.81) |
|  | GLM | 1.488 (1.46, 1.52) | 1.677 (1.65, 1.7) | 1.787 (1.73, 1.85) |
|  | GLM-sofa | 1.047 (1.02, 1.08) | 1.229 (1.2, 1.26) | 1.33 (1.27, 1.39) |
|  | GLM-saps | 0.815 (0.78, 0.84) | 0.999 (0.96, 1.04) | 1.08 (1.02, 1.14) |
|  | TMLE-dmg | 1.539 (1.52, 1.56) | 1.656 (1.63, 1.68) | 1.768 (1.71, 1.83) |
|  | TMLE-dmg_saps | 1.555 (1.53, 1.58) | 1.64 (1.62, 1.66) | 1.718 (1.66, 1.78) |
| n_lab_grp7 | [prm]TMLE-dmg_sofa | 1.75 (1.73, 1.77) | 1.682 (1.66, 1.71) | 1.689 (1.62, 1.76) |
|  | GLM | 1.74 (1.7, 1.78) | 1.674 (1.65, 1.7) | 1.75 (1.68, 1.82) |
|  | GLM-sofa | 1.09 (1.06, 1.13) | 1.013 (0.98, 1.05) | 1.076 (1.01, 1.15) |
|  | GLM-saps | 1.09 (1.05, 1.13) | 1.019 (0.97, 1.07) | 1.067 (0.99, 1.14) |
|  | TMLE-dmg | 1.732 (1.71, 1.76) | 1.703 (1.67, 1.73) | 1.71 (1.64, 1.78) |
|  | TMLE-dmg_saps | 1.748 (1.72, 1.77) | 1.688 (1.66, 1.72) | 1.676 (1.61, 1.74) |

#### Appendix G.7.1. Estimated Average Measurement Frequencies for Vital Signs across Religion Groups

| **Y** | **method** | **Christian** | **Other** | **Missing** |
| --- | --- | --- | --- | --- |
| n_BP | [prm]TMLE-dmg_sofa | 34.551 (34.26, 34.84) | 34.513 (34.08, 34.95) | 33.94 (33.66, 34.22) |
|  | GLM | 34.266 (34.02, 34.52) | 34.288 (33.77, 34.81) | 33.482 (33.09, 33.88) |
|  | GLM-sofa | 29.432 (29.08, 29.79) | 29.418 (28.91, 29.92) | 28.769 (28.38, 29.15) |
|  | GLM-saps | 26.87 (26.35, 27.39) | 26.595 (26.09, 27.1) | 26.327 (25.94, 26.71) |
|  | TMLE-dmg | 34.554 (34.27, 34.84) | 34.515 (34.06, 34.97) | 33.94 (33.65, 34.23) |
|  | TMLE-dmg_saps | 34.541 (34.26, 34.82) | 34.573 (34.13, 35.01) | 33.933 (33.65, 34.22) |
| n_BPm | [prm]TMLE-dmg_sofa | 34.351 (34.06, 34.64) | 34.324 (33.89, 34.76) | 33.789 (33.51, 34.07) |
|  | GLM | 34.062 (33.81, 34.31) | 34.12 (33.6, 34.64) | 33.318 (32.93, 33.71) |
|  | GLM-sofa | 29.102 (28.75, 29.46) | 29.123 (28.62, 29.62) | 28.482 (28.1, 28.86) |
|  | GLM-saps | 26.435 (25.92, 26.95) | 26.185 (25.68, 26.69) | 25.939 (25.55, 26.32) |
|  | TMLE-dmg | 34.357 (34.08, 34.64) | 34.321 (33.87, 34.78) | 33.785 (33.5, 34.07) |
|  | TMLE-dmg_saps | 34.34 (34.06, 34.62) | 34.376 (33.94, 34.82) | 33.78 (33.5, 34.06) |
| n_GCS | [prm]TMLE-dmg_sofa | 3.006 (2.92, 3.1) | 2.333 (2.19, 2.48) | 3.593 (3.47, 3.71) |
|  | GLM | 2.967 (2.88, 3.06) | 2.372 (2.19, 2.56) | 3.63 (3.49, 3.77) |
|  | GLM-sofa | 3.338 (3.21, 3.47) | 2.744 (2.56, 2.93) | 3.991 (3.85, 4.13) |
|  | GLM-saps | 3.149 (2.96, 3.34) | 2.561 (2.38, 2.75) | 3.805 (3.67, 3.95) |
|  | TMLE-dmg | 3.002 (2.91, 3.09) | 2.344 (2.2, 2.49) | 3.603 (3.48, 3.72) |
|  | TMLE-dmg_saps | 2.997 (2.91, 3.09) | 2.336 (2.19, 2.48) | 3.602 (3.48, 3.72) |
| n_GCS_Total | [prm]TMLE-dmg_sofa | 4.902 (4.79, 5.01) | 5.617 (5.44, 5.79) | 5.004 (4.88, 5.13) |
|  | GLM | 4.856 (4.76, 4.95) | 5.429 (5.22, 5.63) | 5.03 (4.87, 5.19) |
|  | GLM-sofa | 5.745 (5.6, 5.89) | 6.325 (6.12, 6.53) | 5.897 (5.74, 6.05) |
|  | GLM-saps | 5.97 (5.76, 6.18) | 6.588 (6.38, 6.79) | 6.108 (5.95, 6.26) |
|  | TMLE-dmg | 4.888 (4.78, 4.99) | 5.613 (5.43, 5.79) | 5.015 (4.89, 5.14) |
|  | TMLE-dmg_saps | 4.899 (4.79, 5) | 5.599 (5.42, 5.78) | 5.017 (4.89, 5.14) |
| n_HR | [prm]TMLE-dmg_sofa | 31.425 (31.2, 31.65) | 31.36 (31.02, 31.7) | 30.834 (30.63, 31.04) |
|  | GLM | 31.184 (30.99, 31.37) | 31.174 (30.78, 31.57) | 30.455 (30.16, 30.75) |
|  | GLM-sofa | 27.617 (27.35, 27.89) | 27.581 (27.2, 27.96) | 26.977 (26.69, 27.27) |
|  | GLM-saps | 25.875 (25.48, 26.27) | 25.651 (25.26, 26.04) | 25.318 (25.02, 25.61) |
|  | TMLE-dmg | 31.429 (31.21, 31.65) | 31.342 (30.99, 31.69) | 30.83 (30.62, 31.04) |
|  | TMLE-dmg_saps | 31.423 (31.21, 31.64) | 31.394 (31.05, 31.74) | 30.818 (30.61, 31.03) |
| n_SpO2 | [prm]TMLE-dmg_sofa | 30.85 (30.63, 31.07) | 30.746 (30.41, 31.09) | 30.402 (30.19, 30.61) |
|  | GLM | 30.65 (30.46, 30.84) | 30.585 (30.19, 30.98) | 30.118 (29.82, 30.42) |
|  | GLM-sofa | 27.371 (27.1, 27.64) | 27.282 (26.89, 27.67) | 26.921 (26.63, 27.21) |
|  | GLM-saps | 26.053 (25.66, 26.45) | 25.803 (25.41, 26.19) | 25.67 (25.37, 25.97) |
|  | TMLE-dmg | 30.845 (30.63, 31.06) | 30.73 (30.37, 31.09) | 30.392 (30.18, 30.6) |
|  | TMLE-dmg_saps | 30.839 (30.63, 31.05) | 30.772 (30.42, 31.12) | 30.394 (30.18, 30.61) |
| n_RR | [prm]TMLE-dmg_sofa | 34.835 (34.58, 35.09) | 34.18 (33.79, 34.57) | 34.858 (34.61, 35.11) |
|  | GLM | 34.575 (34.35, 34.8) | 33.918 (33.45, 34.38) | 34.504 (34.15, 34.86) |
|  | GLM-sofa | 29.032 (28.72, 29.34) | 28.334 (27.89, 28.78) | 29.1 (28.76, 29.44) |
|  | GLM-saps | 25.971 (25.52, 26.43) | 24.967 (24.52, 25.42) | 26.18 (25.84, 26.52) |
|  | TMLE-dmg | 34.847 (34.59, 35.1) | 34.146 (33.74, 34.55) | 34.84 (34.59, 35.1) |
|  | TMLE-dmg_saps | 34.832 (34.58, 35.08) | 34.258 (33.86, 34.65) | 34.811 (34.56, 35.06) |
| n_TempC | [prm]TMLE-dmg_sofa | 12.118 (11.92, 12.32) | 12.592 (12.21, 12.98) | 11.425 (11.19, 11.66) |
|  | GLM | 12.157 (11.96, 12.35) | 12.412 (12.01, 12.82) | 11.212 (10.9, 11.52) |
|  | GLM-sofa | 9.713 (9.43, 10) | 9.95 (9.55, 10.35) | 8.83 (8.52, 9.13) |
|  | GLM-saps | 11.144 (10.73, 11.56) | 11.359 (10.95, 11.76) | 10.233 (9.92, 10.54) |
|  | TMLE-dmg | 12.13 (11.93, 12.33) | 12.595 (12.2, 12.99) | 11.405 (11.17, 11.64) |
|  | TMLE-dmg_saps | 12.104 (11.9, 12.31) | 12.634 (12.24, 13.03) | 11.415 (11.18, 11.65) |

####

#### Appendix G.7.2. Estimated Average Missing Data Rates for Vital Signs across Religion Groups

| **Y** | **method** | **Christian** | **Other** | **Missing** |
| --- | --- | --- | --- | --- |
| h_m_BP | [prm]TMLE-dmg_sofa | 2.273 (2.22, 2.32) | 2.47 (2.37, 2.57) | 2.345 (2.28, 2.41) |
|  | GLM | 2.236 (2.18, 2.29) | 2.402 (2.3, 2.51) | 2.32 (2.24, 2.4) |
|  | GLM-sofa | 2.548 (2.47, 2.62) | 2.716 (2.61, 2.82) | 2.624 (2.54, 2.7) |
|  | GLM-saps | 2.964 (2.86, 3.07) | 3.159 (3.05, 3.26) | 3.024 (2.94, 3.1) |
|  | TMLE-dmg | 2.268 (2.22, 2.32) | 2.478 (2.38, 2.58) | 2.348 (2.29, 2.41) |
|  | TMLE-dmg_saps | 2.269 (2.22, 2.32) | 2.465 (2.36, 2.57) | 2.346 (2.28, 2.41) |
| h_m_BPm | [prm]TMLE-dmg_sofa | 2.363 (2.31, 2.42) | 2.553 (2.45, 2.66) | 2.413 (2.35, 2.48) |
|  | GLM | 2.327 (2.27, 2.38) | 2.476 (2.37, 2.59) | 2.392 (2.31, 2.47) |
|  | GLM-sofa | 2.665 (2.59, 2.74) | 2.817 (2.71, 2.93) | 2.722 (2.64, 2.8) |
|  | GLM-saps | 3.108 (3, 3.22) | 3.289 (3.18, 3.4) | 3.148 (3.07, 3.23) |
|  | TMLE-dmg | 2.359 (2.31, 2.41) | 2.561 (2.46, 2.67) | 2.416 (2.35, 2.48) |
|  | TMLE-dmg_saps | 2.36 (2.31, 2.41) | 2.547 (2.44, 2.65) | 2.414 (2.35, 2.48) |
| h_m_GCS | [prm]TMLE-dmg_sofa | 21.214 (21.05, 21.38) | 21.816 (21.67, 21.96) | 20.607 (20.49, 20.72) |
|  | GLM | 21.12 (21, 21.24) | 21.664 (21.42, 21.91) | 20.459 (20.28, 20.64) |
|  | GLM-sofa | 20.851 (20.68, 21.02) | 21.393 (21.15, 21.64) | 20.197 (20.01, 20.38) |
|  | GLM-saps | 21.014 (20.77, 21.26) | 21.554 (21.31, 21.8) | 20.356 (20.17, 20.54) |
|  | TMLE-dmg | 21.201 (21.05, 21.35) | 21.8 (21.65, 21.95) | 20.603 (20.49, 20.72) |
|  | TMLE-dmg_saps | 21.212 (21.06, 21.36) | 21.797 (21.65, 21.95) | 20.602 (20.48, 20.72) |
| h_m_GCS_Total | [prm]TMLE-dmg_sofa | 19.298 (19.18, 19.42) | 18.538 (18.36, 18.71) | 19.152 (19.03, 19.27) |
|  | GLM | 19.212 (19.11, 19.32) | 18.602 (18.39, 18.82) | 19.013 (18.85, 19.17) |
|  | GLM-sofa | 18.405 (18.26, 18.55) | 17.789 (17.58, 18) | 18.226 (18.06, 18.39) |
|  | GLM-saps | 18.162 (17.95, 18.38) | 17.509 (17.3, 17.72) | 17.996 (17.83, 18.16) |
|  | TMLE-dmg | 19.295 (19.18, 19.41) | 18.528 (18.35, 18.7) | 19.148 (19.02, 19.27) |
|  | TMLE-dmg_saps | 19.286 (19.17, 19.4) | 18.546 (18.37, 18.72) | 19.143 (19.02, 19.27) |
| h_m_HR | [prm]TMLE-dmg_sofa | 2.047 (2, 2.1) | 2.25 (2.15, 2.35) | 2.099 (2.04, 2.16) |
|  | GLM | 2.015 (1.96, 2.06) | 2.172 (2.07, 2.28) | 2.077 (2, 2.16) |
|  | GLM-sofa | 2.217 (2.14, 2.29) | 2.376 (2.27, 2.48) | 2.275 (2.2, 2.35) |
|  | GLM-saps | 2.606 (2.5, 2.71) | 2.787 (2.68, 2.89) | 2.65 (2.57, 2.73) |
|  | TMLE-dmg | 2.043 (1.99, 2.09) | 2.259 (2.16, 2.36) | 2.102 (2.04, 2.16) |
|  | TMLE-dmg_saps | 2.045 (2, 2.09) | 2.247 (2.15, 2.35) | 2.1 (2.04, 2.16) |
| h_m_SpO2 | [prm]TMLE-dmg_sofa | 2.674 (2.61, 2.73) | 2.967 (2.85, 3.08) | 2.651 (2.58, 2.72) |
|  | GLM | 2.636 (2.58, 2.7) | 2.862 (2.74, 2.99) | 2.598 (2.5, 2.69) |
|  | GLM-sofa | 2.809 (2.72, 2.9) | 3.036 (2.91, 3.16) | 2.767 (2.67, 2.86) |
|  | GLM-saps | 3.051 (2.92, 3.18) | 3.294 (3.17, 3.42) | 3 (2.91, 3.09) |
|  | TMLE-dmg | 2.674 (2.61, 2.73) | 2.971 (2.85, 3.09) | 2.654 (2.58, 2.73) |
|  | TMLE-dmg_saps | 2.674 (2.61, 2.73) | 2.967 (2.85, 3.09) | 2.649 (2.58, 2.72) |
| h_m_RR | [prm]TMLE-dmg_sofa | 2.363 (2.3, 2.42) | 2.703 (2.58, 2.83) | 2.348 (2.28, 2.42) |
|  | GLM | 2.321 (2.26, 2.38) | 2.622 (2.5, 2.75) | 2.32 (2.23, 2.41) |
|  | GLM-sofa | 2.513 (2.43, 2.6) | 2.816 (2.69, 2.94) | 2.508 (2.41, 2.6) |
|  | GLM-saps | 2.854 (2.73, 2.98) | 3.177 (3.05, 3.3) | 2.836 (2.74, 2.93) |
|  | TMLE-dmg | 2.36 (2.3, 2.42) | 2.713 (2.58, 2.84) | 2.349 (2.28, 2.42) |
|  | TMLE-dmg_saps | 2.36 (2.3, 2.42) | 2.701 (2.57, 2.83) | 2.349 (2.28, 2.42) |
| h_m_TempC | [prm]TMLE-dmg_sofa | 14.736 (14.58, 14.89) | 14.56 (14.36, 14.76) | 15.036 (14.91, 15.16) |
|  | GLM | 14.608 (14.48, 14.73) | 14.555 (14.3, 14.81) | 15.008 (14.81, 15.2) |
|  | GLM-sofa | 16.028 (15.85, 16.21) | 15.986 (15.73, 16.24) | 16.393 (16.2, 16.59) |
|  | GLM-saps | 15.253 (14.99, 15.51) | 15.226 (14.97, 15.48) | 15.632 (15.44, 15.83) |
|  | TMLE-dmg | 14.713 (14.56, 14.86) | 14.549 (14.34, 14.75) | 15.054 (14.93, 15.18) |
|  | TMLE-dmg_saps | 14.723 (14.57, 14.87) | 14.531 (14.33, 14.73) | 15.049 (14.92, 15.18) |

####

#### Appendix G.7.3. Estimated Average Measurement Frequencies for Lab Tests across Religion Groups

| **Y** | **method** | **Christian** | **Other** | **Missing** |
| --- | --- | --- | --- | --- |
| n_HCT | [prm]TMLE-dmg_sofa | 2.769 (2.73, 2.81) | 2.69 (2.64, 2.74) | 2.722 (2.69, 2.76) |
|  | GLM | 2.745 (2.71, 2.78) | 2.639 (2.57, 2.71) | 2.711 (2.66, 2.76) |
|  | GLM-sofa | 2.067 (2.02, 2.11) | 1.956 (1.89, 2.02) | 2.05 (2, 2.1) |
|  | GLM-saps | 2.147 (2.08, 2.21) | 2.017 (1.95, 2.08) | 2.133 (2.08, 2.18) |
|  | TMLE-dmg | 2.77 (2.73, 2.81) | 2.684 (2.63, 2.74) | 2.721 (2.68, 2.76) |
|  | TMLE-dmg_saps | 2.77 (2.73, 2.81) | 2.695 (2.64, 2.75) | 2.719 (2.68, 2.75) |
| n_Lac | [prm]TMLE-dmg_sofa | 1.576 (1.54, 1.62) | 1.492 (1.42, 1.56) | 1.634 (1.59, 1.68) |
|  | GLM | 1.553 (1.51, 1.59) | 1.427 (1.34, 1.51) | 1.622 (1.56, 1.69) |
|  | GLM-sofa | 0.104 (0.05, 0.16) | -0.033 (-0.11, 0.04) | 0.208 (0.15, 0.27) |
|  | GLM-saps | -0.288 (-0.37, -0.21) | -0.488 (-0.57, -0.41) | -0.159 (-0.22, -0.1) |
|  | TMLE-dmg | 1.585 (1.54, 1.63) | 1.482 (1.41, 1.56) | 1.625 (1.57, 1.68) |
|  | TMLE-dmg_saps | 1.58 (1.54, 1.62) | 1.515 (1.44, 1.59) | 1.618 (1.57, 1.67) |
| n_lab_grp1 | [prm]TMLE-dmg_sofa | 0.493 (0.48, 0.51) | 0.456 (0.43, 0.48) | 0.509 (0.49, 0.53) |
|  | GLM | 0.481 (0.47, 0.5) | 0.419 (0.39, 0.45) | 0.513 (0.49, 0.54) |
|  | GLM-sofa | 0.048 (0.03, 0.07) | -0.017 (-0.05, 0.01) | 0.091 (0.07, 0.11) |
|  | GLM-saps | 0.022 (-0.01, 0.05) | -0.058 (-0.09, -0.03) | 0.069 (0.05, 0.09) |
|  | TMLE-dmg | 0.498 (0.48, 0.51) | 0.452 (0.43, 0.48) | 0.507 (0.49, 0.52) |
|  | TMLE-dmg_saps | 0.496 (0.48, 0.51) | 0.458 (0.43, 0.48) | 0.504 (0.49, 0.52) |
| n_lab_grp2 | [prm]TMLE-dmg_sofa | 4.403 (4.33, 4.47) | 4.415 (4.3, 4.54) | 4.221 (4.14, 4.3) |
|  | GLM | 4.411 (4.34, 4.48) | 4.279 (4.13, 4.42) | 4.158 (4.05, 4.27) |
|  | GLM-sofa | 2.038 (1.94, 2.13) | 1.889 (1.76, 2.02) | 1.845 (1.74, 1.95) |
|  | GLM-saps | 2.054 (1.91, 2.2) | 1.828 (1.69, 1.97) | 1.878 (1.77, 1.99) |
|  | TMLE-dmg | 4.41 (4.34, 4.48) | 4.415 (4.28, 4.55) | 4.209 (4.12, 4.29) |
|  | TMLE-dmg_saps | 4.404 (4.33, 4.47) | 4.457 (4.33, 4.58) | 4.209 (4.13, 4.29) |
| n_lab_grp3 | [prm]TMLE-dmg_sofa | 1.966 (1.95, 1.99) | 1.976 (1.94, 2.01) | 2.026 (2, 2.05) |
|  | GLM | 1.95 (1.93, 1.97) | 1.937 (1.9, 1.98) | 2.027 (2, 2.06) |
|  | GLM-sofa | 1.432 (1.4, 1.46) | 1.415 (1.38, 1.45) | 1.522 (1.49, 1.55) |
|  | GLM-saps | 1.215 (1.17, 1.25) | 1.172 (1.13, 1.21) | 1.315 (1.29, 1.35) |
|  | TMLE-dmg | 1.971 (1.95, 1.99) | 1.973 (1.94, 2.01) | 2.022 (2, 2.05) |
|  | TMLE-dmg_saps | 1.968 (1.95, 1.99) | 1.983 (1.95, 2.02) | 2.018 (1.99, 2.04) |
| n_lab_grp4 | [prm]TMLE-dmg_sofa | 2.014 (1.99, 2.04) | 1.938 (1.9, 1.97) | 1.999 (1.97, 2.02) |
|  | GLM | 2.003 (1.98, 2.02) | 1.904 (1.86, 1.95) | 1.99 (1.96, 2.02) |
|  | GLM-sofa | 1.43 (1.4, 1.46) | 1.327 (1.29, 1.37) | 1.432 (1.4, 1.46) |
|  | GLM-saps | 1.42 (1.38, 1.46) | 1.299 (1.26, 1.34) | 1.427 (1.4, 1.46) |
|  | TMLE-dmg | 2.018 (2, 2.04) | 1.932 (1.89, 1.97) | 1.996 (1.97, 2.02) |
|  | TMLE-dmg_saps | 2.016 (1.99, 2.04) | 1.941 (1.9, 1.98) | 1.992 (1.97, 2.02) |
| n_lab_grp5 | [prm]TMLE-dmg_sofa | 0.291 (0.28, 0.3) | 0.265 (0.25, 0.28) | 0.293 (0.28, 0.31) |
|  | GLM | 0.286 (0.28, 0.3) | 0.266 (0.25, 0.29) | 0.289 (0.27, 0.3) |
|  | GLM-sofa | 0.128 (0.11, 0.14) | 0.107 (0.09, 0.13) | 0.135 (0.12, 0.15) |
|  | GLM-saps | 0.036 (0.02, 0.06) | 0.006 (-0.01, 0.03) | 0.047 (0.03, 0.06) |
|  | TMLE-dmg | 0.292 (0.28, 0.3) | 0.262 (0.24, 0.28) | 0.292 (0.28, 0.31) |
|  | TMLE-dmg_saps | 0.292 (0.28, 0.3) | 0.266 (0.25, 0.28) | 0.29 (0.28, 0.3) |
| n_lab_grp6 | [prm]TMLE-dmg_sofa | 1.566 (1.55, 1.59) | 1.602 (1.56, 1.64) | 1.655 (1.63, 1.68) |
|  | GLM | 1.546 (1.53, 1.57) | 1.564 (1.52, 1.61) | 1.665 (1.63, 1.7) |
|  | GLM-sofa | 1.094 (1.06, 1.12) | 1.108 (1.07, 1.15) | 1.223 (1.19, 1.26) |
|  | GLM-saps | 0.851 (0.81, 0.89) | 0.841 (0.8, 0.88) | 0.992 (0.96, 1.02) |
|  | TMLE-dmg | 1.571 (1.55, 1.59) | 1.598 (1.56, 1.64) | 1.652 (1.63, 1.68) |
|  | TMLE-dmg_saps | 1.569 (1.55, 1.59) | 1.606 (1.57, 1.65) | 1.648 (1.62, 1.67) |
| n_lab_grp7 | [prm]TMLE-dmg_sofa | 1.729 (1.7, 1.75) | 1.713 (1.67, 1.76) | 1.728 (1.7, 1.76) |
|  | GLM | 1.72 (1.69, 1.74) | 1.676 (1.62, 1.73) | 1.718 (1.68, 1.76) |
|  | GLM-sofa | 1.059 (1.02, 1.09) | 1.01 (0.96, 1.06) | 1.073 (1.04, 1.11) |
|  | GLM-saps | 1.058 (1.01, 1.11) | 0.988 (0.94, 1.04) | 1.078 (1.04, 1.12) |
|  | TMLE-dmg | 1.734 (1.71, 1.76) | 1.707 (1.66, 1.76) | 1.723 (1.69, 1.75) |
|  | TMLE-dmg_saps | 1.733 (1.71, 1.76) | 1.716 (1.67, 1.76) | 1.72 (1.69, 1.75) |

#### 
